# Redox deracemization of β,γ-alkynyl α-amino esters†

**DOI:** 10.1039/d0sc00944j

**Published:** 2020-04-21

**Authors:** Lu Zhang, Rongxiu Zhu, Aili Feng, Changyin Zhao, Lei Chen, Guidong Feng, Lei Liu

**Affiliations:** School of Pharmaceutical Sciences, Shandong University Jinan 250012 China leiliu@sdu.edu.cn; School of Chemistry and Chemical Engineering, Shandong University Jinan 250100 China

## Abstract

The first non-enzymatic redox deracemization method using molecular oxygen as the terminal oxidant has been described. The one-pot deracemization of β,γ-alkynyl α-amino esters consisted of a copper-catalyzed aerobic oxidation and chiral phosphoric acid-catalyzed asymmetric transfer hydrogenation with excellent functional group compatibility. By using benzothiazoline as the reducing reagent, an exclusive chemoselectivity at the C=N bond over the C≡C bond was achieved, allowing for efficient deracemization of a series of α-amino esters bearing diverse α-alkynyl substituent patterns. The origins of chemo- and enantio-selectivities were elucidated by experimental and computational mechanistic investigation. The generality of the strategy is further demonstrated by efficient deracemization of β,γ-alkenyl α-amino esters.

## Abstract 2

A one-pot deracemization of β,γ-alkynyl α-amino esters consisting of an aerobic oxidation and chiral phosphoric acid-catalyzed asymmetric transfer hydrogenation has been described.

## Graphical abstract

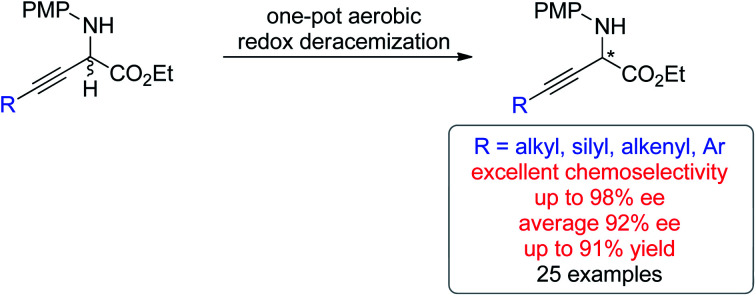

## Introduction

Deracemization, wherein a racemic mixture of a given molecule is completely transformed into a single enantiomer of exactly the same species in theoretically 100% yield, has emerged as an attractive and topologically straightforward alternative to conventional enantioselective synthesis or kinetic resolution strategies.^1–3^ Deracemization is a thermodynamically disfavored process and is commonly achieved by destroying and recreating stereocenters through oxidation and reduction chemistry, at least one of which should involve asymmetric manipulation. Despite the conceptual simplicity and potential practical benefits, pure chemically catalytic redox deracemization has remained underdeveloped^4,5^ and suffers from narrow substrate scope including secondary alcohols,^6^ cyclic amines,^7,8^ and cyclic ethers.^9^ Moreover, existing approaches typically require stoichiometric oxidants such as oxopiperidinium salt, NBS, and DDQ, which generate undesired waste.^6,7,9^ The use of molecular oxygen as an ideal oxidant in light of economic and ecological factors with H_2_O as the only byproduct has never been demonstrated for this process.^10^ Therefore, developing a deracemization method using molecular oxygen as the terminal oxidant to access enantioenriched molecules that are otherwise difficult to access is highly desirable.

**Scheme 1:**
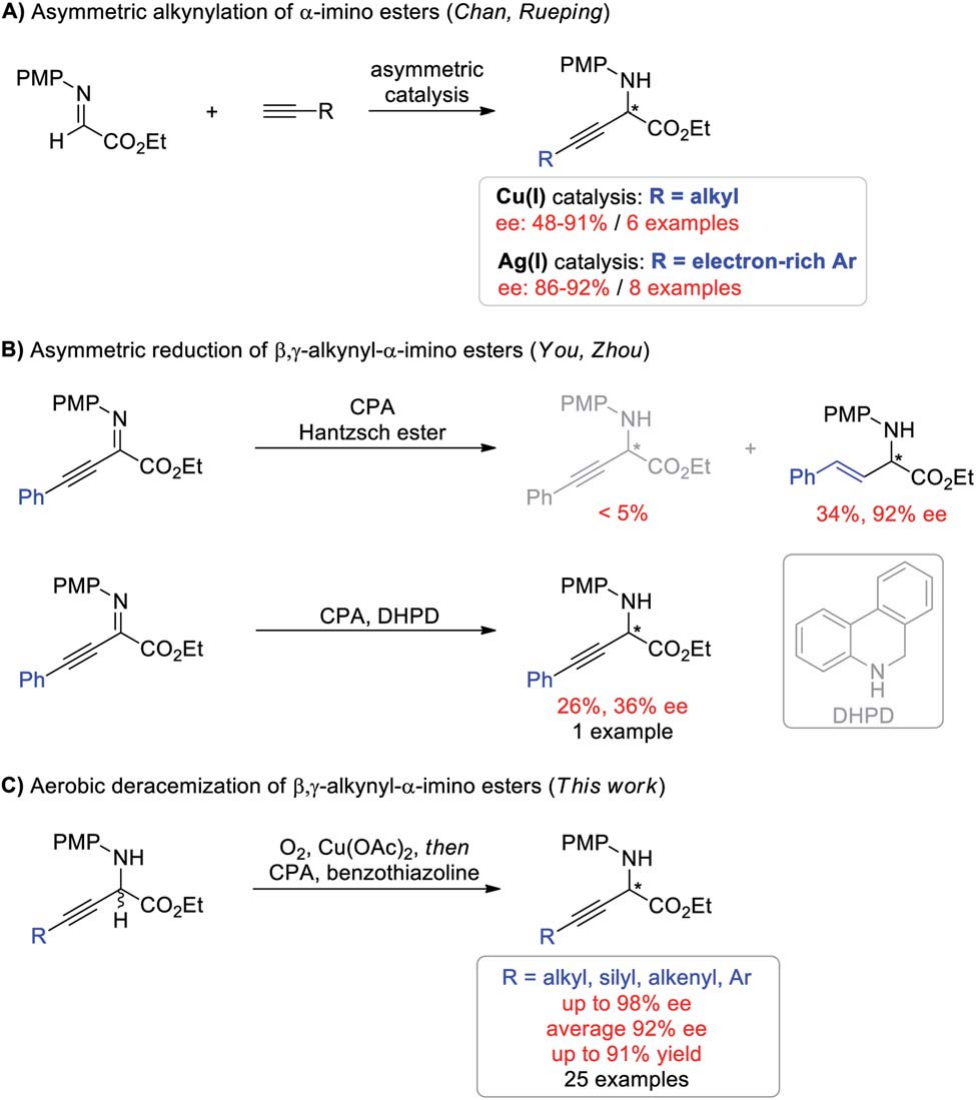
Overview of enantioselective access to β,γ-alkynyl α-amino acid derivatives.

Optically active non-natural amino acids are fundamental subunits for a range of biologically significant molecules.^11,12^ In particular, optically pure β,γ-alkynyl α-amino acids represent a special class of these compounds,^13^ not only because the rich chemistry of alkynes allows for further synthetic elaboration on the β,γ-positions, but also because α-alkynyl moieties can change the biological properties of certain natural amino acids for potential therapeutic utility.^14^ But surprisingly, asymmetric synthesis of β,γ-alkynyl α-amino acids and their derivatives has remained challenging (Scheme 1). The groups of Chan and Rueping demonstrated enantioselective addition of terminal alkynes to α-imino esters (Scheme 1A).^15^ Albeit innovative, each method suffered from a narrow alkyne scope and was merely suitable for either aliphatic or electron-rich aryl alkynes with moderate to good ee (48–92%). Asymmetric hydrogenation of β,γ-alkynyl α-imino esters represents the other potentially efficient strategy.^16^ However, chemo- and enantio-selective reduction of the imine moiety with the alkyne intact proved to be challenging (Scheme 1B). The You group revealed chiral phosphoric acid (CPA) catalyzed asymmetric transfer hydrogenation with a Hantzsch ester, giving β,γ-alkenyl α-amino esters through an initial partial reduction of the C≡C bond followed by C=N bond reduction.^16*a*^ The Zhou group demonstrated that the alkyne group can be conserved using 5,6-dihydrophenanthridine as the hydrogen source, but the expected β,γ-alkynyl α-amino ester was only isolated in 26% yield with 36% ee.^16*b*^ The hydride transfer mode (1,4 or 1,2) and hydrogen transfer ability have been demonstrated to generate a significant influence on the reactivity of target reactions.^17^ Moreover, benzothiazolines have exhibited superior reactivity and enantioselectivity to other hydride donors in several CPA catalyzed transfer hydrogenation reactions by carefully tuning the substituent at the C2 position.^18^ We envisioned that using benzothiazolines as the hydride donor might address the chemo- and enantio-selectivity issues encountered in Scheme 1B. Herein, we report a one-pot redox deracemization of β,γ-alkynyl α-amino acid derivatives (Scheme 1C). The strategically different approach consisted of an aerobic oxidation and CPA-catalyzed asymmetric transfer hydrogenation with benzothiazoline, allowing for asymmetric access to a series of non-natural α-amino esters bearing diverse α-alkynyl substituent patterns in high efficiency with excellent chemo- and enantio-selectivity.

## Results and discussion

Initially, redox deracemization of aliphatic alkynyl substituted α-amino ester rac-**1a** was selected as a model reaction using molecular oxygen as the terminal oxidant and the combination of CPA **2** and benzothiazoline **3** as the asymmetric transfer hydrogenation system (Table 1).^19^ The oxidation was performed prior to the addition of **2** to avoid reagent quenching. No oxidation was observed in the absence of any additive (entry 1, Table 1). A careful examination of metal salt additives revealed that reaction with 10 mol% of Cu(OAc)_2_ provided a clean and efficient aerobic oxidation at rt, and the expected (*S*)-**1a** was recovered in 85% yield with 6% ee in the presence of CPA **2a** and benzothiazoline **3a** (entries 2–7, Table 1). Notably, the reaction exhibited excellent chemoselectivity with the alkynyl moiety highly conserved. The solvent was found to be crucial to the enantioselectivity, and when asymmetric reduction was performed in a mixture of hexane and CH_2_Cl_2_, a remarkably improved ee of 73% was observed (entries 5 and 8, Table 1). The asymmetric transfer hydrogenation system was extensively investigated, and the combination of **2a** and **3d** afforded the highest level of enantiofacial discrimination (entries 8–14, Table 1). Further optimization of the solvent choice identified a mixture of decane and mesitylene to be optimal for the asymmetric reduction process (entries 15 and 16, Table 1). The reaction proved to be sensitive to moisture, as demonstrated by an obvious loss of enantioselectivity in the absence of molecular sieves (entries 16 and 17, Table 1).

**Table 1:** Reaction condition optimization^a^

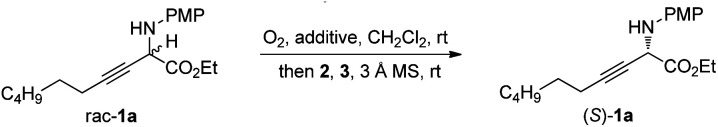					
Entry	Additive	**2**	**3**	Recovery^b^ (%)	ee^c^ (%)
1	—	—	—	n.o.	n.d.
2	CuBr_2_	**2a**	**3a**	<5	n.d.
3	CuCl_2_	**2a**	**3a**	17	3
4	CuF_2_	**2a**	**3a**	63	5
5	Cu(OAc)_2_	**2a**	**3a**	85	6
6	Cu(OTf)_2_	**2a**	**3a**	<5	n.d.
7	CuOAc	**2a**	**3a**	72	5
8^d^	Cu(OAc)_2_	**2a**	**3a**	86	73
9^d^	Cu(OAc)_2_	**2a**	**3b**	73	70
10^d^	Cu(OAc)_2_	**2a**	**3c**	71	47
11^d^	Cu(OAc)_2_	**2a**	**3d**	83	81
12^d^	Cu(OAc)_2_	**2b**	**3d**	76	77
13^d^	Cu(OAc)_2_	**2c**	**3d**	<5	n.d.
14^d^	Cu(OAc)_2_	**2d**	**3d**	70	53
15^e^	Cu(OAc)_2_	**2a**	**3d**	79	85
16^f^	Cu(OAc)_2_	**2a**	**3d**	86	94
17^f^^,^^g^	Cu(OAc)_2_	**2a**	**3d**	79	55
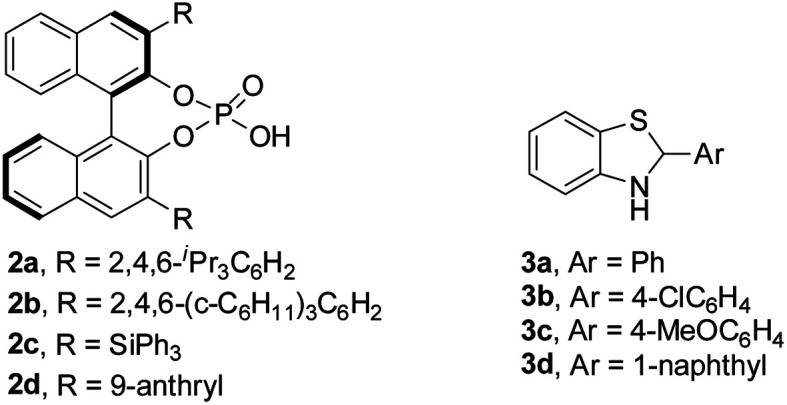					

a Reaction conditions: rac-**1a** (0.1 mmol) and additive (10 mol%) in CH_2_Cl_2_ (2 mL) under 1 atm molecular oxygen at rt for 3 h, followed by **2** (5 mol%), **3** (0.12 mmol), and 3 Å molecular sieves (40 mg) at rt for 4 h.

b Yield of the isolated product.

c Determined by chiral HPLC analysis.

d Asymmetric reduction in a mixture of hexane and CH_2_Cl_2_ (v/v = 1 : 1).

e Asymmetric reduction in a mixture of decane and CH_2_Cl_2_ (v/v = 1 : 1).

f Asymmetric reduction in a mixture of decane and mesitylene (v/v = 1 : 1).

g Reaction without 3 Å MS. PMP = 4-methoxyphenyl, n.o. = no oxidation, n.d. = not determined.

**Scheme 2:**
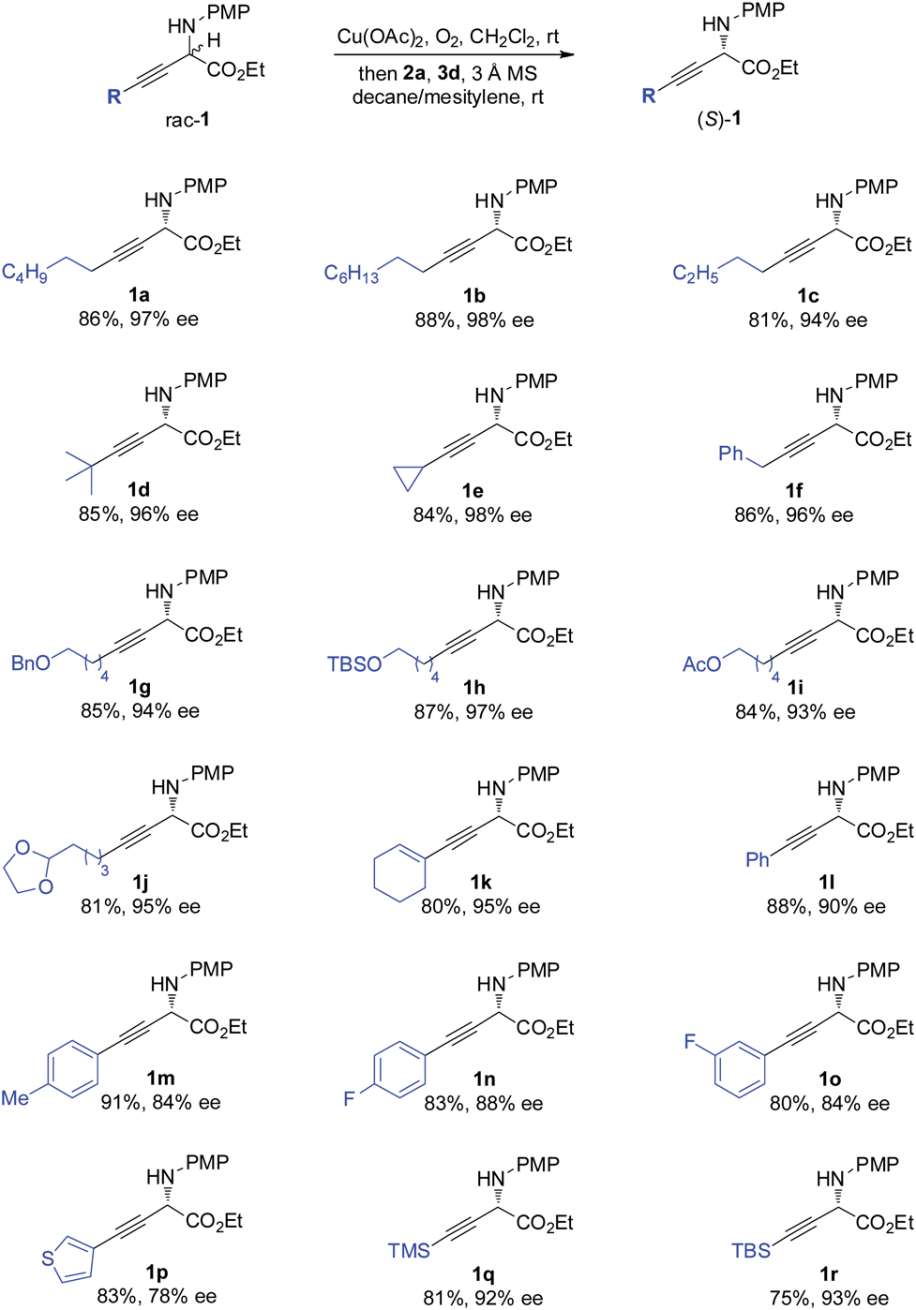
Scope of the α-alkynyl component of non-natural amino esters.

The scope of the one-pot redox deracemization of β,γ-alkynyl α-amino esters was explored (Scheme 2). The reaction was found to be fairly general for substrates bearing a wide range of aliphatic alkynes with varied chain lengths and sizes, affording corresponding optically pure **1a–1d** in good yields with excellent ee (94–98% ee).^20^ The deracemization method exhibited excellent functional group compatibility with commonly encountered ones, including cyclopropane (**1e**), aryl (**1f**), benzyl ether (**1g**), silyl ether (**1h**), acetate (**1i**), and acetal (**1j**), well tolerated as additional functional handles for structurally diverse non-natural amino acid synthesis. Alkenyl substituted alkyne **1k** was similarly effectively deracemized. Substrates containing electronically varied aryl and heteroaryl acetylenes were also suitable candidates for the protocol, as demonstrated by the efficient generation of enantiopure **1l–1p** with high enantiocontrol. Silyl-substituted alkynes were further identified to be competent components for the process, furnishing respective TMS **1q** and TBS **1r** with 92% and 93% ee.

**Scheme 3:**
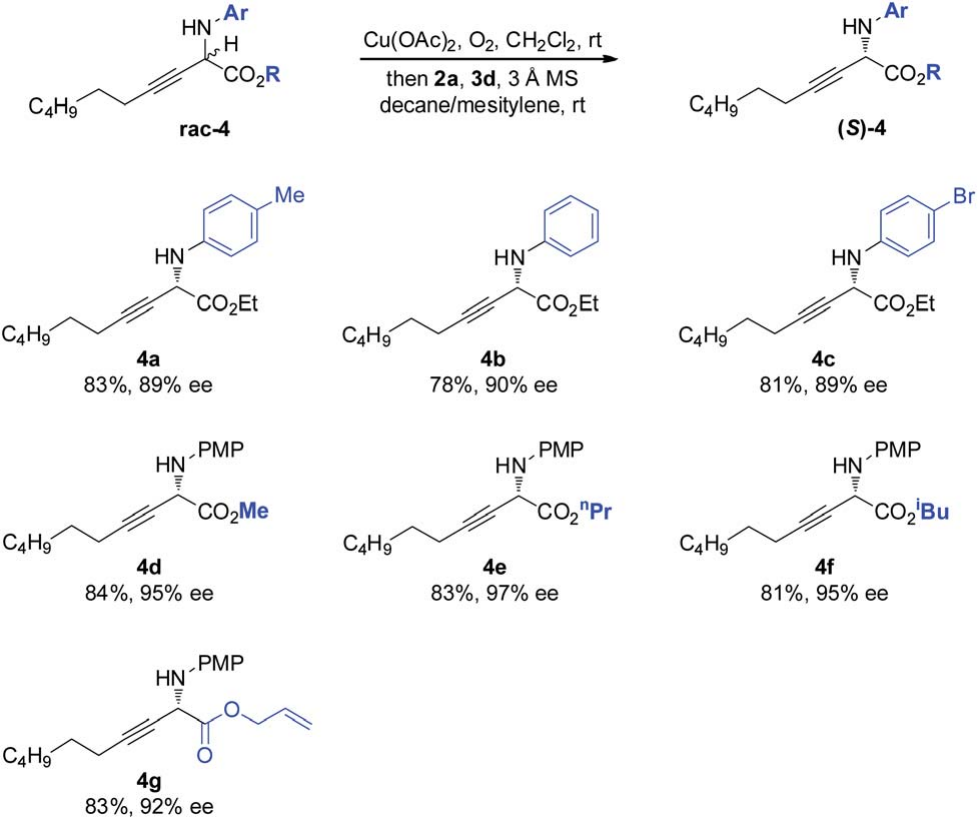
Scope of the amino and ester components of non-natural amino esters.

The scope of the amino component was then investigated (Scheme 3). Besides the electron-rich PMP moiety, other electronically varied aryl groups (**4a–4c**) were also well tolerated. The method was found to be insensitive to the variant on the ester moiety, as demonstrated by effective deracemization of **4d–4g**.

**Scheme 4:**
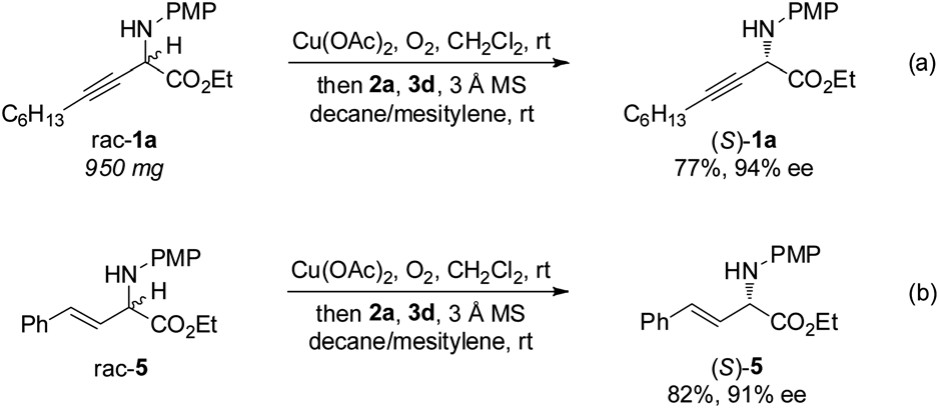
Gram-scale study and redox deracemization of β,γ-alkenyl α-amino ester.

The gram-scale aerobic deracemization proceeded smoothly without obvious loss of efficiency and enantioselectivity, and (*S*)-**1a** was obtained in 77% yield with 94% ee (Scheme 4a). The redox deracemization technology was also applicable to β,γ-alkenyl α-amino esters, as illustrated by the formation of enantiopure (*S*)-**5** in 82% yield with 91% ee (Scheme 4b).^21^

**Scheme 5:**
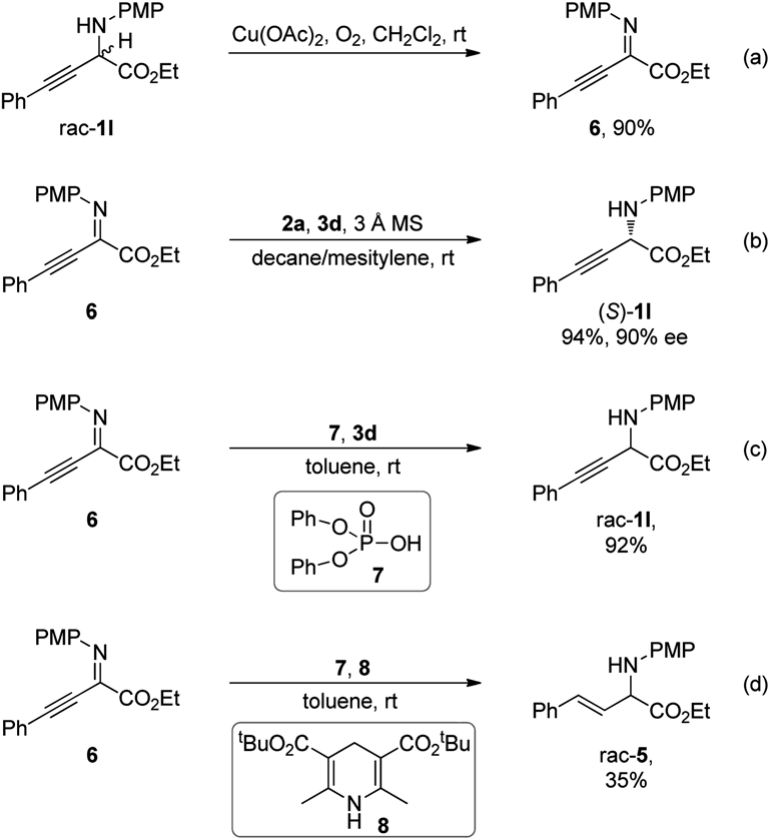
Control experiments.

Control experiments were conducted to gain further insights into the reaction mechanism (Scheme 5). In the presence of a catalytic amount of Cu(OAc)_2_ under a molecular oxygen atmosphere, α-amino ester rac-**1l** was transformed to an intermediate detected by TLC analysis, which was identified as α-imino ester **6** (Scheme 5a). Subjecting **6** to standard conditions in the absence of oxidation elements furnished the desired (*S*)-**1l** with a comparable ee to that obtained by the deracemization process, indicating the intermediacy of α-imino ester **6** (Scheme 5b). Unlike the studies in Scheme 1B, CPA-catalyzed asymmetric transfer hydrogenation of **6** with benzothiazoline as the hydride donor exhibited exclusive chemoselectivity at the C=N bond, and reduction of the C≡C bond was not observed. Diphenyl phosphate **7** catalyzed reduction of **6** with benzothiazoline **3d** proceeded smoothly, providing rac-**1l** in 90% yield, suggesting that the excellent chemoselectivity should not derive from the chiral elements on the CPA catalyst (Scheme 5c). Similarly, the chemoselectivity of reduction with a Hantzsch ester at the C≡C bond over the C=N bond in Scheme 1B should also not originate from the chiral environment on CPA, as demonstrated by diphenyl phosphate catalyzed reduction of **6** with Hantzsch ester **8** affording rac-**5** in 35% yield (Scheme 5d).

**Fig. 1:**
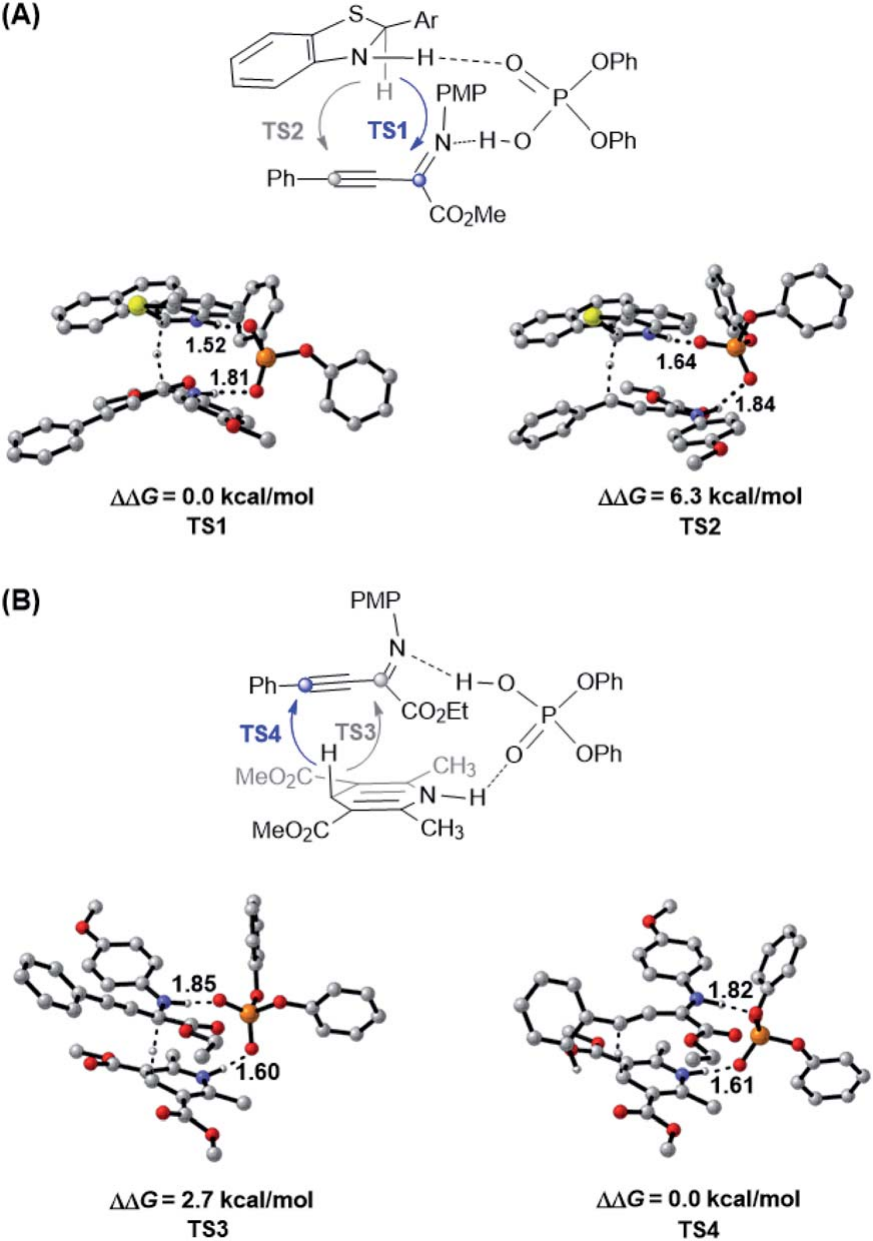
Optimized structures and the relative Gibbs free energies of chemoselectivity-determining transition states at the PCM-M06/6-311+G(d,p)//B3LYP/6-31G(d,p) level in toluene. (A) **TS1** and **TS2** for benzothiazoline-mediated transfer hydrogenation. (B) **TS3** and **TS4** for Hantzsch ester-mediated transfer hydrogenation. Key hydrogen-bonding interactions are marked with bond lengths in Å. Noncritical hydrogen atoms are omitted for clarity.

Due to the different hydride transfer modes (1,4 *vs.* 1,2) and hydrogen transfer abilities, benzothiazolines have exhibited superior reactivity and enantioselectivity to widely used Hantzsch esters in several CPA catalyzed transfer hydrogenation reactions.^17,18^ However, to our knowledge, different chemoselectivities have never been observed for these two types of hydride donors for the same substrate. To elucidate the origin of different chemoselectivities for benzothiazoline and the Hantzsch ester, density functional theory (DFT) calculations have been performed on the transition structures for respective transfer hydrogenation of the C=N bond (**TS1** and **TS3**) and C≡C bond (**TS2** and **TS4**) at the M06/6-311+G(d,p)//B3LYP/6-31G(d,p) level with the polarizable continuum model (PCM) (Fig. 1, see the ESI† for details).^22,23^ For the reduction with benzothiazoline, **TS1** is 6.3 kcal mol^−1^ more favorable than **TS2** in terms of free energy. In the case of the Hantzsch ester as the hydride donor, **TS3** for C=N bond reduction is 2.7 kcal mol^−1^ higher in energy than **TS4** for the C≡C bond. The results are consistent with the experimentally observed chemoselectivities.

To further understand this intriguing chemoselectivity, we conducted distortion/interaction analysis for the aforementioned four transition state structures at the PCM-M06/6-311+G(d,p)//B3LYP/6-31G(d,p) level in toluene (Table 2).^24^ We compared the energies of distortion of the CPA catalyst (Δ*E*^cat^_dist_), hydride donor (Δ*E*^hyd^_dist_), and substrate (Δ*E*^sub^_dist_) components, as well as the interaction energies among these three parts. Despite the small difference in catalyst and substrate distortion, drastically different hydride donor distortion energies were found, favoring reduction of the C≡C bond for both reductants. On the other hand, an obvious interaction energy difference was observed, favoring reduction of the C=N bond for both reductants. In the benzothiazoline-mediated reaction, the interaction energy is responsible for the observed selectivity of C=N bond reduction, as evidenced by the stronger hydrogen bonding interaction in **TS1** than in **TS2** (1.52 Å *vs.* 1.64 Å and 1.81 Å *vs.* 1.84 Å). In the Hantzsch ester-involved reaction, the distortion energy is responsible for the observed selectivity of C≡C bond reduction, probably due to the reacting hydride lying in a distal position with reference to the hydrogen-binding N–H moiety.

**Table 2:** Distortion/interaction analysis for the chemoselectivity-determining transition states^a^

TS	Δ*E*^‡^	Δ*E*^cat^_dist_	Δ*E*^hyd^_dist_	Δ*E*^sub^_dist_	Δ*E*_dist_^b^	Δ*E*_int_^c^
**TS1**	9.1	2.1	25.7	15.1	42.9	−33.8
**TS2**	15.4	1.9	14.6	18.9	35.4	−20.0
**TS3**	11.8	1.5	21.4	14.6	37.5	−25.7
**TS4**	9.1	1.0	10.0	16.7	27.7	−18.6

a Calculated at the PCM-M06/6-311+G(d,p)//B3LYP/6-31G(d,p) level in toluene. All energies are given in kcal mol^−1^.

b Δ*E*_dist_ = Δ*E*^cat^_dist_ + Δ*E*^hyd^_dist_ + Δ*E*^sub^_dist_.

c Δ*E*_int_ = Δ*E*^‡^ − Δ*E*_dist_.

**Fig. 2:**
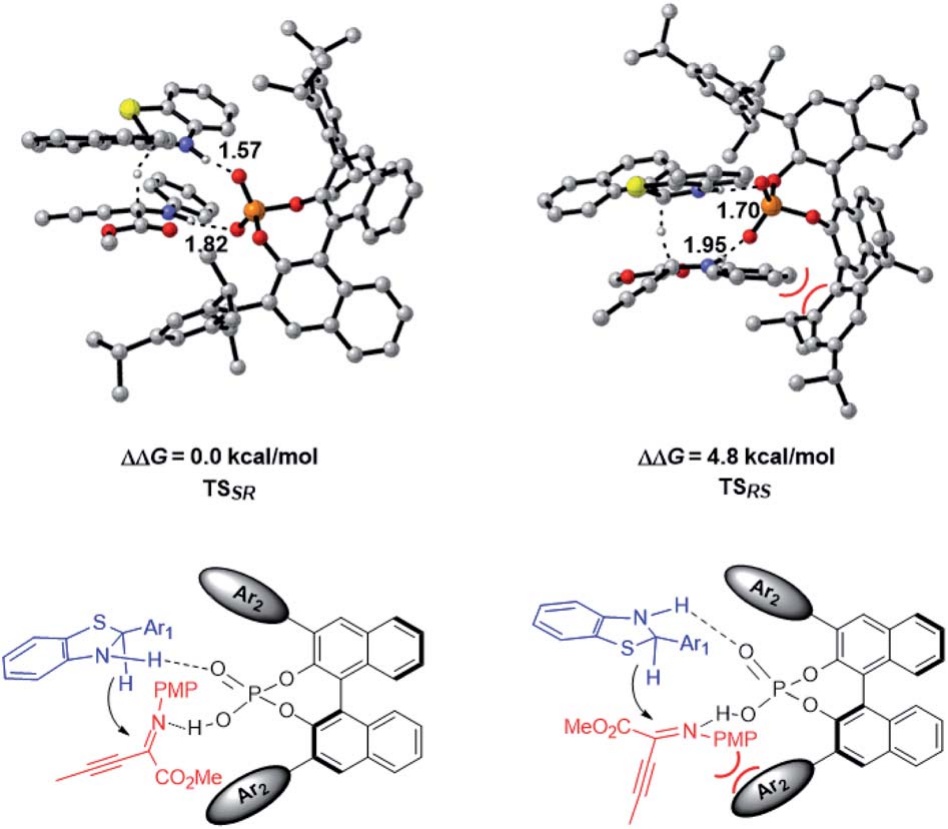
Lower-lying transition states and their relative Gibbs free energies for CPA-catalyzed transfer hydrogenation of β,γ-alkynyl-α-imino ester at the PCM-M06/6-311+G(d,p)//B3LYP/6-31G(d,p) level in *n*-decane. Key hydrogen-bonding interactions are marked with bond lengths in Å.

The enantioselective transfer hydrogenation of β,γ-alkynyl-α-imino ester with CPA **2a** and benzothiazoline **3d** was next investigated by DFT calculations at the PCM-M06/6-311+G(d,p)//B3LYP/6-31G(d,p) level in *n*-decane (Fig. 2). After exploring the possible transition structures for the process (see the ESI† for details), the most stable diastereomeric **TSSR** giving the major enantiomer and **TSRS** giving the minor enantiomer were compared. **TSSR** is 4.8 kcal mol^−1^ more favorable than **TSRS** in terms of free energy, which is in agreement with the experimentally observed enantioselectivity. A closer inspection of the two transition structures reveals that **TSRS** suffers from steric repulsion between the PMP group of the α-imino ester and the bulky triisopropylphenyl moiety of CPA when the *si*-face of the substrate is attacked by **3d**, while such steric repulsion could be avoided when the attack occurs from the *re*-face. The hydrogen bonding interactions of CPA with the respective substrate and **3d** are the second factor that differentiates the diastereomeric transition states. There are stronger NH⋯O interactions in **TSSR** than in **TSRS**, evidenced by shorter NH⋯O hydrogen bonding distances in **TSSR** than in **TSRS** (1.57 and 1.82 Å in **TSSR***vs.* 1.70 and 1.95 Å in **TSRS**). Therefore, our calculations suggest that the steric effect and hydrogen bonding interactions differentiate the enantiomeric reduction process.

## Conclusions

In summary, the first non-enzymatic redox deracemization method using molecular oxygen as the terminal oxidant has been revealed. The one-pot deracemization of β,γ-alkynyl α-amino acid derivatives consisted of a copper-catalyzed aerobic oxidative process and CPA-catalyzed asymmetric transfer hydrogenation with excellent functional group compatibility. By using benzothiazoline as the reductant, an exclusive chemoselectivity at the C=N bond over the C≡C bond was achieved, allowing for efficient deracemization of a series of α-amino esters bearing diverse α-alkynyl substituent patterns. The generality of the strategy is further demonstrated by efficient deracemization of β,γ-alkenyl α-amino esters. Mechanistic exploration by combined experiments and computations elucidated the origins of chemo- and enantio-selectivities. We envision that the sustainable method outlined herein will have potential applications in increasingly significant proteomics and peptide based drug discovery research.

## Conflicts of interest

There are no conflicts to declare.

## Supplementary Material

SC-011-D0SC00944J-s001
