# Supplementary material for: Redox deracemization of β,γ-alkynyl α-amino esters
Source: Chem Sci. 2020 Apr 21;11(17):4444–9. doi: 10.1039/d0sc00944j (PMC8159540; doi:10.1039/d0sc00944j)
Supplement: SC-011-D0SC00944J-s001 [file SC-011-D0SC00944J-s001.pdf]

Electronic Supplementary Material (ESI) for Chemical Science.
This journal is © The Royal Society of Chemistry 2020

Supplementary Information

Redox Deracemization of Non-Natural *β,γ*-Alkynyl α-Amino Esters

Lu Zhang, Rongxiu Zhu, Aili Feng, Changyin Zhao, Lei Chen, Guidong Feng and Lei Liu*

Table of contents

Table of contents S1

General information S2

Substrate preparation..S3

General procedure S4

Analytical data for products S5

Computation studies S21

References..S49

NMR spectra...S51

HPLC spectra for ee determination S78

General information

All reactions were carried out with dry solvents under anhydrous conditions. Standard syringe techniques were applied for transfer of dry solvents and some air-sensitive reagents and were introduced into reaction vessels through a rubber septum. Proton (^{1}H NMR) and carbon (^{13}C NMR) nuclear magnetic resonance spectra were recorded at 500 MHz, 400 MHz and 126 MHz, 101 MHz, respectively. The chemical shifts are given in parts per million (ppm) on the delta (δ) scale. The solvent peak was used as a reference value, for ^{1}H NMR: $CDCl_3$ = 7.26 ppm, for ^{13}C NMR: $CDCl_3$ = 77.23. Analytical TLC was performed on precoated silica gel GF254 plates. Column chromatography was carried out on silica gel (200–300 mesh). HRMS were carried out on an Orbitrap analyzer. UV spectra were obtained with an Agilent 8453E UV-Visible spectroscopy system. Optical rotations were measured using a 2.5 mL cell with a 10 cm path length on Hanon P850 Automatic Polarimeter and concentrations (c) were reported in g×(100 mL)$^{-1}$. Enantiomeric excesses were determined by HPLC using a Daicel Chiralpak AD-H, IG or IB column with hexane/*i*-PrOH as the eluent.

Substrate preparation

Synthesis of racemic *β,γ*-alkynyl α-amino esters[1]

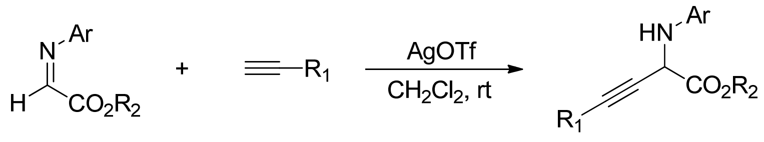

S1 **S2** rac-**1** or rac-**4**

Procedure A: A mixture of **S1** (0.5 mmol, 1.0 equiv), **S2** (1.0 mmol, 2.0 equiv), and AgOTf (0.05 mmol, 10 mol %) in CH_2Cl_2 (5 mL) was stirred overnight at rt for 12 h. The solvent was removed under vacumm, and the residue was purified by flash chromatography to give the desired product rac-**1** or rac-**4**.

Synthesis of racemic *β,γ*-alkenyl α-amino ester 5[2]

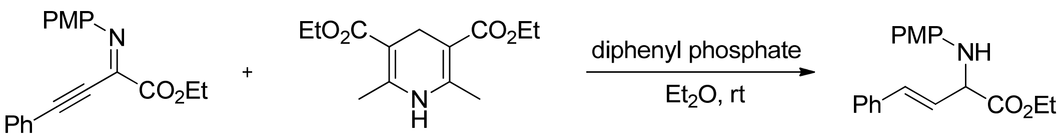

rac-**1l** **8** rac-**5**

Procedure B: A mixture of rac-**1l** (0.5 mmol, 1.0 equiv), **8** (1.1 mmol, 2.2 equiv) and diphenyl phosphate (0.05 mmol, 10 mol %) in Et_2O (10 mL) was stirred at rt under N_2 atmosphere. After all the rac-**1l** disappeared monitored by TLC, the solvent was removed under reduced pressure, and the residue was purified by silica gel column chromatography to afford the product rac-**5**.

General procedure

Procedure for redox deracemization of *β,γ*-alkynyl α-amino esters

A mixture of rac-**1** (0.1 mmol, 1.0 eq) and $Cu(OAc)_2$ (0.01 mmol, 10 mol %) in CH_2Cl_2 (2 mL) was stirred at rt under dioxygen atmosphere (dioxygen balloon, 1atm) for 3 hours. After all the rac-**1** disappeared monitored by TLC, the solvent was removed in vacuo, and decane (1 mL), mesitylene (1 mL), **2a** (0.01 mmol, 5 mol %), 3 Å MS (40 mg), and **3d** (0.12 mmol, 1.2 eq) were successively added. The mixture was stirred at rt for 4 hours under N_2 atmosphere, and then directly purified by flash chromatography to give the desired product.

Analytical data for products

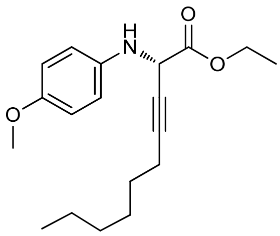

***(S)*-Ethyl 2-((4-methoxyphenyl)amino)dec-3-ynoate (1a)**

The compound was prepared followed by the general procedure, and purified by flash column chromatography using 5% solution of EtOAc in petroleum ether (28.4 mg, 86% yield). ^{1}H NMR (500 MHz, $CDCl_3$) δ 6.81 – 6.75 (m, 2H), 6.70 – 6.65 (m, 2H), 4.70 (t, *J* = 2.2 Hz, 1H), 4.26 (q, *J* = 7.1 Hz, 2H), 3.74 (s, 3H), 2.17 (td, *J* = 7.1, 2.2 Hz, 2H), 1.50 – 1.42 (m, 2H), 1.34 – 1.22 (m, 9H), 0.87 (t, *J* = 7.0 Hz, 3H); ^{13}C NMR (126 MHz, $CDCl_3$) δ 169.69, 153.38, 139.78, 116.09, 114.85, 85.57, 75.24, 62.30, 55.79, 50.30, 31.46, 28.57, 28.48, 22.71, 18.87, 14.24, 14.22; HRMS (ESI) m/z calculated for $C_{19}H_{28}NO_3$ $[M+H]^+$ 318.2064, found 318.2074; HPLC: the ee value was determined by HPLC analysis (Chiralcel AD-H, *i*-PrOH/Hexane = 25/75, 1.0 mL/min, 306 nm), retention time: t_{major} = 7.097 min, t_{minor} = 10.650 min, ee = 97%; $[\alpha]_D^{25}$ = +34.98 (c = 1.24, $CHCl_3$).

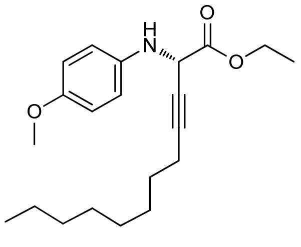

***(S)*-Ethyl 2-((4-methoxyphenyl)amino)dodec-3-ynoate (1b)**

The compound was prepared followed by the general procedure, and purified by flash column chromatography using 5% solution of EtOAc in petroleum ether (31.6 mg, 88% yield). ^{1}H NMR (500 MHz, $CDCl_3$) δ 6.81 – 6.76 (m, 2H), 6.70 – 6.64 (m, 2H), 4.70 (t, *J* = 2.2 Hz, 1H), 4.26 (q, *J* = 7.1 Hz, 2H), 3.74 (s, 3H), 2.17 (td, *J* = 7.1, 2.1 Hz, 2H), 1.49 – 1.42 (m, 2H), 1.32 – 1.23 (m, 14H), 0.88 (t, *J* = 7.0 Hz, 3H); ^{13}C NMR

(126 MHz, $CDCl_3$) δ 169.73, 153.43, 139.88, 116.11, 114.92, 85.60, 75.31, 62.30, 55.84, 50.38, 32.03, 29.38, 29.26, 28.94, 28.57, 22.85, 18.90, 14.29, 14.27; HRMS (ESI) m/z calculated for $C_{21}H_{32}NO_3$ $[M+H]^+$ 346.2377, found 346.2390; HPLC: the ee value was determined by HPLC analysis (Chiralcel AD-H,*i*-PrOH/Hexane = 25/75, 1.0 mL/min, 242 nm), retention time: t_{major}= 5.983 min, t_{minor}= 8.457 min, ee = 98%; $[\alpha]_D^{25}$ = +32.51 (c = 1.18, $CHCl_3$).

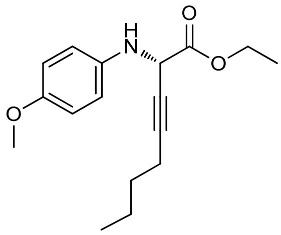

***(S)*-Ethyl 2-((4-methoxyphenyl)amino)oct-3-ynoate (1c)**

The compound was prepared followed by the general procedure, and purified by flash column chromatography using 5% solution of EtOAc in petroleum ether (24.5 mg, 81% yield). ^{1}H NMR (500 MHz, $CDCl_3$) δ 6.80 – 6.77 (m, 2H), 6.70 – 6.65 (m, 2H), 4.70 (t, *J* = 2.2 Hz, 1H), 4.26 (qd, *J* = 7.1, 1.0 Hz, 2H), 3.75 (s, 3H), 2.18 (td, *J* = 7.0, 2.2 Hz, 2H), 1.48 – 1.42 (m, 2H), 1.38 – 1.33 (m, 2H), 1.30 (t, *J* = 7.1 Hz, 3H), 0.87 (t, *J* = 7.3 Hz, 3H); ^{13}C NMR (126 MHz, $CDCl_3$) δ 169.73, 153.45, 139.82, 116.17, 114.91, 85.59, 75.26, 62.32, 55.86, 50.40, 30.60, 21.99, 18.58, 14.26, 13.74; HRMS (ESI) m/z calculated for $C_{17}H_{24}NO_3$ $[M+H]^+$ 290.1751, found 290.1758; HPLC: the ee value was determined by HPLC analysis (Chiralcel AD-H,*i*-PrOH/Hexane = 25/75, 1.0 mL/min, 241 nm), retention time: t_{major} =7.703min, t_{minor} = 11.873 min, ee = 94%; $[\alpha]_D^{25}$ = +55.86 (c = 1.32, $CHCl_3$).

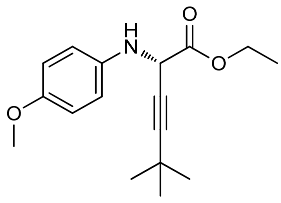

***(S)*-Ethyl 2-((4-methoxyphenyl)amino)-5,5-dimethylhex-3-ynoate (1d)**

The compound was prepared followed by the general procedure, and purified by flash

column chromatography using 5% solution of EtOAc in petroleum ether (25.7 mg, 85% yield). ^{1}H NMR (500 MHz, $CDCl_3$) δ 6.80 – 6.76 (m, 2H), 6.70 – 6.65 (m, 2H), 4.69 (s, 1H), 4.30 – 4.21 (m, 2H), 3.74 (s, 3H), 1.28 (t, *J* = 7.1 Hz, 3H), 1.18 (s, 9H); ^{13}C NMR (126 MHz, $CDCl_3$) δ 169.84, 153.48, 139.86, 116.29, 114.86, 93.77, 73.73, 62.14, 55.84, 50.53, 30.90, 27.58, 14.22; HRMS (ESI) m/z calculated for $C_{17}H_{24}NO_3[M+H]^+$ 290.1751, found 290.1762; HPLC: the ee value was determined by HPLC analysis (Chiralcel AD-H,*i*-PrOH/Hexane = 25/75, 1.0 mL/min, 242 nm), retention time: t_{major} = 4.953 min, t_{minor} = 6.690 min, ee = 96%; $[\alpha]_D^{25}$ = +47.82 (c = 1.12, $CHCl_3$).

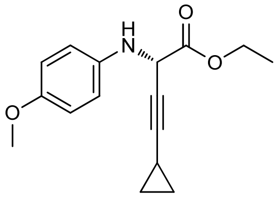

***(S)*-Ehyl 4-cyclopropyl-2-((4-methoxyphenyl)amino)but-3-ynoate (1e)**

The compound was prepared followed by the general procedure, and purified by flash column chromatography using 5% solution of EtOAc in petroleum ether (24.1 mg, 84% yield). ^{1}H NMR (500 MHz, $CDCl_3$) δ 6.80 – 6.76 (m, 2H), 6.67 – 6.63 (m, 2H), 4.66 (d, *J* = 1.8 Hz, 1H), 4.25 (q, *J* = 7.1 Hz, 2H), 3.74 (s, 3H), 1.29 (t, *J* = 7.1 Hz, 3H), 0.77 – 0.71 (m, 2H), 0.68 – 0.62 (m, 2H); ^{13}C NMR (126 MHz, $CDCl_3$) δ 169.62, 153.41, 139.82, 116.08, 114.89, 88.50, 70.37, 62.31, 55.83, 50.39, 14.24, 8.43, 8.42, -0.34; HRMS (ESI) m/z calculated for $C_{16}H_{20}NO_3$ $[M+H]^+$274.1438, found 274.1445; HPLC: the ee value was determined by HPLC analysis (Chiralcel AD-H,*i*-PrOH/Hexane = 25/75, 1.0 mL/min, 305 nm), retention time: t_{major} = 9.653 min,t_{minor} = 12.567min, ee = 98%; $[\alpha]_D^{25}$ = +63.9 (c = 0.68, $CHCl_3$).

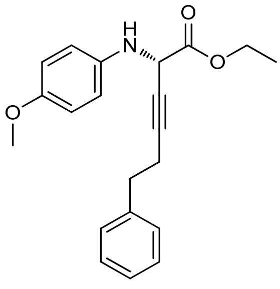

***(S)*-Ethyl 2-((4-methoxyphenyl)amino)-6-phenylhex-3-ynoate (1f)**

The compound was prepared followed by the general procedure, and purified by flash column chromatography using 5% solution of EtOAc in petroleum ether (28.9 mg, 86% yield). ^{1}H NMR (500 MHz, $CDCl_3$) δ 7.30 – 7.16 (m, 5H), 6.83 – 6.77 (m, 2H), 6.69 – 6.64 (m, 2H), 4.69 (t, *J* = 2.1 Hz, 1H), 4.30 – 4.23 (m, 2H), 3.76 (s, 3H), 2.79 (t, *J* = 7.4 Hz, 2H), 2.48 (td, *J* = 7.4, 2.1 Hz, 2H), 1.30 (t, *J* = 7.1 Hz, 3H);^{13}C NMR (126 MHz, $CDCl_3$) δ 169.52, 153.41, 140.59, 139.76, 128.65, 128.52, 126.47, 116.05, 114.92, 84.58, 76.14, 62.36, 55.84, 50.25, 34.89, 21.10, 14.25; HRMS (ESI) m/z calculated for $C_{20}H_{22}NO_3$ $[M+H]^+$ 324.1594, found 324.1607; HPLC: the ee value was determined by HPLC analysis (Chiralcel AD-H,*i*-PrOH/Hexane = 25/75, 1.0 mL/min, 240 nm), retention time: t_{major} =10.893 min, t_{minor}= 14.397 min, ee = 96%; $[\alpha]_D^{25}$ = +38.7 (c = 1.02, $CHCl_3$).

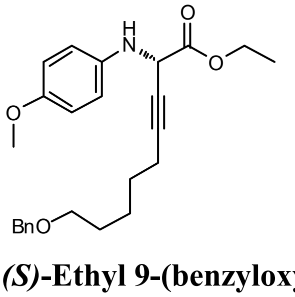

***(S)*-Ethyl 9-(benzyloxy)-2-((4-methoxyphenyl)amino)non-3-ynoate (1g)**

The compound was prepared followed by the general procedure, and purified by flash column chromatography using 5% solution of EtOAc in petroleum ether (34.7 mg, 85% yield). ^{1}H NMR (500 MHz, $CDCl_3$) δ 7.37 – 7.26 (m, 5H), 6.80 – 6.76 (m, 2H), 6.69 – 6.63 (m, 2H), 4.70 (t, *J* = 2.1 Hz, 1H), 4.49 (s, 2H), 4.26 (q, *J* = 7.1 Hz, 2H), 3.74 (s, 3H), 3.44 (t, *J* = 6.6 Hz, 2H), 2.19 (td, *J* = 6.9, 2.1 Hz, 2H), 1.63 – 1.56 (m, 2H), 1.53 – 1.47 (m, 2H), 1.45 – 1.39 (m, 2H), 1.29 (t, *J* = 7.1 Hz, 3H);^{13}C NMR (126 MHz, $CDCl_3$) δ 169.69, 153.43, 139.85, 138.84, 128.56, 127.81, 127.70, 116.12, 114.92,

85.33, 75.47, 73.10, 70.44, 62.31, 55.84, 50.36, 29.44, 28.39, 25.57, 18.87, 14.27; HRMS (ESI) m/z calculated for $C_{25}H_{32}NO_4$ $[M+H]^+$ 410.2326, found 410.2319; HPLC: the ee value was determined by HPLC analysis (Chiralcel AD-H, *i*-PrOH/Hexane = 25/75, 1.0 mL/min, 241 nm), retention time: t_{major} = 13.077 min, t_{minor} = 18.763 min, ee = 94%; $[\alpha]_D^{25}$ = +12.41 (c = 0.2, $CHCl_3$).

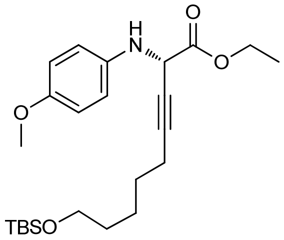

***(S)*-Ethyl**

9-((tert-butyldimethylsilyl)oxy)-2-((4-methoxyphenyl)amino)non-3-ynoate (1h)

The compound was prepared followed by the general procedure, and purified by flash column chromatography using 15% solution of EtOAc in petroleum ether (37.6 mg, 87% yield). ^{1}H NMR (500 MHz, $CDCl_3$) δ 6.79 – 6.75 (m, 2H), 6.68 – 6.64 (m, 2H), 4.69 (t, *J* = 2.1 Hz, 1H), 4.28 – 4.23 (m, 2H), 3.73 (s, 3H), 3.57 (t, *J* = 6.5 Hz, 2H), 2.17 (td, *J* = 7.0, 2.1 Hz, 2H), 1.52 – 1.45 (m, 4H), 1.40 – 1.33 (m, 2H), 1.28 (t, *J* = 7.1 Hz, 3H), 0.89 (s, 9H), 0.04 (s, 6H); ^{13}C NMR (126 MHz, $CDCl_3$) δ 169.60, 153.32, 139.77, 115.99, 114.80, 85.27, 75.34, 63.15, 62.19, 55.70, 50.24, 32.43, 28.33, 26.10, 25.14, 18.82, 18.47, 14.20, -5.15; HRMS (ESI) m/z calculated for $C_{24}H_{40}NO_4Si$ $[M+H]^+$ 434.2721, found 434.2728; HPLC: the ee value was determined by HPLC analysis (Chiralcel AD-H,*i*-PrOH/Hexane = 25/75, 1.0 mL/min, 243 nm), retention time: t_{major} =4.343min, t_{minor} = 5.117min, ee = 96%; $[\alpha]_D^{25}$=+36.3(c=1.6,$CHCl_3$).

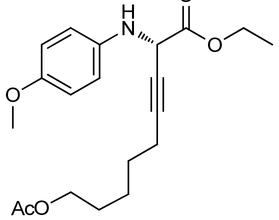

***(S)*-Ethyl9-acetoxy-2-((4-methoxyphenyl)amino)non-3-ynoate(1i)**

The compound was prepared followed by the general procedure, and purified by flash

column chromatography using 5% solution of EtOAc in petroleum ether (30.3 mg, 84% yield). ^{1}HNMR (500 MHz, $CDCl_3$) δ 6.80–6.76 (m, 2H), 6.70–6.65(m, 2H), 4.70 (t, *J* = 2.2 Hz, 1H), 4.26(q, *J* = 7.1 Hz, 2H), 4.02 (t, *J* = 6.7 Hz, 2H), 3.74 (s, 3H), 2.20 (dt, *J* = 7.0, 3.5 Hz, 2H), 2.04 (s, 3H), 1.62 – 1.56 (m, 2H), 1.52 – 1.48 (m, 2H), 1.41 – 1.36 (m, 2H), 1.30 (t, *J* = 7.1 Hz, 3H); ^{13}C NMR (126 MHz, $CDCl_3$) δ 171.38, 169.62, 153.50, 139.72, 116.19, 114.92, 85.09, 75.63, 64.53, 62.36, 55.85, 50.37, 28.28, 28.12, 25.23, 21.19, 18.80, 14.28; HRMS (ESI) m/z calculatedfor$C_{20}H_{28}NO_5$ $[M+H]^+$ 362.1962, found 362.1967; HPLC: the ee value was determined by HPLC analysis (Chiralcel IB,*i*-PrOH/Hexane = 25/75, 1.0 mL/min, 309 nm), retention time: t_{major} = 7.663min, t_{minor} = 9.057min, ee = 92%; $[\alpha]_D^{25}$ = +31.81 (c = 0.82, $CHCl_3$).

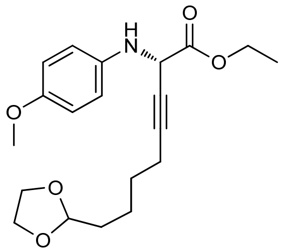

***(S)*-Ethyl 8-(1,3-dioxolan-2-yl)-2-((4-methoxyphenyl)amino)oct-3-ynoate (1j)**

The compound was prepared followed by the general procedure, and purified by flash column chromatography using 20% solution of EtOAc in petroleum ether (29.2 mg, 81% yield). ^{1}H NMR (500 MHz, $CDCl_3$) δ 6.80 – 6.76 (m, 2H), 6.69 – 6.65 (m, 2H), 4.81 (t, *J* = 4.8 Hz, 1H), 4.69 (s, 1H), 4.26 (q, *J* = 7.1 Hz, 2H), 4.15 (s, 1H), 3.97 – 3.91 (m, 2H), 3.86 – 3.81 (m, 2H), 3.74 (s, 3H), 2.19 (td, *J* = 6.9, 2.1 Hz, 2H), 1.66 – 1.61 (m, 2H), 1.55 – 1.44 (m, 4H), 1.30 (t, *J* = 7.1 Hz, 3H); ^{13}C NMR (126 MHz, $CDCl_3$) δ 169.69, 153.43, 139.87, 116.12, 114.93, 104.64, 85.18, 75.56, 65.05, 62.33, 55.85, 50.37, 33.52, 28.45, 23.43, 18.86, 14.27; HRMS (ESI) m/z calculated for $C_{20}H_{28}NO_5$ $[M+H]^+$ 362.1962, found 362.1975; HPLC: the ee value was determined by HPLC analysis (Chiralcel IB, *i*-PrOH/Hexane = 25/75, 1.0 mL/min, 309 nm), retention time: t_{major} = 8.320 min, t_{minor}= 9.643 min, ee = 95%; $[\alpha]_D^{25}$ = +49.76 (c = 1.6, $CHCl_3$).

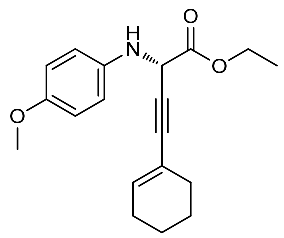

(S)-Ethyl 4-(cyclohex-1-en-1-yl)-2-((4-methoxyphenyl)amino)but-3-ynoate (1k)

The compound was prepared followed by the general procedure, and purified by flash column chromatography using 5% solution of EtOAc in petroleum ether (25.1 mg, 80% yield). ^{1}H NMR (500 MHz, $CDCl_3$) δ 6.81 – 6.77 (m, 2H), 6.70 – 6.66 (m, 2H), 6.10 (dd, J = 3.9, 2.0 Hz, 1H), 4.82 (s, 1H), 4.27 (q, J = 7.1 Hz, 2H), 3.75 (s, 3H), 2.09 – 2.03 (m, 4H), 1.61 – 1.54 (m, 4H), 1.30 (t, J = 7.1 Hz, 3H);^{13}C NMR (126 MHz, $CDCl_3$) δ 169.48, 153.45, 139.81, 136.34, 120.01, 116.15, 114.94, 86.41, 81.52, 62.41, 55.87, 50.77, 29.10, 25.80, 22.37, 21.59, 14.28; HRMS (ESI) m/z calculated for $C_{19}H_{24}NO_3$ $[M+H]^+$ 314.1751, found 314.1759; HPLC: the ee value was determined by HPLC analysis (Chiralcel AD-H,*i*-PrOH/Hexane = 25/75, 1.0 mL/min, 239 nm), retention time: t_{major} =7.947 min, t_{minor}= 10.163 min, ee = 95%; $[\alpha]_D^{25}$ = +119.88 (c = 1.04, $CHCl_3$).

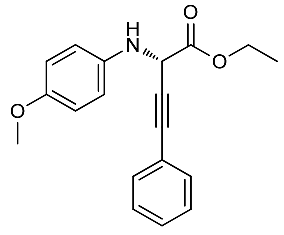

(S)-Ethyl 2-((4-methoxyphenyl)amino)-4-phenylbut-3-ynoate (1l)

The compound was prepared followed by the general procedure, and purified by flash column chromatography using 15% solution of EtOAc in petroleum ether (27.2 mg, 88% yield). ^{1}H NMR (400 MHz, $CDCl_3$) δ 7.40 – 7.34 (m, 2H), 7.28 – 7.21 (m, 3H), 6.82 – 6.75 (m, 2H), 6.73 – 6.69 (m, 2H), 4.92 (s, 1H), 4.27 (q, J = 7.1 Hz, 2H), 3.71 (s, 3H), 1.29 (t, J = 7.1 Hz, 3H);^{13}C NMR (101 MHz, $CDCl_3$) δ 169.15, 153.55, 139.61, 132.08, 128.84, 128.42, 122.32, 116.24, 114.96, 84.52, 84.36, 62.59, 55.82, 50.81, 14.27; HRMS (ESI) m/z calculated for $C_{19}H_{20}NO_3$ $[M+H]^+$ 310.1438, found 310.1451; HPLC: the ee value was determined by HPLC analysis (Chiralcel

AD-H,*i*-PrOH/Hexane = 25/75, 1.0 mL/min, 221 nm), retention time: t_{major} = 11.243 min, t_{minor}= 13.707 min, ee = 90%; $[\alpha]_D^{25}$ =+105.2 (c = 1.1, $CHCl_3$).

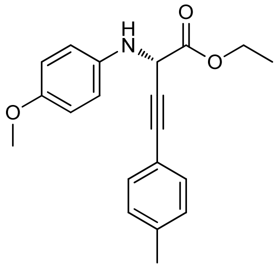

***(S)*-Ethyl 2-((4-methoxyphenyl)amino)-4-(p-tolyl)but-3-ynoate (1m)**

The compound was prepared followed by the general procedure, and purified by flash column chromatography using 10% solution of EtOAc in petroleum ether (29.4 mg, 91% yield). ^1H NMR (500 MHz, $CDCl_3$) δ 7.32 (dd, *J* = 8.1, 2.6 Hz, 2H), 7.10 (d, *J* = 8.2 Hz, 2H), 6.85 – 6.80 (m, 2H), 6.78 – 6.73 (m, 2H), 4.97 (d, *J* = 4.6 Hz, 1H), 4.32 (q, *J* = 7.1 Hz, 2H), 3.75 (s, 3H), 2.34 (s, 3H), 1.33 (t, *J* = 7.1 Hz, 3H); ^{13}C NMR (126 MHz, $CDCl_3$) δ 169.18, 153.44, 139.64, 139.62, 138.92, 131.89, 129.11, 129.10, 119.18, 116.12, 114.87, 84.60, 83.63, 83.59, 62.44, 55.72, 50.76, 21.58, 14.19; HRMS (ESI) m/z calculated for $C_{20}H_{22}NO_3$ $[M+H]^+$ 324.1594, found 324.1618; HPLC: the ee value was determined by HPLC analysis (Chiralcel AD-H,*i*-PrOH/Hexane = 25/75, 1.0 mL/min, 246 nm), retention time: t_{major} = 7.047 min, t_{minor}= 8.553 min, ee = 84%; $[\alpha]_D^{25}$ = +131.8 (c = 1.12, $CHCl_3$).

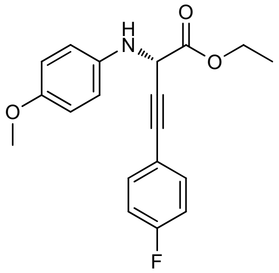

***(S)*-Ethyl 4-(4-fluorophenyl)-2-((4-methoxyphenyl)amino)but-3-ynoate (1n)**

The compound was prepared followed by the general procedure, and purified by flash column chromatography using 15% solution of EtOAc in petroleum ether (27.1 mg, 83% yield). ^1H NMR (500 MHz, $CDCl_3$) δ 7.41 – 7.35 (m, 2H), 7.01 – 6.96 (m, 2H),

6.83 – 6.79 (m, 2H), 6.76 – 6.71 (m, 2H), 4.93 (s, 1H), 4.31 (q, J = 7.1 Hz, 2H), 3.75 (s, 3H), 1.33 (t, J = 7.1 Hz, 3H); ^{13}C NMR (126 MHz, $CDCl_3$) δ 169.09, 162.91 (d, J = 250.0 Hz), 153.62, 139.61, 134.08, 134.02, 118.43 (d, J = 3.5 Hz), 116.25, 115.85, 115.67, 115.01, 84.16, 83.49, 62.64, 55.86, 50.80, 14.30; HRMS (ESI) m/z calculated for $C_{19}H_{19}FNO_3$ $[M+H]^+$ 328.1343, found 328.1359; HPLC: the ee value was determined by HPLC analysis (Chiralcel AD-H,*i*-PrOH/Hexane = 25/75, 1.0 mL/min, 311 nm), retention time: t_{major} = 12.347 min,t_{minor}= 14.740 min, ee = 88%; $[\alpha]_D^{25}$ = +92.96 (c = 1.04, $CHCl_3$).

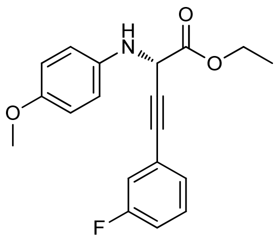

***(S)*-Ethyl 4-(3-fluorophenyl)-2-((4-methoxyphenyl)amino)but-3-ynoate (1o)**

The compound was prepared followed by the general procedure, and purified by flash column chromatography using 10% solution of EtOAc in petroleum ether (26.1 mg, 80% yield). ^{1}H NMR (500 MHz, $CDCl_3$) δ 7.26 – 7.22 (m, 1H), 7.18 (dt, J = 7.7, 1.1 Hz, 1H), 7.10 (ddd, J = 9.3, 2.5, 1.4 Hz, 1H), 7.02 (tdd, J = 8.5, 2.6, 1.0 Hz, 1H), 6.84 – 6.80 (m, 2H), 6.76 – 6.72 (m, 2H), 4.95 (s, 1H), 4.38 – 4.26 (m, 3H), 3.76 (s, 3H), 1.33 (t, J = 7.1 Hz, 3H);^{13}C NMR (126 MHz, $CDCl_3$) δ 168.90, 162.42 (d, J = 246.6 Hz), 153.64, 139.50, 130.05 (d, J = 8.6 Hz), 127.98, 124.15 (d, J = 9.5 Hz), 118.90 (d, J = 22.9 Hz), 116.25 (d, J = 21.2 Hz), 116.25, 115.00, 85.43, 83.25, 83.22, 62.70, 55.83, 50.75, 14.27; HRMS (ESI) m/z calculated for $C_{19}H_{19}FNO_3$ $[M+H]^+$ 328.1343, found 328.1349; HPLC: the ee value was determined by HPLC analysis (Chiralcel AD-H,*i*-PrOH/Hexane = 25/75, 1.0 mL/min, 244 nm), retention time: t_{major} =11.067 min, t_{minor}= 12.700 min, ee = 84%; $[\alpha]_D^{25}$ = +81.72 (c = 1.02, $CHCl_3$).

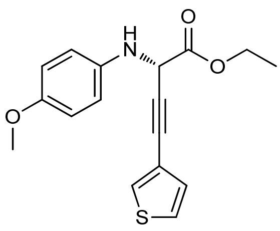

***(S)*-Ethyl 2-((4-methoxyphenyl)amino)-4-(thiophen-3-yl)but-3-ynoate (1p)**

The compound was prepared followed by the general procedure, and purified by flash column chromatography using 10% solution of EtOAc in petroleum ether (26.1 mg, 83% yield). ^{1}H NMR (500 MHz, $CDCl_3$) δ 7.44 (dd, *J* = 3.0, 1.1 Hz, 1H), 7.23 (dd, *J* = 5.0, 3.0 Hz, 1H), 7.07 (dd, *J* = 5.0, 1.1 Hz, 1H), 6.83 – 6.79 (m, 2H), 6.75 – 6.71 (m, 2H), 4.93 (s, 1H), 4.33 – 4.29 (m, 2H), 3.75 (s, 3H), 1.32 (t, *J* = 7.1 Hz, 3H);^{13}C NMR (126 MHz, $CDCl_3$) δ 169.13, 153.58, 139.65, 130.15, 129.80, 125.48, 121.38, 116.22, 115.00, 84.03, 79.75, 62.61, 55.86, 50.85, 14.28; HRMS (ESI) m/z calculated for $C_{17}H_{18}NO_3S$ $[M+H]^+$ 316.1002, found 316.9987; HPLC: the ee value was determined by HPLC analysis (Chiralcel AD-H,*i*-PrOH/Hexane = 25/75, 1.0 mL/min, 244 nm), retention time: t_{major} = 15.323 min, t_{minor}= 21.250 min, ee = 78%; $[\alpha]_D^{25}$ = +132.7 (c = 0.5, $CHCl_3$).

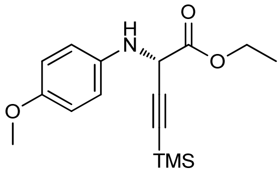

***(S)*-Ethyl 2-((4-methoxyphenyl)amino)-4-(trimethylsilyl)but-3-ynoate (1q)**

The compound was prepared followed by the general procedure, and purified by flash column chromatography using 3% solution of EtOAc in petroleum ether (24.7 mg, 81% yield). ^{1}H NMR (500 MHz, $CDCl_3$) δ 6.79 (d, *J* = 8.7 Hz, 2H), 6.67 (d, *J* = 8.8 Hz, 2H), 4.72 (s, 1H), 4.31 – 4.23 (m, 2H), 4.20 (s, 1H), 3.75 (s, 3H), 1.30 (t, *J* = 7.1 Hz, 3H), 0.14 (s, 9H);^{13}C NMR (126 MHz, $CDCl_3$) δ 169.07, 153.56, 139.71, 116.25, 114.91, 100.18, 89.99, 62.46, 55.86, 51.16, 14.20, -0.10; HRMS (ESI) m/z calculated for $C_{16}H_{24}NO_3Si$ $[M+H]^+$ 306.1520, found 306.1504; HPLC: the ee value was determined by HPLC analysis (Chiralcel AD-H, *i*-PrOH/Hexane = 25/75, 1.0 mL/min,

244 nm), retention time: t_{major} = 4.703min, t_{minor}= 5.723 min, ee = 92%; $[\alpha]_D^{25}$ = +111.8 (c = 1.16, $CHCl_3$).

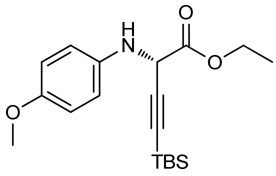

(S)-Ethyl4-(tert-butyldimethylsilyl)-2-((4-methoxyphenyl)amino)but-3-ynoate (1r)

The compound was prepared followed by the general procedure, and purified by flash column chromatography using 10% solution of EtOAc in petroleum ether (26.0 mg, 75% yield). ^{1}H NMR (500 MHz, $CDCl_3$) δ 6.80 – 6.76 (m, 2H), 6.71 – 6.66 (m, 2H), 4.74 (s, 1H), 4.32 – 4.23 (m, 2H), 3.74 (s, 3H), 1.30 (t, *J* = 7.1 Hz, 3H), 0.88 (s, 9H), 0.07 (s, 6H);^{13}C NMR (126 MHz, $CDCl_3$) δ 169.00, 153.52, 139.67, 116.27, 114.89, 100.84, 88.21, 62.41, 55.83, 51.04, 26.75, 26.11, 16.72, 14.21, -4.69, -4.70, -5.03; HRMS (ESI) m/z calculated for $C_{19}H_{30}NO_3Si$ $[M+H]^+$ 348.1989, found 348.1998;HPLC: the ee value was determined by HPLC analysis (Chiralcel AD-H,*i*-PrOH/Hexane = 10/90, 1.0 mL/min, 308 nm), retention time: t_{major} = 5.043 min, t_{minor}= 6.740min, ee = 93%; $[\alpha]_D^{25}$ = +93.76 (c = 0.56, $CHCl_3$).

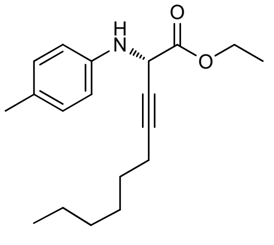

(S)-Ethyl 2-(p-tolylamino)dec-3-ynoate (4a)

The compound was prepared followed by the general procedure, and purified by flash column chromatography using 3% solution of EtOAc in petroleum ether (25.0 mg, 83% yield). ^{1}H NMR (500 MHz, $CDCl_3$) δ 7.01 (d, *J* = 8.0 Hz, 2H), 6.65 – 6.59 (m, 2H), 4.75 (d, *J* = 6.9 Hz, 1H), 4.27 (q, *J* = 7.1 Hz, 2H), 2.25 (s, 3H), 2.18 (td, *J* = 7.1, 2.2 Hz, 2H), 1.50 – 1.44 (m, 2H), 1.35 – 1.23 (m, 9H), 0.88 (t, *J* = 7.0 Hz, 3H); ^{13}C NMR

(126 MHz, $CDCl_3$) δ 169.67, 143.53, 129.87, 129.76, 128.41, 114.53, 85.46, 75.24, 62.30, 49.60, 31.48, 28.58, 28.51, 22.71, 20.63, 18.88, 14.25, 14.21; HRMS (ESI) m/z calculated for $C_{19}H_{28}NO_2$ $[M+H]^+$ 302.2115, found 302.2106; HPLC: the ee value was determined by HPLC analysis (Chiralcel IG, *i*-PrOH/Hexane = 15/85, 1.0 mL/min, 298 nm), retention time: t_{major} = 6.773 min, t_{minor}= 7.407 min, ee = 89%; $[\alpha]_D^{25}$ = +32.71 (c = 1.11, $CHCl_3$).

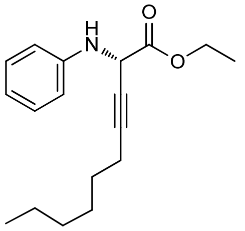

***(S)*-Ethyl 2-(phenylamino)dec-3-ynoate (4b)**

The compound was prepared followed by the general procedure, and purified by flash column chromatography using 2% solution of EtOAc in petroleum ether (22.4 mg, 78% yield). ^{1}H NMR (400 MHz, $CDCl_3$) δ 7.23 – 7.16 (m, 2H), 6.79 (t, *J* = 7.4 Hz, 1H), 6.73 – 6.66 (m, 2H), 4.78 (t, *J* = 2.2 Hz, 1H), 4.28 (q, *J* = 7.1 Hz, 2H), 2.18 (td, *J* = 7.0, 2.1 Hz, 2H), 1.52 – 1.44 (m, 2H), 1.35 – 1.25 (m, 9H), 0.88 (t, *J* = 6.9 Hz, 3H); ^{13}C NMR (101 MHz, $CDCl_3$) δ 169.54, 145.86, 129.37, 119.12, 114.30, 85.54, 75.11, 62.38, 49.25, 31.47, 28.58, 28.50, 22.71, 18.88, 14.24, 14.20; HRMS (ESI) m/z calculated for $C_{18}H_{26}NO_2$ $[M+H]^+$ 288.1958, found 288.1982; HPLC: the ee value was determined by HPLC analysis (Chiralcel AD-H,*i*-PrOH/Hexane = 20/80, 1.0 mL/min, 288 nm), retention time: t_{major} =6.510 min, t_{minor}= 9.467 min, ee = 90%; $[\alpha]_D^{25}$ = +20.13 (c = 1.02, $CHCl_3$).

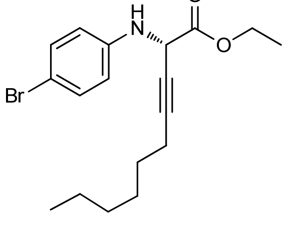

***(S)*-Ethyl 2-((4-bromophenyl)amino)dec-3-ynoate (4c)**

The compound was prepared followed by the general procedure, and purified by flash

column chromatography using 3% solution of EtOAc in petroleum ether (29.6 mg, 81% yield). ^{1}H NMR (500 MHz, $CDCl_3$) δ 7.30 – 7.26 (m, 2H), 6.59 – 6.55 (m, 2H), 4.72 (t, *J* = 2.2 Hz, 1H), 4.29 (q, *J* = 7.1 Hz, 2H), 2.17 (td, *J* = 7.1, 2.2 Hz, 2H), 1.49 – 1.42 (m, 2H), 1.33 – 1.23 (m, 9H), 0.87 (t, *J* = 7.0 Hz, 3H); ^{13}C NMR (126 MHz, $CDCl_3$) δ 169.17, 144.77, 132.14, 115.91, 110.97, 85.89, 74.59, 62.61, 49.03, 31.47, 28.59, 28.47, 22.74, 18.86, 14.27, 14.24; HRMS (ESI) m/z calculated for $C_{18}H_{25}BrNO_2$ $[M+H]^+$ 366.1063, found 366.1078; HPLC: the ee value was determined by HPLC analysis (Chiralcel AD-H,*i*-PrOH/Hexane = 25/75, 1.0 mL/min, 250 nm), retention time: t_{major} = 6.557min, t_{minor}= 9.843min, ee = 89%; $[\alpha]_D^{25}$ = +30.21 (c = 1.15, $CHCl_3$).

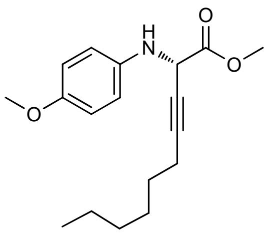

***(S)*-Methyl 2-((4-methoxyphenyl)amino)dec-3-ynoate (4d)**

The compound was prepared followed by the general procedure, and purified by flash column chromatography using 5% solution of EtOAc in petroleum ether (25.4 mg, 84 yield). ^{1}H NMR (500 MHz, $CDCl_3$) δ 6.81 – 6.76 (m, 2H), 6.69 – 6.64 (m, 2H), 4.73 (t, *J* = 2.2 Hz, 1H), 3.81 (s, 3H), 3.74 (s, 3H), 2.17 (td, *J* = 7.1, 2.2 Hz, 2H), 1.49 – 1.43 (m, 2H), 1.33 – 1.23 (m, 6H), 0.87 (t, *J* = 7.0 Hz, 3H); ^{13}C NMR (126 MHz, $CDCl_3$) δ 170.22, 153.46, 139.76, 116.10, 114.91, 85.77, 75.12, 55.81, 53.21, 50.20, 31.45, 28.59, 28.48, 22.70, 18.88, 14.20; HRMS (ESI) m/z calculated for $C_{18}H_{26}NO_3$ $[M+H]^+$ 304.1907, found 304.1910; HPLC: the ee value was determined by HPLC analysis (Chiralcel AD-H, *i*-PrOH/Hexane = 25/75, 1.0 mL/min, 242 nm), retention time: t_{major} = 10.667 min, t_{minor}= 15.793 min, ee = 95%; $[\alpha]_D^{25}$ = +62.3 (c = 1.22, $CHCl_3$).

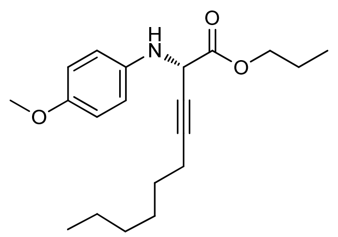

***(S)*-Propyl 2-((4-methoxyphenyl)amino)dec-3-ynoate (4e)**

The compound was prepared followed by the general procedure, and purified by flash column chromatography using 5% solution of EtOAc in petroleum ether (27.5 mg, 83% yield). ^{1}H NMR (500 MHz, $CDCl_3$) δ 6.81 – 6.75 (m, 2H), 6.70 – 6.64 (m, 2H), 4.71 (t, J = 2.2 Hz, 1H), 4.21 – 4.12 (m, 2H), 3.74 (s, 3H), 2.17 (td, J = 7.1, 2.2 Hz, 2H), 1.72 – 1.66 (m, 2H), 1.49 – 1.42 (m, 2H), 1.33 – 1.24 (m, 6H), 0.95 (t, J = 7.4 Hz, 3H), 0.87 (t, J = 7.0 Hz, 3H); ^{13}C NMR (126 MHz, $CDCl_3$) δ 169.85, 153.43, 139.86, 116.09, 114.93, 85.57, 75.33, 67.82, 55.86, 50.34, 31.50, 28.62, 28.53, 22.73, 22.13, 18.90, 14.24, 10.43; HRMS (ESI) m/z calculated for $C_{20}H_{30}NO_3$ $[M+H]^+$ 332.2220, found 332.2202 HPLC: the ee value was determined by HPLC analysis (Chiralcel AD-H, *i*-PrOH/Hexane = 25/75, 1.0 mL/min, 215 nm), retention time: t_{major} = 6.227 min, t_{minor}= 9.180 min, ee = 97%; $[\alpha]_D^{25}$ = +18.73 (c = 1.08, $CHCl_3$).

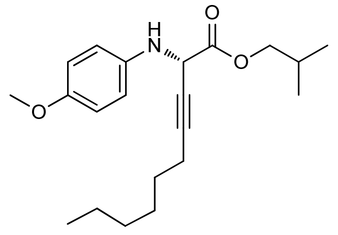

***(S)*-Isobutyl 2-((4-methoxyphenyl)amino)dec-3-ynoate (4f)**

The compound was prepared followed by the general procedure, and purified by flash column chromatography using 5% solution of EtOAc in petroleum ether (27.9 mg, 81% yield). ^{1}H NMR (500 MHz, $CDCl_3$) δ 6.80 – 6.75 (m, 2H), 6.69 – 6.65 (m, 2H), 4.72 (s, 1H), 4.19 (s, 1H), 4.04 (dd, J = 10.5, 6.7 Hz, 1H), 3.94 (dd, J = 10.5, 6.6 Hz, 1H), 3.73 (s, 3H), 2.17 (td, J = 7.1, 2.2 Hz, 2H), 2.03 – 1.95 (m, 1H), 1.49 – 1.43 (m, 2H), 1.34 – 1.22 (m, 6H), 0.94 (dd, J = 6.7, 1.2 Hz, 6H), 0.87 (t, J = 7.0 Hz, 3H); ^{13}C NMR (126 MHz, $CDCl_3$) δ 169.78, 153.35, 139.87, 115.97, 114.88, 85.43, 75.36, 72.09, 55.78, 50.24, 31.46, 28.59, 28.50, 27.96, 22.68, 19.06, 19.05, 18.84, 14.19; HRMS

(ESI) m/z calculated for $C_{21}H_{32}NO_3$ $[M+H]^+$ 346.2377, found 346.2369; HPLC: the ee value was determined by HPLC analysis (Chiralcel AD-H,*i*-PrOH/Hexane = 25/75, 1.0 mL/min, 242 nm), retention time: t_{major} = 6.617min, t_{minor}= 10.107 min, ee = 95%; $[\alpha]_D^{25}$ = +82.71 (c = 1.21, $CHCl_3$).

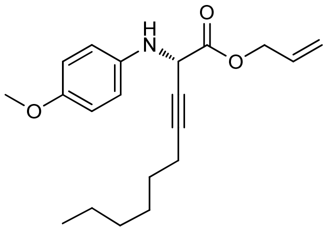

***(S)*-Allyl 2-((4-methoxyphenyl)amino)dec-3-ynoate (4g)**

The compound was prepared followed by the general procedure, and purified by flash column chromatography using 5% solution of EtOAc in petroleum ether (27.3 mg, 83% yield). ^{1}H NMR (500 MHz, $CDCl_3$) δ 6.81 – 6.76 (m, 2H), 6.70 (d, *J* = 8.8 Hz, 2H), 5.96 – 5.87 (m, 1H), 5.39 – 5.33 (m, 1H), 5.27 – 5.23 (m, 1H), 4.76 (t, *J* = 2.2 Hz, 1H), 4.70 (ddt, *J* = 10.3, 5.6, 1.4 Hz, 2H), 3.75 (s, 3H), 2.17 (td, *J* = 7.1, 2.2 Hz, 2H), 1.49 – 1.43 (m, 2H), 1.33 – 1.23 (m, 6H), 0.87 (t, *J* = 7.0 Hz, 3H); ^{13}C NMR (126 MHz, $CDCl_3$) δ 169.31, 153.68, 131.57, 118.94, 116.45, 114.93, 86.06, 74.94, 66.64, 55.85, 50.53, 31.49, 28.62, 28.49, 22.72, 18.90, 14.24; HRMS (ESI) m/z calculated for $C_{20}H_{28}NO_3$ $[M+H]^+$ 330.2064, found 330.2069; HPLC: the ee value was determined by HPLC analysis (Chiralcel AD-H, *i*-PrOH/Hexane = 25/75, 1.0 mL/min, 238 nm), retention time: t_{major} =6.813 min, t_{minor} = 10.407 min, ee = 92%; $[\alpha]_D^{25}$ = +83.7 (c = 0.24, $CHCl_3$).

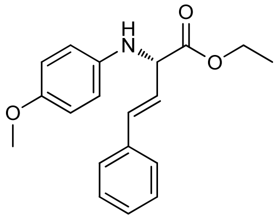

***(S,E)*-Ethyl 2-((4-methoxyphenyl)amino)-4-phenylbut-3-enoate (*(S)*-5)**

The compound was prepared followed by the general procedure, and purified by flash column chromatography using 5% solution of EtOAc in petroleum ether (25.5 mg, 82%

yield). ^{1}H NMR (500 MHz, $CDCl_3$) δ 7.39 – 7.36 (m, 2H), 7.31 (t, *J* = 7.5 Hz, 2H), 7.26 – 7.24 (m, 1H), 6.79 – 6.76 (m, 2H), 6.68 – 6.63 (m, 2H), 6.30 (dd, *J* = 15.9, 5.9 Hz, 1H), 4.65 (dd, *J* = 5.9, 1.5 Hz, 1H), 4.28 – 4.20 (m, 2H), 3.74 (s, 3H), 1.29 (t, *J* = 7.1 Hz, 3H); ^{13}C NMR (126 MHz, $CDCl_3$) δ 172.13, 152.91, 140.65, 136.44, 133.03, 128.79, 128.18, 126.85, 125.51, 115.33, 115.06, 61.97, 60.13, 55.93, 14.41; HRMS (ESI) m/z calculated for $C_{19}H_{22}NO_3$ $[M+H]^+$ 312.1594, found 312.1606; HPLC: the ee value was determined by HPLC analysis (Chiralcel AD-H,*i*-PrOH/Hexane = 25/75, 1.0 mL/min, 255 nm), retention time: t_{major} = 11.753 min, t_{minor}= 13.687 min, ee = 91%; $[\alpha]_D^{25}$ = +90.7 (c = 2.56, $CHCl_3$).

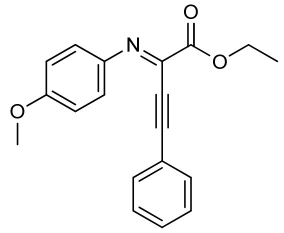

***(E)*-Ethyl 2-((4-methoxyphenyl)imino)-4-phenylbut-3-ynoate (6)**

^{1}H NMR (500 MHz, CDCl3) δ 7.59 – 7.53 (m, 2H), 7.50 – 7.45 (m, 2H), 7.43 – 7.39 (m, 1H), 7.38 – 7.33 (m, 2H), 6.98 – 6.94 (m, 2H), 4.47 (q, *J* = 7.1 Hz, 2H), 3.86 (s, 3H), 1.45 (t, *J* = 7.1 Hz, 3H); ^{13}C NMR (126 MHz, $CDCl_3$) δ 163.07, 159.66, 141.84, 139.31, 132.72, 130.35, 128.76, 125.02, 121.28, 113.96, 98.51, 83.57, 62.93, 55.70, 14.43; HRMS (ESI) m/z calculated for $C_{19}H_{18}NO_3$ $[M + H]^+$ 308.1281, found 308.1289.

Computation studies

Computation details

Density functional theory (DFT) calculations were carried out with Gaussian 09 package.[3] The B3LYP functional[4] with the standard 6-31G(d,p)[5] basis set was used for geometry optimizations in the gas phase. The vibrational frequencies were performed at the same level of theory to verify transition state structures (only one imaginary frequency) and to obtain thermal corrections. To get more accurate energy, single-point energy calculations of all the transition state structures were conducted at the M06/6-311+G(d,p) level[6] with polarizable continuum model (PCM)[7] in toluene for diphenyl phosphate catalysis or in n-decane for chiral phosphoric acid catalysis. The Gibbs free energy used in this article is the sum of the electronic energy in the solution phase and the gas-phase thermal correction. Noncovalent interactions (NCIs) analyses were performed by using the Multiwfn program,[8] and all the NCI isosurfaces were rendered by VMD 1.9.3 program.[9] The 3D diagrams of structures were prepared using CYL view.[10]

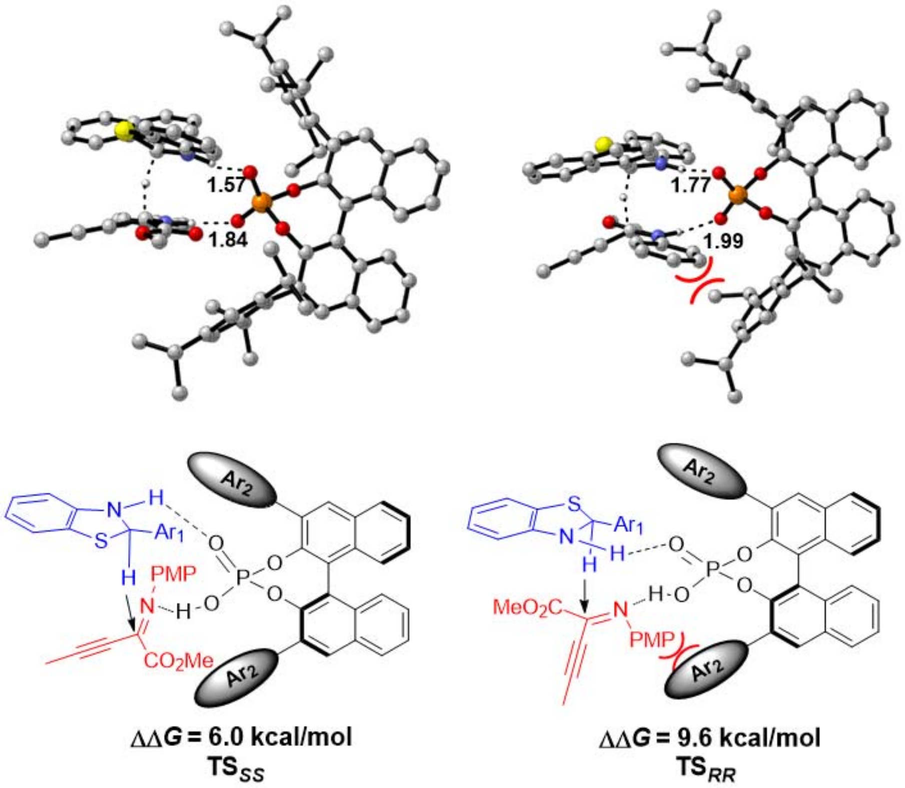

Fig. S1. Another two higher-lying transition states and their relative Gibbs free energies for CPA-catalyzed transfer hydrogenation of β,γ-alkynyl-α-imino ester at the PCM-M06/6-311+G(d, p)//B3LYP/6-31G(d, p) level in n-decane. Key hydrogen-bonding interactions are marked with bond lengths in Å.

Table S1. Electronic energies (*E*, in Hartree) of all the transition states at the PCM-M06/6-311+G(d, p)//B3LYP/6-31G(d, p) level and their thermal corrections to Gibbs free energy (G^{corr}, in hartree) at the B3LYP/6-31G(d, p) level.

TS	E	G^{corr}
**TS1**	-3228.34887	0.682122
**TS2**	-3228.34053	0.683892
**TS3**	-2903.728464	0.679281
**TS4**	-2903.734857	0.681289
$\mathbf{TS}_{\boldsymbol{RS}}$	-4357.960921	1.278551
$\mathbf{TS}_{\boldsymbol{RR}}$	-4357.954284	1.279097
$\mathbf{TS}_{\boldsymbol{SS}}$	-4357.958734	1.278286
$\mathbf{TS}_{\boldsymbol{SR}}$	-4357.968337	1.278366

Cartesian coordinates of the calculated species

TS1

0 1

C	-4.72382500	-2.34572100	0.75989900
C	-3.99974500	-2.30718000	-0.43626400
C	-3.66999800	-3.49128700	-1.10212800
C	-4.06546900	-4.71790500	-0.57065800
C	-4.78213900	-4.77091500	0.62814500
C	-5.10497400	-3.58145200	1.28583800
C	-6.74293200	1.69048000	-0.23357500
C	-5.48813000	1.40570300	-0.78205800
C	-5.25242700	1.59829100	-2.14726900
C	-6.29099700	2.06807400	-2.95401300
C	-7.54755200	2.35366700	-2.41837500
C	-7.76653600	2.16423400	-1.05173100
O	-3.61283200	-1.13486800	-1.04884100
O	-4.53489600	0.95726400	0.11275300
P	-3.10677300	0.22302700	-0.26756700
O	-2.51652800	-0.12843400	1.08272900
O	-2.32157400	1.02740100	-1.26477700

H	-5.66738600	-3.61004900	2.21522600
H	-6.10759100	2.21405400	-4.01520600
H	-0.52347000	0.87044700	-1.13239000
H	-6.89470900	1.53333800	0.82944800
H	-8.73891500	2.38208400	-0.61836000
H	-8.34644500	2.71943400	-3.05665900
H	-4.27121000	1.39548100	-2.55879200
H	-3.10133600	-3.42358000	-2.02323700
H	-3.81021400	-5.63505700	-1.09479100
H	-5.09203800	-5.72668700	1.04072500
H	-4.97848300	-1.42353200	1.26812600
C	-0.33048200	3.37644400	-0.94160700
C	0.68434500	2.46249600	-0.60206100
C	1.83503600	2.94437000	0.05335900
C	1.97355300	4.29958000	0.31991300
C	0.96980800	5.20859300	-0.04173500
C	-0.18994600	4.73340100	-0.66949600
O	1.21056600	6.51614800	0.26461000
C	0.22317400	7.47706600	-0.07592200
N	0.46464800	1.11339100	-0.91122100
C	1.38162500	0.08195000	-0.94423000
H	-1.23580400	3.00105400	-1.40583700
H	2.61890900	2.26586900	0.36060100
H	2.85461000	4.67640300	0.82859400
H	-0.99104200	5.40699200	-0.94947700
H	0.61580100	8.44147100	0.25017400
H	0.04389400	7.50794500	-1.15825600
H	-0.72687700	7.28188500	0.43789500
C	0.98542100	3.42656000	4.08852300
C	-0.30937700	3.37104900	3.54735900
C	-0.72381600	2.28955500	2.77803900
C	0.18803000	1.24677600	2.56124700
C	1.48322500	1.29743200	3.11371800
C	1.89510900	2.39092300	3.87472800
S	2.37807600	-0.16248500	2.71237500
N	-0.05411000	0.10628400	1.82985100
C	1.01722100	-0.74123200	1.64021200
C	0.77090500	-2.21968400	1.55003500
C	-0.50977700	-2.70311700	1.76842100
C	-0.79224900	-4.08542700	1.70362800
C	0.21299400	-4.98631200	1.44848200
C	1.54134400	-4.54119700	1.21120400
C	1.83462500	-3.13489300	1.23100300
C	2.58401400	-5.46788100	0.94132700

C	3.86777800	-5.04208700	0.68539800
C	4.15448600	-3.65831300	0.67138800
C	3.16571200	-2.73225000	0.93010600
H	1.28416600	4.28401600	4.68334900
H	-0.99986000	4.18870200	3.72898700
H	-1.71969900	2.22634300	2.35412400
H	2.89397000	2.43138900	4.29711600
H	-1.03379700	-0.05160400	1.41784000
H	1.42589600	-0.43546900	0.35402200
H	-1.32367200	-2.02089500	1.98361300
H	-1.81271400	-4.42010400	1.85937300
H	0.00674500	-6.05297200	1.41672500
H	2.34473600	-6.52817000	0.94150000
H	4.65616300	-5.76234900	0.48756200
H	5.16212500	-3.31755900	0.45147900
H	3.41819400	-1.67968100	0.87684300
C	0.79157800	-1.16600200	-1.59903900
O	-0.39726400	-1.39463400	-1.60710800
O	1.73551500	-1.96815400	-2.09890200
C	1.25650600	-3.21582800	-2.66972700
H	0.54338800	-2.97923400	-3.46474500
H	0.72241500	-3.76746100	-1.89097600
C	2.76715600	0.34704200	-1.20351000
C	3.95475300	0.51511900	-1.39999600
C	5.33943400	0.73082800	-1.65517800
C	5.86590400	2.03804500	-1.67825600
C	6.19867900	-0.36138200	-1.88998500
C	7.22008100	2.24140900	-1.92856700
H	5.20272100	2.87890400	-1.50239300
C	7.55133500	-0.14629500	-2.13824800
H	5.79002100	-1.36675200	-1.87801500
C	8.06586900	1.15291300	-2.15818900
H	7.61710600	3.25206500	-1.94596700
H	8.20640600	-0.99349400	-2.31895000
H	9.12153600	1.31654400	-2.35350600
C	2.46514700	-3.97165100	-3.18426700
H	2.99612700	-3.39042100	-3.94367600
H	3.15657800	-4.20030500	-2.36901700
H	2.14162200	-4.91415300	-3.63700900

TS2

0 1

C	-2.90681200	-3.74776400	0.64170100
C	-3.01834200	-3.42546900	-0.71426200

C	-2.75228800	-4.38576700	-1.69467300
C	-2.36713200	-5.67222600	-1.31623000
C	-2.24296200	-6.00384500	0.03543800
C	-2.51541500	-5.03716300	1.00714300
C	-5.58676100	-0.34118400	2.18566400
C	-5.06764800	-0.32571600	0.88675100
C	-5.91094200	-0.12164600	-0.21017300
C	-7.27815400	0.05709000	0.01203200
C	-7.80778600	0.04170600	1.30296600
C	-6.95273600	-0.15650500	2.38971100
O	-3.44449700	-2.18871000	-1.15187900
O	-3.70044200	-0.51348600	0.79165700
P	-2.83437300	-0.76099500	-0.59982100
O	-1.41654400	-0.96428300	-0.12425600
O	-3.17032500	0.28181200	-1.62858300
H	-2.43529900	-5.28853600	2.06137200
H	-7.93272400	0.21331900	-0.84117500
H	-1.61865800	0.96984400	-2.33125000
H	-4.90679000	-0.49901600	3.01681200
H	-7.34819700	-0.17081500	3.40170200
H	-8.87284600	0.18276300	1.46116600
H	-5.49977500	-0.08721100	-1.21153600
H	-2.85650800	-4.10452800	-2.73741500
H	-2.16630300	-6.41734700	-2.08124200
H	-1.94840600	-7.00726000	0.32868900
H	-3.12933200	-2.99810400	1.39225700
C	0.20325300	3.70801000	-1.64187400
C	-0.87157600	2.82330500	-1.80398700
C	-2.18996000	3.30896300	-1.66200400
C	-2.41389300	4.63972700	-1.36044400
C	-1.33557100	5.52624600	-1.19775200
C	-0.02640600	5.05069000	-1.34311100
O	-1.66580500	6.81378700	-0.90163700
C	-0.61900200	7.75756300	-0.73417900
N	-0.72680700	1.47587400	-2.16179000
C	0.35225700	0.67744200	-2.09803600
H	1.22198100	3.36260700	-1.75776000
H	-3.01674800	2.61419900	-1.76984400
H	-3.42218300	5.02236300	-1.24268800
H	0.82334600	5.71255400	-1.22738600
H	-1.10407000	8.70875700	-0.51052000
H	0.04190800	7.48451900	0.09847600
H	-0.02040400	7.86367600	-1.64794800
C	-0.40699400	3.41990700	3.83121200

C	-1.40621600	2.94653800	2.96756500
C	-1.17470800	1.87278600	2.11285600
C	0.09087200	1.27072900	2.13103300
C	1.08713900	1.73342900	3.01481300
C	0.85015500	2.81342500	3.86278400
S	2.53229100	0.72927600	2.90326200
N	0.48950300	0.19469700	1.36801000
C	1.84094600	-0.14769400	1.43910500
C	2.19051600	-1.60776800	1.29490200
C	1.16965100	-2.53986800	1.20482500
C	1.44325800	-3.92107800	1.08343900
C	2.74082900	-4.37201600	1.06835000
C	3.82420600	-3.45672800	1.15372900
C	3.55912300	-2.04806100	1.25458700
C	5.16823200	-3.91590000	1.13631100
C	6.22298700	-3.03501300	1.20739800
C	5.96920700	-1.64818000	1.29329300
C	4.67600300	-1.16999400	1.31507800
H	-0.61212100	4.26044700	4.48678200
H	-2.38091100	3.42440000	2.96190800
H	-1.94911900	1.49255600	1.45707100
H	1.62128300	3.16667600	4.54015600
H	-0.19356600	-0.26855200	0.72440100
H	2.38328400	0.43662500	0.42668000
H	0.13346700	-2.22500900	1.21975600
H	0.61078900	-4.61429700	1.01061900
H	2.95931600	-5.43398900	0.99031000
H	5.34695500	-4.98570500	1.06449800
H	7.24563900	-3.40033200	1.19379900
H	6.79816400	-0.94802300	1.33590100
H	4.52332300	-0.09915800	1.36553400
C	0.13737600	-0.66865000	-2.76263500
O	-0.82961500	-0.90373600	-3.45341600
O	1.13609600	-1.51766700	-2.50858300
C	0.98168400	-2.84877200	-3.06822300
H	0.68839900	-2.75382500	-4.11742300
H	0.16653100	-3.34513900	-2.53257200
C	1.56770900	0.96535500	-1.55142100
C	2.63435700	1.04522500	-0.88657200
C	4.02298200	1.47343000	-0.91875700
C	4.49739500	2.43646900	-0.00907200
C	4.90882400	0.92204400	-1.86321700
C	5.82924200	2.84619100	-0.05457300
H	3.81663900	2.85968400	0.72241600

C	6.23636400	1.33951300	-1.90304000
H	4.54123400	0.17185200	-2.55550900
C	6.70088300	2.30012900	-0.99950700
H	6.18588500	3.59425600	0.64736100
H	6.91154100	0.91373500	-2.63954900
H	7.73787600	2.62118200	-1.03263900
C	2.30320500	-3.57142300	-2.90024100
H	2.21770200	-4.58579100	-3.30280500
H	3.10326800	-3.05577100	-3.43989000
H	2.58041600	-3.64170100	-1.84557600

TS3

0 1

C	-5.78848100	-1.93070400	-0.10188400
C	-4.97435200	-1.56739700	-1.17953600
C	-5.33617000	-1.90883200	-2.48679000
C	-6.51599000	-2.61230800	-2.71893900
C	-7.34185800	-2.97698300	-1.65270300
C	-6.97008200	-2.63194300	-0.35283200
C	-5.50720100	3.11142200	0.19725200
C	-4.62827500	2.15315700	-0.31923500
C	-4.07286500	2.32047700	-1.59297700
C	-4.40446200	3.45630100	-2.33540500
C	-5.27882800	4.41821200	-1.82834600
C	-5.82965100	4.23740300	-0.55705200
O	-3.77845200	-0.88543000	-1.05511500
O	-4.38643300	1.06846300	0.49726600
P	-3.22721300	-0.08344000	0.27215500
O	-3.32556100	-0.95126100	1.51282600
O	-1.91628000	0.52775900	-0.12415900
H	-7.60329800	-2.91179200	0.48490100
H	-3.97302900	3.58222300	-3.32489500
H	-0.09240600	0.34370100	-0.39154900
H	-5.92579300	2.95183300	1.18553300
H	-6.51509100	4.97495300	-0.14846100
H	-5.53136200	5.29536300	-2.41673100
H	-3.39269200	1.57926300	-1.99311700
H	-4.68280800	-1.61275400	-3.30093600
H	-6.79080200	-2.87280600	-3.73744900
H	-8.26313500	-3.52273800	-1.83374000
H	-5.49154300	-1.67924900	0.90885400
C	0.08024200	2.86249300	-0.39902400
C	1.15769900	1.98420000	-0.19380800
C	2.40120000	2.51764200	0.19515000

C	2.55850700	3.89130000	0.33319200
C	1.48694300	4.76686900	0.10423400
C	0.24011300	4.23885500	-0.25235600
O	1.75640400	6.09526200	0.26534400
C	0.70437800	7.02190800	0.04376400
N	0.90893800	0.60673800	-0.37476100
C	1.80634900	-0.41080000	-0.60376200
H	3.23531400	1.86823100	0.41910400
H	3.51554300	4.30977300	0.62862000
H	-0.61535100	4.88193600	-0.42167800
H	1.13177500	8.01075300	0.21730100
H	0.32664900	6.96719500	-0.98522400
H	-0.12989200	6.86230300	0.73888600
H	-1.89761800	-1.28916300	2.13210200
N	-0.86025900	-1.44202100	2.30189100
C	-0.33635200	-2.64251900	1.91395000
C	-0.13534400	-0.35593100	2.69553000
C	1.03849300	-2.75135900	1.79341800
C	1.24610500	-0.41170200	2.58222900
C	1.84704700	-1.54413800	1.90112200
H	2.00486300	-1.06965500	0.64030000
H	2.91182600	-1.69434100	2.06896100
C	-0.95301200	0.79170000	3.21951100
H	-2.01578200	0.57518300	3.10976200
H	-0.72111500	0.97375100	4.27156900
H	-0.71688800	1.70646000	2.67250600
C	-1.36403600	-3.71083900	1.65968500
H	-2.34984200	-3.25491000	1.55086200
H	-1.12476400	-4.27158100	0.75695800
H	-1.38686800	-4.42388100	2.48982000
C	2.19447500	0.63898400	3.01844300
O	3.38227600	0.60931700	2.74249900
C	1.77659500	-3.98508900	1.45128700
O	2.94787300	-3.98974100	1.10979800
O	1.63749500	1.62111200	3.75569400
O	1.04793600	-5.11965400	1.57608800
C	1.75641400	-6.33846600	1.29897200
H	1.04716000	-7.14068000	1.50212000
H	2.08085300	-6.37142500	0.25578600
H	2.63458300	-6.42714400	1.94271300
C	2.53462600	2.66845400	4.16545300
H	2.89515500	3.21828100	3.29322800
H	1.94471200	3.32017800	4.80966700
H	3.38655500	2.25392200	4.70882100

H	-0.89061900	2.45344000	-0.65634800
C	3.14142300	-0.12486600	-1.03441100
C	4.28468900	0.05275300	-1.40382600
C	1.15279700	-1.64349600	-1.22636100
O	-0.03008200	-1.88456000	-1.12352700
O	2.05720300	-2.43494800	-1.80605500
C	1.57706000	-3.69972800	-2.32915400
H	1.01101300	-3.49944000	-3.24453800
H	0.89071800	-4.14236000	-1.60250300
C	5.61812600	0.29463100	-1.84021800
C	6.49170000	-0.78124100	-2.09511600
C	6.07885000	1.61454900	-2.01991000
C	7.79525200	-0.53706800	-2.51774100
H	6.13553700	-1.79635600	-1.95373500
C	7.38407300	1.84667000	-2.44391900
H	5.40389500	2.44177100	-1.82538900
C	8.24490100	0.77439200	-2.69336100
H	8.46308800	-1.37157500	-2.71010200
H	7.73138000	2.86653400	-2.58046200
H	9.26275100	0.96024100	-3.02341400
C	2.79467900	-4.56855200	-2.57704800
H	3.34047900	-4.72810100	-1.64308400
H	2.48213300	-5.53865800	-2.97667800
H	3.46844700	-4.10110300	-3.30071600

TS4

0 1

C	-5.18828900	-2.36479400	1.72644300
C	-5.24514900	-2.42114500	0.33052800
C	-6.35378900	-2.98058800	-0.31222400
C	-7.41216000	-3.48199500	0.44254900
C	-7.37282900	-3.42592400	1.83787800
C	-6.26018100	-2.86699100	2.46768600
C	-5.14603200	2.36821700	0.25579600
C	-4.63329100	1.23880200	-0.39498400
C	-4.92469400	1.01393600	-1.74603800
C	-5.72636100	1.93068900	-2.43104800
C	-6.23954200	3.05996800	-1.79173200
C	-5.94568100	3.27161000	-0.44148000
O	-4.23952000	-1.97039400	-0.50606800
O	-3.85498200	0.41120800	0.38061300
P	-3.02430600	-0.93528400	-0.12202000
O	-2.30700500	-1.38200600	1.13341400
O	-2.28818400	-0.67758100	-1.40399600

H	-6.21684500	-2.82162000	3.55267200
H	-5.95015500	1.75207500	-3.47929600
H	-0.55479200	-0.22425400	-1.74336400
H	-4.91135500	2.51155300	1.30554700
H	-6.34568400	4.13997700	0.07542700
H	-6.86485100	3.76220100	-2.33490700
H	-4.52077700	0.14609700	-2.25241600
H	-6.36593100	-3.01061700	-1.39675100
H	-8.27079100	-3.91488100	-0.06337200
H	-8.19902000	-3.81432200	2.42605500
H	-4.31565500	-1.94757700	2.21357200
C	-1.24271500	2.16915100	-1.51199400
C	0.05977900	1.69211600	-1.27377000
C	1.02096900	2.57960800	-0.74861100
C	0.67267300	3.89494500	-0.46988000
C	-0.63004600	4.36128200	-0.70242800
C	-1.58873100	3.48380200	-1.23060300
O	-0.86306400	5.66302700	-0.38625300
C	-2.16917700	6.18390300	-0.60203700
N	0.30581400	0.34573400	-1.59456000
C	1.47525000	-0.31652100	-1.66259500
H	2.03019600	2.25122400	-0.55230200
H	1.40524800	4.58533200	-0.06506600
H	-2.60622000	3.80368600	-1.41825400
H	-2.13563400	7.22234700	-0.26976200
H	-2.44432800	6.15162500	-1.66341500
H	-2.92233500	5.63884100	-0.02032700
H	-0.74959200	-1.16592700	1.49084400
N	0.28676900	-1.04386800	1.64240500
C	1.09222900	-2.09779200	1.31134000
C	0.72856600	0.16201000	2.12775600
C	2.46541600	-1.92785600	1.36344800
C	2.08648300	0.37968400	2.22823600
C	3.03797400	-0.61988000	1.71584600
C	-0.37704400	1.10406700	2.52908000
H	-0.20506500	2.10057500	2.12151300
H	-1.33977800	0.72630300	2.18062000
H	-0.40931900	1.20553100	3.61784600
C	0.34488800	-3.35823400	0.95853700
H	0.57376000	-3.67861500	-0.05935700
H	0.64090100	-4.17035500	1.62596500
H	-0.72965900	-3.18904300	1.03492500
C	2.72395800	1.60030200	2.76317900
O	3.91908100	1.81859600	2.65204100

C	3.47446400	-2.95765400	1.04921400
O	4.67448900	-2.72568500	1.04190800
O	1.88461500	2.45125500	3.39976700
O	2.98080200	-4.18886600	0.77399200
C	3.97130700	-5.20126800	0.53137700
H	4.58546900	-4.94383500	-0.33489700
H	4.62369500	-5.31696500	1.40029000
C	2.50328400	3.63972400	3.91724700
H	2.96828400	4.21559400	3.11325500
H	1.69858900	4.20980000	4.38153200
H	3.26949600	3.38537300	4.65375300
H	-1.98784500	1.48605400	-1.90059500
C	2.72013400	0.15636100	-1.34557900
C	3.80318500	0.32914500	-0.72868200
C	1.34919300	-1.72922800	-2.18549100
O	0.28180900	-2.28172000	-2.34553300
O	2.54536000	-2.26735900	-2.45639200
C	2.53271100	-3.62010800	-2.97812900
H	1.77765000	-3.68670400	-3.76602200
H	2.23586300	-4.29627300	-2.16989900
H	3.97792700	-0.66989000	2.26813100
H	3.51415000	-0.17121400	0.65254200
C	5.14692700	0.86713500	-0.71577900
C	5.41793100	2.07412700	-1.38874600
C	6.18647000	0.19147200	-0.05310100
C	6.71148400	2.58808300	-1.40264100
H	4.61148500	2.59165500	-1.89851100
C	7.47655700	0.71590600	-0.07481800
H	5.97149500	-0.74019700	0.46077800
C	7.74291300	1.91215300	-0.74537900
H	6.91443900	3.51818900	-1.92551200
H	8.27704800	0.18921000	0.43618600
H	8.75031700	2.31806600	-0.75479300
C	3.92967500	-3.91876700	-3.48673200
H	3.97303700	-4.94436500	-3.86618200
H	4.20268900	-3.23915800	-4.29869400
H	4.66912800	-3.81124600	-2.68795700
H	3.41182500	-6.11855400	0.34778600

TS*SR*

0 1

H	1.63913400	-0.34647300	-1.49617300
C	1.03651300	-1.45398200	-3.67954700
C	2.12806500	-1.81957900	-2.86446800

C	2.84852300	-2.98944700	-3.16510600
C	2.50382400	-3.74881000	-4.28219800
C	1.43979700	-3.37366400	-5.10246000
C	0.70697400	-2.22572800	-4.78755800
N	2.40829500	-0.99526400	-1.76341400
C	3.53116800	-0.99048100	-0.96698900
H	0.45433000	-0.57961300	-3.41320000
H	3.66648100	-3.30940700	-2.53448200
H	3.06951600	-4.64977700	-4.50207900
H	-0.13346000	-1.92782600	-5.40768700
C	0.09989500	-5.84810800	-1.79922000
C	-0.77095100	-4.74882800	-1.73541600
C	-0.45264700	-3.61461600	-0.99590400
C	0.77001900	-3.59657000	-0.31146500
C	1.63875100	-4.70491900	-0.36702900
C	1.31475800	-5.83623600	-1.11375700
S	3.06874800	-4.41835500	0.61585200
N	1.25666500	-2.55598000	0.45325000
C	2.54718600	-2.69475200	0.92776400
C	2.90813300	-2.10022300	2.26165600
C	1.91602400	-1.50828400	3.02723200
C	2.19692200	-0.98139600	4.30721600
C	3.46482800	-1.06246600	4.82977500
C	4.52094100	-1.63967300	4.07522700
C	4.25916400	-2.14571600	2.75641000
C	5.83850500	-1.70861300	4.60197400
C	6.87350800	-2.23474700	3.86386200
C	6.62910500	-2.69984000	2.55253600
C	5.36117200	-2.65283300	2.01292500
H	-0.17310800	-6.71901100	-2.38685200
H	-1.71312700	-4.77474300	-2.27196500
H	-1.12696200	-2.76824700	-0.93648800
H	1.98784700	-6.68646300	-1.15699800
H	0.65361200	-1.68806200	0.61955000
H	3.27678100	-1.95324800	0.05030600
H	0.90445400	-1.42880200	2.64921400
H	1.39244800	-0.52521700	4.87521900
H	3.68127100	-0.67987900	5.82377600
H	6.01341400	-1.33093200	5.60605700
H	7.87571800	-2.28248900	4.27922500
H	7.44991300	-3.09102900	1.95854400
H	5.22127800	-2.98430300	0.99076800
C	3.53094900	0.17769500	0.01138700
O	2.51603400	0.75154800	0.33412100

O	4.74725100	0.40657700	0.51721400
C	4.79097600	1.37194900	1.59175200
H	4.33241200	2.30928300	1.27584000
H	4.26005900	0.98018900	2.46202900
C	4.80362600	-1.41083600	-1.48149100
C	5.89817100	-1.75514900	-1.87137500
H	5.84900200	1.50199600	1.81594800
C	7.21155100	-2.14387600	-2.36795800
H	7.65366600	-1.34256400	-2.97054000
H	7.14815500	-3.03932500	-2.99597800
H	7.89768000	-2.35737500	-1.54113200
P	-0.75100300	0.43896000	-0.18338500
O	-0.42147500	-0.57736200	0.88975700
O	-0.01235100	0.42123700	-1.49121200
O	-0.69344200	1.91943500	0.55517400
O	-2.36316100	0.33121400	-0.57556900
C	-0.55214200	4.07860600	-0.52327700
C	-1.34019800	3.00329000	-0.01110600
C	-2.72863800	3.05138400	0.02795600
C	-1.22017100	5.16751800	-1.04804800
H	-0.64148200	6.01111700	-1.41353600
C	-2.63121600	5.21799700	-1.15498500
C	-3.40674900	4.13781800	-0.62741000
C	-4.81786300	4.18007100	-0.81017300
C	-5.42214000	5.24301100	-1.44469800
C	-4.65364700	6.32847300	-1.92804400
C	-3.28564500	6.31020700	-1.78673200
H	-5.42184200	3.35416200	-0.45458000
H	-6.50007100	5.24510100	-1.57968100
H	-5.14391100	7.16386100	-2.41956800
H	-2.67855900	7.12640800	-2.17034200
C	-3.33033300	0.65184900	0.35676800
C	-3.50109600	1.98217600	0.72022200
C	-5.16481000	-0.04253600	1.74453100
C	-4.18870400	-0.38690300	0.82995800
H	-5.85067300	-0.80903800	2.09434900
C	-5.29148700	1.26855000	2.26531500
C	-4.43998700	2.30214000	1.76098700
C	-4.54596800	3.59694400	2.34217700
C	-5.46094800	3.85679200	3.33861500
C	-6.32775600	2.84076200	3.80679500
C	-6.23937600	1.57327000	3.27976000
H	-3.88389700	4.38396000	2.00116200
H	-5.51443000	4.85120600	3.77272500

H	-7.05001500	3.06153900	4.58745700
H	-6.88556900	0.77719000	3.64114200
C	0.94837500	4.11826600	-0.48554600
C	1.68474000	4.03253500	-1.69220800
C	1.62137700	4.38425700	0.72787200
C	3.06634000	4.25268000	-1.66124700
C	1.02772600	3.71146000	-3.03560600
C	3.00546800	4.59703800	0.70242000
C	0.89024300	4.47305000	2.06608700
C	3.74937700	4.54745100	-0.47900500
H	3.62058000	4.19998000	-2.59411000
H	-0.01030700	3.43061800	-2.84180700
C	1.69164400	2.50579400	-3.72433100
C	1.01879500	4.93543200	-3.97247300
H	3.51912500	4.82667800	1.63399000
H	-0.17167600	4.29140700	1.88387300
C	1.37144300	3.38309900	3.04286000
C	1.00439300	5.87735800	2.68971900
C	5.24324200	4.85153300	-0.46835200
H	1.14534100	2.24529100	-4.63784200
H	2.72835400	2.71512400	-4.01147700
H	1.68036600	1.63885000	-3.06218700
H	0.52182600	4.69202800	-4.91834800
H	0.49258800	5.78433800	-3.52514400
H	2.03823000	5.26343400	-4.20629200
H	2.42109900	3.53323900	3.32289000
H	0.77806700	3.40756900	3.96413000
H	1.27406900	2.39195700	2.59300400
H	0.61731700	6.64503800	2.01180000
H	0.43293400	5.92974900	3.62329600
H	2.04402900	6.13296700	2.92312500
H	5.57000600	4.81467900	0.58017500
C	6.07804200	3.82017700	-1.24882700
C	5.51915300	6.27834200	-0.98391800
H	7.14790400	4.02567100	-1.13105000
H	5.88590600	2.79928500	-0.90442800
H	5.85683200	3.85400900	-2.32104400
H	4.97011000	7.02261000	-0.39883700
H	6.58741700	6.51698000	-0.92576300
H	5.20735000	6.38131700	-2.02927500
C	-4.11909600	-1.80681200	0.34983200
C	-3.78121700	-2.84260900	1.25489100
C	-4.51661000	-2.13543500	-0.96977100
C	-3.89642000	-4.17619700	0.83981700

C	-3.27940400	-2.56963800	2.67443700
C	-4.59614400	-3.48357100	-1.33551100
C	-4.91256400	-1.07819600	-2.00050800
C	-4.31600700	-4.52413400	-0.44450700
H	-3.64795300	-4.96048300	1.54795000
H	-3.18150400	-1.48838900	2.79523200
C	-1.87973500	-3.16921200	2.90429000
C	-4.27069100	-3.07127700	3.74249100
H	-4.91619900	-3.73497200	-2.34473500
H	-4.81319600	-0.09481500	-1.53603100
C	-3.97855000	-1.09394100	-3.22546000
C	-6.38825400	-1.22461800	-2.42186000
C	-4.53952400	-5.96953700	-0.87842100
H	-1.51570000	-2.90848600	3.90484700
H	-1.88325200	-4.26221200	2.82932100
H	-1.17447000	-2.77479200	2.17017600
H	-3.90467400	-2.83346200	4.74765100
H	-5.25719900	-2.61134700	3.62617100
H	-4.40401400	-4.15756600	3.68636700
H	-4.03265700	-2.04932800	-3.76029500
H	-4.26307800	-0.30428800	-3.92998300
H	-2.94198200	-0.92147100	-2.92483600
H	-7.05493400	-1.17803900	-1.55461600
H	-6.67022000	-0.42021200	-3.11044200
H	-6.57145800	-2.17657700	-2.93233100
H	-4.34508100	-6.01110900	-1.95970500
C	-3.60484200	-6.98464000	-0.20275400
C	-6.01500000	-6.36979100	-0.66666800
H	-3.74036600	-7.97662400	-0.64715000
H	-2.55394400	-6.70101500	-0.31171000
H	-3.81919000	-7.08064900	0.86728300
H	-6.69070200	-5.68670400	-1.19026900
H	-6.20195700	-7.38549300	-1.03382700
H	-6.27244600	-6.34142400	0.39808000
H	1.17885700	-3.97206600	-5.97024000

TS*RS*

0 1

H	-0.28676100	-2.33120500	-0.73876300
C	1.17893900	-3.22897400	1.11992100
C	-0.11808200	-3.69224700	0.82823200
C	-0.72640300	-4.62028600	1.69371400
C	-0.02969100	-5.10008300	2.80140900
C	1.26013400	-4.65270900	3.08176900

C	1.85260400	-3.70842600	2.23816500
N	-0.73782900	-3.16600000	-0.31707800
C	-1.93226100	-3.52367700	-0.89625700
H	1.64996300	-2.50322100	0.46628500
H	-1.73279000	-4.96503900	1.51148600
H	-0.51402600	-5.81864100	3.45622800
H	2.85191700	-3.34285300	2.43974800
C	-2.48761000	-2.98008800	5.12666900
C	-1.36391000	-2.20987400	4.78432100
C	-1.24196500	-1.62719800	3.52782800
C	-2.28280100	-1.82651300	2.60941200
C	-3.41687900	-2.58331300	2.95733700
C	-3.52457300	-3.17715000	4.21508400
S	-4.57625800	-2.59589400	1.63858000
N	-2.33476400	-1.31695400	1.32761500
C	-3.40046700	-1.72801700	0.53825400
C	-3.95248600	-0.79216500	-0.49982000
C	-3.37272300	0.45584600	-0.65194600
C	-3.83604400	1.36778800	-1.62500700
C	-4.89932000	1.03996200	-2.42924900
C	-5.52896000	-0.22796900	-2.31673200
C	-5.03972600	-1.18323300	-1.36177500
C	-6.62952300	-0.56774800	-3.14845100
C	-7.22810900	-1.80407300	-3.06897300
C	-6.72686900	-2.76226100	-2.16011100
C	-5.66141900	-2.46274600	-1.33736600
H	-2.55548100	-3.42516200	6.11461200
H	-0.57064100	-2.06970900	5.51181100
H	-0.38521500	-1.03030100	3.23678300
H	-4.39780100	-3.76394800	4.48147800
H	-1.54994300	-0.67641400	1.04617800
H	-2.89587800	-2.69777600	-0.23880500
H	-2.55120200	0.76676600	-0.01795600
H	-3.35257100	2.33308500	-1.70683300
H	-5.27234600	1.74465300	-3.16783500
H	-6.98981000	0.17542000	-3.85501100
H	-8.07202200	-2.04880300	-3.70718300
H	-7.17727100	-3.74947700	-2.11403900
H	-5.27882800	-3.24266200	-0.69028100
C	-2.06084400	-2.98537800	-2.32458300
O	-1.27368900	-2.21715300	-2.82139400
O	-3.15439400	-3.46736800	-2.92952200
C	-3.37922700	-2.97577300	-4.26507800
H	-2.58746000	-3.32347700	-4.93302300

H	-3.40044100	-1.88444200	-4.26968200
C	-2.52495100	-4.81480900	-0.69030800
C	-3.10753800	-5.87202100	-0.57652700
H	-4.34514100	-3.38158500	-4.56177100
C	-3.75849700	-7.16816800	-0.43963900
H	-4.82919400	-7.05271800	-0.23857300
H	-3.65170900	-7.75827100	-1.35656700
H	-3.32089700	-7.74272400	0.38407500
H	1.79575400	-5.02572800	3.94963700
P	0.68679900	0.40590500	0.01380300
O	-0.16407800	0.28386400	1.26170300
O	0.48585800	-0.57604000	-1.10022700
O	2.28193600	0.40885500	0.50975100
O	0.52733400	1.95381300	-0.59755800
C	4.23252200	0.03213400	-0.89599200
C	3.25485100	0.91310800	-0.33889900
C	3.30904900	2.28532200	-0.54111000
C	5.18972600	0.57751900	-1.72921300
H	5.95787700	-0.07263600	-2.13854400
C	5.20569000	1.94810000	-2.08283000
C	4.24658300	2.82513500	-1.48699300
C	4.25204600	4.19131700	-1.88650800
C	5.17031700	4.66103300	-2.79957600
C	6.13920000	3.79552900	-3.36097200
C	6.15124700	2.46588000	-3.00901500
H	3.51247800	4.86621000	-1.47240300
H	5.14754900	5.70615500	-3.09519200
H	6.86142900	4.18090000	-4.07493600
H	6.87867000	1.78473800	-3.44352400
C	1.02511600	3.02576800	0.12517900
C	2.40264000	3.18246200	0.22263800
C	0.66223500	5.04703900	1.38094700
C	0.12386600	3.99874900	0.65892400
H	-0.00547000	5.81580200	1.75887800
C	2.04721700	5.16584500	1.64606600
C	2.94177600	4.21811500	1.05978700
C	4.32820100	4.33259300	1.36167600
C	4.79692500	5.34017100	2.17551400
C	3.91013700	6.29554900	2.72721400
C	2.56255300	6.20560200	2.46654200
H	5.01938600	3.60532100	0.95280500
H	5.85824700	5.39995000	2.39949700
H	4.29468900	7.08904100	3.36152800
H	1.86658300	6.92316500	2.89398600

C	4.33745900	-1.42725500	-0.56465700
C	4.01669100	-2.40669900	-1.53668300
C	4.91004900	-1.81828100	0.66858400
C	4.26960200	-3.75267200	-1.24584300
C	3.45684700	-2.04352400	-2.91371200
C	5.14426500	-3.17892000	0.90388800
C	5.33350800	-0.80243000	1.73048300
C	4.83526600	-4.16399000	-0.03627000
H	4.02874000	-4.49988500	-1.99723400
H	3.22333600	-0.97608400	-2.91000200
C	2.14623400	-2.77936600	-3.24030100
C	4.50884400	-2.29016100	-4.01467000
H	5.59946700	-3.48116600	1.84500300
H	5.11678700	0.19685800	1.34577800
C	4.53438100	-0.95989500	3.03729800
C	6.85078700	-0.85894200	1.99473600
C	5.14183600	-5.63010200	0.24406600
H	1.79967400	-2.49662200	-4.24046300
H	2.27114600	-3.86838300	-3.23119900
H	1.36146000	-2.50141900	-2.53671700
H	4.12108200	-1.97963700	-4.99131100
H	5.43223400	-1.73343100	-3.82591100
H	4.77179400	-3.35215900	-4.08184900
H	4.71513900	-1.93495600	3.50542400
H	4.82760000	-0.18981700	3.75971400
H	3.46173400	-0.85765700	2.85124000
H	7.41902800	-0.70287000	1.07215900
H	7.14374000	-0.08193600	2.70972600
H	7.15364800	-1.82512600	2.41313400
H	5.50799600	-5.68822500	1.27806400
C	3.89036600	-6.52187400	0.14760600
C	6.26513200	-6.15426600	-0.67170100
H	4.13396400	-7.55671700	0.41403000
H	3.10103700	-6.17016300	0.81810000
H	3.48452000	-6.53028600	-0.86996400
H	7.17167500	-5.54896600	-0.57250200
H	6.51736900	-7.19168700	-0.42405600
H	5.96064500	-6.12678000	-1.72380700
C	-1.35586500	4.04985100	0.39917800
C	-2.27451000	3.75083100	1.43509800
C	-1.82203600	4.60266600	-0.81697500
C	-3.62888800	4.05091600	1.24532700
C	-1.82445900	3.14337300	2.76565100
C	-3.18987900	4.87543800	-0.95562000

C	-0.88204900	4.93625800	-1.97542800
C	-4.10888100	4.62827700	0.06634300
H	-4.32668600	3.83893800	2.05039500
H	-0.81805400	2.74070700	2.62169900
C	-2.71466700	1.96923000	3.20992800
C	-1.76526900	4.20410600	3.88364500
H	-3.54851800	5.31534300	-1.88372500
H	0.14257300	4.76671100	-1.63584200
C	-1.11565700	4.00017400	-3.17711400
C	-0.97377900	6.41637200	-2.39242500
C	-5.57440400	5.01369400	-0.09630200
H	-2.27731000	1.47900400	4.08580400
H	-3.72185900	2.29825600	3.48961200
H	-2.80948800	1.22060100	2.42089500
H	-1.44241700	3.74892300	4.82682500
H	-1.06558600	5.00922500	3.64456000
H	-2.75102600	4.65446100	4.04865800
H	-2.12930500	4.11177100	-3.58038300
H	-0.41097000	4.22933700	-3.98433200
H	-0.97035700	2.95510500	-2.88953300
H	-0.78040400	7.08024100	-1.54366200
H	-0.23586700	6.63772800	-3.17138900
H	-1.96117400	6.66808900	-2.79468400
H	-5.70963400	5.32427000	-1.14129400
C	-6.53502100	3.83961000	0.16437200
C	-5.93120600	6.22221700	0.79182200
H	-7.57071900	4.14062700	-0.02929900
H	-6.30087400	2.98352800	-0.47501300
H	-6.48119100	3.50171000	1.20489800
H	-5.28161800	7.07663700	0.57752300
H	-6.96967100	6.53262600	0.62946200
H	-5.81710300	5.97569700	1.85327600

TS*SS*

0 1

H	-1.72229400	-0.94963500	-1.14817700
C	-2.16714200	-0.11809600	-3.44281000
C	-3.17387400	-0.58634100	-2.57202200
C	-4.51257300	-0.56350200	-3.00079500
C	-4.82482700	-0.09973800	-4.27756300
C	-3.82815000	0.34792800	-5.14507000
C	-2.49948200	0.33855800	-4.71322200
N	-2.74248100	-1.06067500	-1.31835800
C	-3.46156100	-1.51007800	-0.23610200

H	-1.13859500	-0.11174700	-3.09920300
H	-5.30582500	-0.89282100	-2.34773200
H	-5.86507500	-0.08770800	-4.59056900
H	-1.70923000	0.69278400	-5.36890200
H	-3.67065700	-0.39367400	0.63632000
C	-2.57392400	-2.23363600	0.78040700
O	-1.36920200	-2.14672900	0.78039800
O	-3.30268800	-2.88981400	1.69161000
C	-2.54427600	-3.51293600	2.75230900
H	-1.74895900	-4.13225800	2.33521500
H	-2.10813100	-2.74571400	3.39608300
C	-4.79106700	-2.03965600	-0.35614600
C	-5.90090800	-2.52668200	-0.33824600
H	-3.26564400	-4.11394200	3.30491200
C	-7.23114700	-3.11977500	-0.35432100
H	-7.28863100	-3.92923700	-1.09058800
H	-7.99371600	-2.37692800	-0.61424200
H	-7.48524100	-3.53757600	0.62571100
P	0.86940600	0.04548400	-0.14460600
O	0.22114600	0.67553200	1.07197200
O	0.03768900	-0.42830800	-1.30009800
O	1.86173700	-1.13531000	0.46488400
O	1.97780600	1.09113700	-0.79721700
C	2.86817300	-3.00768500	-0.67382700
C	2.91348400	-1.63518200	-0.27790100
C	4.02016600	-0.82718300	-0.51699900
C	3.94188800	-3.50461500	-1.38537400
H	3.94283900	-4.55281800	-1.67107900
C	5.02957800	-2.69190200	-1.78800600
C	5.06904400	-1.32493500	-1.36736700
C	6.13674500	-0.51294600	-1.84376100
C	7.11945200	-1.03321000	-2.65700500
C	7.09792900	-2.39648200	-3.03546300
C	6.06977900	-3.20487200	-2.60963700
H	6.16508100	0.53520500	-1.57159700
H	7.91671000	-0.38819800	-3.01547900
H	7.88366100	-2.79567700	-3.67036800
H	6.02655800	-4.24934600	-2.90812000
C	3.10410200	1.45964700	-0.09014200
C	4.11721100	0.52761300	0.09776100
C	4.37708200	3.18859700	0.98737400
C	3.22240500	2.82090200	0.32559200
H	4.50529500	4.22515500	1.28682300
C	5.38831000	2.25609300	1.32561600

C	5.25499300	0.89657900	0.89811700
C	6.24821700	-0.03278000	1.31695300
C	7.32329300	0.36790500	2.07947800
C	7.47265800	1.72163300	2.46308900
C	6.51978700	2.64248500	2.09453500
H	6.14627600	-1.07475700	1.03900700
H	8.06165900	-0.36464400	2.39307400
H	8.32955300	2.02593200	3.05720700
H	6.60858800	3.68250700	2.39854200
C	1.74172100	-3.93629600	-0.32330700
C	0.90304400	-4.44526100	-1.34579400
C	1.58581400	-4.39975900	1.00289700
C	-0.05426000	-5.41257500	-1.02071600
C	1.00051500	-3.98245000	-2.80009500
C	0.61119200	-5.36867200	1.27307900
C	2.46747400	-3.91166900	2.15075700
C	-0.21709400	-5.89537800	0.27976000
H	-0.68532300	-5.80367000	-1.81412800
H	1.72387100	-3.16507000	-2.84551900
C	-0.33693500	-3.41778700	-3.30933400
C	1.50891100	-5.10682000	-3.72373000
H	0.50828200	-5.74167500	2.29029200
H	3.16381600	-3.17061300	1.75160100
C	1.63990400	-3.20892100	3.24286500
C	3.31979100	-5.05296100	2.73925600
C	-1.22672200	-6.98961400	0.60592900
H	-0.22066400	-3.02056000	-4.32380100
H	-1.12020600	-4.18341000	-3.34382700
H	-0.67242900	-2.60620600	-2.66261300
H	1.60463700	-4.74355600	-4.75313100
H	2.48740200	-5.48091400	-3.40597700
H	0.81813900	-5.95776900	-3.73432600
H	0.95556300	-3.90754100	3.73878500
H	2.29912100	-2.79114000	4.01259500
H	1.05090400	-2.39646300	2.81068300
H	3.94230300	-5.51988100	1.96909800
H	3.98177400	-4.67167900	3.52501100
H	2.69610000	-5.83696900	3.18312600
H	-1.25480200	-7.08523700	1.70014700
C	-2.65251800	-6.64897600	0.13435600
C	-0.77590400	-8.34992400	0.03771200
H	-3.35741100	-7.42772300	0.44700200
H	-2.99752700	-5.69408800	0.54342900
H	-2.70556300	-6.57839500	-0.95738000

H	0.21841100	-8.62092000	0.40584900
H	-1.47511200	-9.14461100	0.32223700
H	-0.72849000	-8.32006400	-1.05646500
C	2.17236900	3.85816200	0.05470600
C	1.47576700	4.45563400	1.13568700
C	1.94889600	4.32449200	-1.26252000
C	0.60102900	5.51793800	0.87703800
C	1.63764600	3.99165500	2.58484000
C	1.05531500	5.38424300	-1.46426500
C	2.68358700	3.75324700	-2.47460500
C	0.38150900	6.00957500	-0.41195600
H	0.08319100	5.97810600	1.71390600
H	2.31217400	3.13285300	2.59044900
C	0.30359700	3.50894900	3.18366900
C	2.27272400	5.08550800	3.46564400
H	0.89934300	5.75402400	-2.47564700
H	3.33469600	2.94716600	-2.12968500
C	1.71079800	3.13795700	-3.49788400
C	3.59274100	4.81112200	-3.13142700
C	-0.50133200	7.22478800	-0.67359800
H	0.45381200	3.16151600	4.21234700
H	-0.44681600	4.30679900	3.21145500
H	-0.09176200	2.67667100	2.59802000
H	2.42579800	4.71534100	4.48562900
H	3.24342100	5.40664100	3.07394600
H	1.63270900	5.97279800	3.52779300
H	1.02940600	3.89149600	-3.90991800
H	2.26694300	2.70272100	-4.33584100
H	1.11604300	2.34572400	-3.03640300
H	4.30952400	5.22002800	-2.41186000
H	4.15770300	4.36818100	-3.95929700
H	3.01313100	5.64812400	-3.53612500
H	-0.69904900	7.24902800	-1.75405900
C	-1.86102800	7.16288400	0.04371400
C	0.24433500	8.52631000	-0.31446100
H	-2.48081500	8.02062100	-0.24025400
H	-2.40756000	6.24895000	-0.20650900
H	-1.74440300	7.19387400	1.13245800
H	1.19010200	8.60384700	-0.85962700
H	-0.36431100	9.40506100	-0.55665100
H	0.47354200	8.55981200	0.75648800
H	-4.08287100	0.70396600	-6.13885300
C	-2.64034500	-0.46652900	5.97674000
C	-1.35189100	-0.46876800	5.41942100

C	-1.14148900	-0.13919300	4.08260400
C	-2.25647200	0.20724800	3.30541900
C	-3.54711400	0.21611700	3.86723400
C	-3.75181100	-0.12870900	5.20202100
S	-4.73095800	0.72669800	2.66842400
N	-2.22914800	0.57982900	1.97959600
C	-3.45610400	0.74850700	1.35977600
C	-3.56557000	1.76170500	0.25497100
C	-2.42858300	2.46626400	-0.11107000
C	-2.45733300	3.42554800	-1.14590200
C	-3.63450800	3.70016200	-1.79819900
C	-4.82809200	3.00791400	-1.46223700
C	-4.80392700	1.99850500	-0.44098900
C	-6.04314100	3.29292000	-2.13864900
C	-7.20048500	2.60905300	-1.84462900
C	-7.17620100	1.59223400	-0.86582800
C	-6.01135400	1.29152600	-0.18968600
H	-2.77769600	-0.72948000	7.02086300
H	-0.50104200	-0.73534400	6.03879000
H	-0.15490200	-0.13707500	3.63235100
H	-4.74843300	-0.12481700	5.63168600
H	-1.28979200	0.58786600	1.47425600
H	-1.48673200	2.29253900	0.39439700
H	-1.53964500	3.94346000	-1.40382700
H	-3.66965800	4.44663800	-2.58719900
H	-6.03843700	4.06614400	-2.90227400
H	-8.12405500	2.83963700	-2.36714300
H	-8.08247200	1.03557800	-0.64451200
H	-6.02735500	0.47546600	0.52260100

TS*RR*

0 1

H	-1.57351100	-1.49310300	-1.08680300
C	-1.02238500	-3.17916000	0.73223800
C	-2.31841600	-2.75285600	0.39762000
C	-3.40386500	-3.15160800	1.19796800
C	-3.18905900	-3.97799900	2.29724600
C	-1.90326400	-4.41159500	2.62396600
C	-0.82505200	-4.00046400	1.83816800
N	-2.44926100	-1.92034800	-0.73240500
C	-3.60677500	-1.49856200	-1.33101400
H	-0.17889800	-2.86278200	0.13093400
H	-4.40422200	-2.80491000	0.98738600
H	-4.03779200	-4.27094200	2.90865700

H	0.18113500	-4.32100500	2.07520400
C	-3.34796200	-0.84719900	-2.69414300
O	-2.26603200	-0.81682700	-3.22763000
O	-4.47941000	-0.33688500	-3.20167100
C	-4.33925900	0.28265700	-4.49623300
H	-3.93902600	-0.43370000	-5.21706300
H	-3.67194700	1.14415700	-4.43094200
C	-4.80540600	-2.28713400	-1.29856600
C	-5.84767000	-2.90339600	-1.35251200
H	-5.34460600	0.59377200	-4.77770600
C	-7.09341200	-3.65632200	-1.37062100
H	-7.76100900	-3.31334300	-0.57139100
H	-7.61612000	-3.53610100	-2.32559600
H	-6.90741600	-4.72493800	-1.21752400
H	-1.74174900	-5.05165700	3.48626600
P	0.78401900	-0.04029700	-0.00841200
O	-0.15785700	0.38251700	1.09571500
O	0.20203800	-0.61573000	-1.26741900
O	1.91998600	-1.07211900	0.64576100
O	1.73716200	1.26523700	-0.45687900
C	3.48451200	-2.49259900	-0.56616700
C	3.14810300	-1.23302700	0.02081300
C	4.05966600	-0.18714600	0.07346200
C	4.72000600	-2.61106300	-1.17234100
H	5.00425800	-3.57179700	-1.59240800
C	5.63580400	-1.53601900	-1.26393400
C	5.30625700	-0.29513800	-0.63530100
C	6.22299100	0.78536900	-0.77394800
C	7.40381600	0.63199100	-1.46662100
C	7.74022900	-0.60911300	-2.05742500
C	6.86876100	-1.66850200	-1.95834000
H	5.98057300	1.74551400	-0.33520200
H	8.08200900	1.47504200	-1.56403000
H	8.67767700	-0.71747500	-2.59525300
H	7.10409400	-2.62452700	-2.41934800
C	2.62024200	1.80012600	0.46644300
C	3.73517000	1.05420500	0.82512000
C	3.31491100	3.61576100	1.88173900
C	2.43060200	3.13792300	0.93370800
H	3.21437600	4.64306900	2.21895900
C	4.35889400	2.82614600	2.42311200
C	4.58573200	1.52219200	1.88287900
C	5.63441700	0.73811200	2.43942800
C	6.41958800	1.22615700	3.46067000

C	6.20785000	2.52746900	3.97558300
C	5.19686900	3.30837900	3.46462000
H	5.80329700	-0.26244300	2.05855100
H	7.20692300	0.60471200	3.87785600
H	6.83819900	2.90188000	4.77703200
H	5.01596900	4.30592300	3.85724100
C	2.64263000	-3.73266300	-0.46581100
C	1.84625700	-4.15875400	-1.55731800
C	2.78067100	-4.55690900	0.67500600
C	1.20593500	-5.40041800	-1.47611200
C	1.71618000	-3.32163800	-2.83115700
C	2.10970300	-5.78673600	0.70505200
C	3.66147600	-4.16323500	1.86249300
C	1.32145800	-6.23124500	-0.35711400
H	0.60034300	-5.72931700	-2.31540200
H	1.95917900	-2.28747700	-2.57217800
C	0.29145100	-3.30553400	-3.41014000
C	2.71144600	-3.79903700	-3.90951900
H	2.21461400	-6.42431200	1.58027000
H	4.17841200	-3.23658800	1.60141900
C	2.82954700	-3.87033500	3.12549900
C	4.74938400	-5.21665000	2.14560700
C	0.62196800	-7.58354200	-0.29477700
H	0.23521000	-2.58563200	-4.23235200
H	-0.00261500	-4.28329600	-3.80990800
H	-0.44262500	-2.99867200	-2.66434000
H	2.61663400	-3.18791600	-4.81417600
H	3.74770300	-3.73266600	-3.56742800
H	2.51547500	-4.84189300	-4.18575000
H	2.28300400	-4.76019800	3.46015100
H	3.48010500	-3.55442000	3.94886900
H	2.10682800	-3.07047900	2.94019300
H	5.36183400	-5.40234000	1.25749200
H	5.41167000	-4.87511300	2.94903700
H	4.31868700	-6.17385600	2.45911400
H	0.84840200	-8.01648900	0.68896500
C	-0.90882600	-7.44853500	-0.39813600
C	1.16446000	-8.55465500	-1.36079600
H	-1.39153500	-8.42629700	-0.28676900
H	-1.30039900	-6.78045400	0.37454900
H	-1.20572200	-7.04106200	-1.37074600
H	2.24658300	-8.68666700	-1.26212700
H	0.69038000	-9.53841100	-1.26833300
H	0.96566300	-8.18304200	-2.37210100

C	1.43429700	4.10098800	0.35356300
C	0.31708200	4.53142900	1.10880000
C	1.71310400	4.70563400	-0.89537600
C	-0.47521000	5.57377900	0.61250000
C	-0.03184300	3.91434200	2.46430200
C	0.87618900	5.73100800	-1.35160700
C	2.93076800	4.32626400	-1.73966300
C	-0.21296800	6.19405900	-0.61130400
H	-1.31794300	5.91492900	1.20617900
H	0.49568100	2.95862600	2.53804400
C	-1.53437700	3.61251700	2.60545400
C	0.42439900	4.81082300	3.63375500
H	1.09525300	6.20467500	-2.30636300
H	3.46437600	3.52292800	-1.22704400
C	2.53706600	3.78231900	-3.12552700
C	3.91462700	5.50643200	-1.86540300
C	-1.03181400	7.37930400	-1.10942600
H	-1.71719900	3.03786900	3.51960500
H	-2.13340600	4.52718000	2.67550800
H	-1.90552500	3.03113900	1.75891400
H	0.16411800	4.35124900	4.59413200
H	1.50441700	4.97754200	3.62517200
H	-0.06558800	5.79027100	3.58682400
H	1.99315500	4.53024500	-3.71437900
H	3.43136900	3.50371800	-3.69385500
H	1.91105800	2.89029700	-3.03131200
H	4.22895600	5.86496500	-0.88000400
H	4.81058000	5.20163300	-2.41792300
H	3.46617100	6.35122900	-2.39982200
H	-0.80773400	7.49413100	-2.17884000
C	-2.55033700	7.17488700	-0.97726900
C	-0.59139500	8.67898200	-0.40525500
H	-3.08888300	8.01375500	-1.43232500
H	-2.87104900	6.25211900	-1.46891500
H	-2.86093300	7.12172300	0.07191200
H	0.47989600	8.85832400	-0.53949900
H	-1.13592400	9.54379700	-0.80156900
H	-0.78652300	8.62216000	0.67155900
C	-2.65983800	3.91559900	-3.31795000
C	-1.41875300	3.36278000	-2.96756700
C	-1.29610300	2.44766200	-1.92644800
C	-2.45237600	2.08775900	-1.22386900
C	-3.69906300	2.64054200	-1.57941900
C	-3.81669200	3.55356000	-2.62663100

S	-4.97747300	2.01318400	-0.54988700
N	-2.53938300	1.19489700	-0.16344000
C	-3.82693500	0.78549100	0.16044000
C	-4.11122500	0.30420200	1.55695900
C	-3.08798800	0.30198500	2.49181700
C	-3.31680500	-0.10142000	3.82542500
C	-4.56991000	-0.48881700	4.23038300
C	-5.64713400	-0.52598600	3.30600600
C	-5.42482600	-0.15092500	1.93689800
C	-6.94085700	-0.94289800	3.71861700
C	-7.98658300	-1.01090600	2.82726500
C	-7.76740100	-0.67743100	1.47232000
C	-6.52348000	-0.26595200	1.03938100
H	-2.72479500	4.62546400	-4.13680400
H	-0.53065500	3.64463100	-3.52177600
H	-0.33999500	2.00311400	-1.68466700
H	-4.78267800	3.96985800	-2.89434100
H	-4.01073900	-0.30500600	-0.59802200
H	-2.08153700	0.59358200	2.21741800
H	-2.48730600	-0.09014200	4.52510600
H	-4.75551800	-0.78161300	5.26039800
H	-7.08705500	-1.21174500	4.76150600
H	-8.97153000	-1.32719200	3.15751300
H	-8.58576800	-0.74426300	0.76089900
H	-6.39012500	-0.05061600	-0.01336900
H	-1.67947800	0.80852000	0.28976100

References

1. J.-X. Ji, T. T.-L. Au-Yeung, J. Wu, C. W. Yip, A. S. C. Chan, *Adv. Synth. Catal.*, 2004, **346**, 42.

2. Q. Kang, Z.-A. Zhao, S.-L. You, *Org. Lett.*, 2008, **10**, 2031.

3. M. J. Frisch, G. W. Trucks, H. B. Schlegel, G. E. Scuseria,M. A. Robb, J. R. Cheeseman, G. Scalmani, V. Barone,B. Mennucci, G. A. Petersson, H. Nakatsuji, M. Caricato,X. Li, H. P. Hratchian, A. F. Izmaylov, J. Bloino, G. Zheng,J. L. Sonnenberg, M. Hada, M. Ehara, K. Toyota, R. Fukuda,J. Hasegawa, M. Ishida, T. Nakajima, Y. Honda, O. Kitao,H. Nakai, T. Vreven, J. A. Montgomery Jr., J. E. Peralta,F. Ogliaro, M. Bearpark, J. J. Heyd, E. Brothers,K. N. Kudin, V. N. Staroverov, R. Kobayashi, J. Normand,K. Raghavachari, A. Rendell, J. C. Burant, S. S. Iyengar,J. Tomasi, M. Cossi, N. Rega, J. M. Millam, M. Klene,J. E. Knox, J. B. Cross, V. Bakken, C. Adamo, J. Jaramillo,R. Gomperts, R. E. Stratmann, O. Yazyev, A. J. Austin,R. Cammi, C. Pomelli, J. W. Ochterski, R. L. Martin,K. Morokuma, V. G. Zakrzewski, G. A. Voth, P. Salvador,J. J. Dannenberg, S. Dapprich, A. D. Daniels, Ö. Farkas,J. B. Foresman, J. V. Ortiz, J. Cioslowski and D. J. Fox, Gaussian 09, Revision B.01, Gaussian, Inc., Wallingford, CT, 2010.

4. (a) A. D. Becke, *J. Chem. Phys.*, 1993, **98**, 5648; (b) C. Lee, W. Yang, R. G. Parr, *Phys. Rev. B: Condens. Matter Mater. Phys.*, 1988, **37**, 785.

5. (a) K. A. Peterson, D. Figgen, M. Dolg, H. Stoll, *J. Chem. Phys.*, 2007, **126**, 124101; (b) D. Figgen, K. A. Peterson, M. Dolg, H. Stoll, *J. Chem. Phys.*, 2009, **130**, 164108.

6. (a) Y. Zhao, D. G. Truhlar, *Theor. Chem. Acc.*, 2008, **120**, 215; (b) Y. Zhao, D. G. Truhlar, *Acc. Chem. Res.*, 2008, **41**, 157.

7. J. Tomasi, B. Mennucci, R. Cammi, *Chem. Rev.*, 2005, **105**, 2999.

8. (a) T. Lu, F. Chen, *J. Comput. Chem.*, 2012, **33**, 580; (b) S. Manzetti, T.Lu, *J. Phys. Org. Chem.*, 2013, **26**, 473.

9. W. Humphrey, A. Dalke, K. Schulten, *J. Mol. Graphics*, 1996, **14**, 33.

10. C. Y. Legault, *CYLview, 1.0b*, Université de Sherbrooke, Canada, 2009, http://www.cylview.org.

NMR spectra

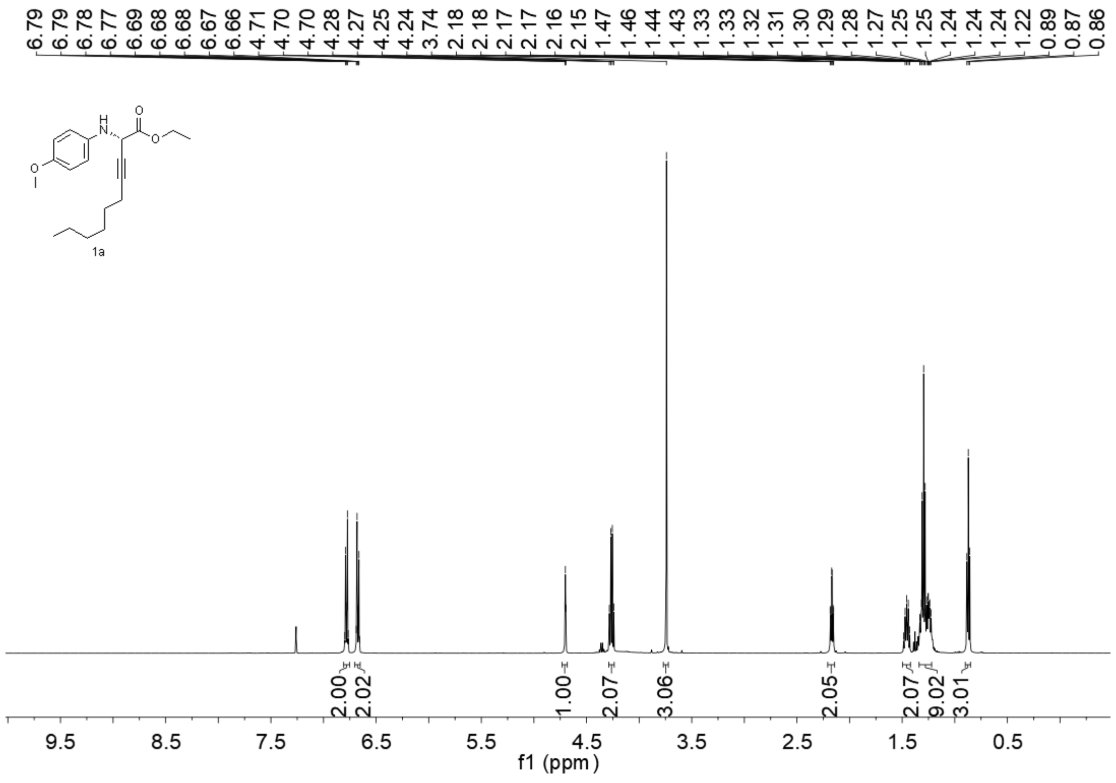

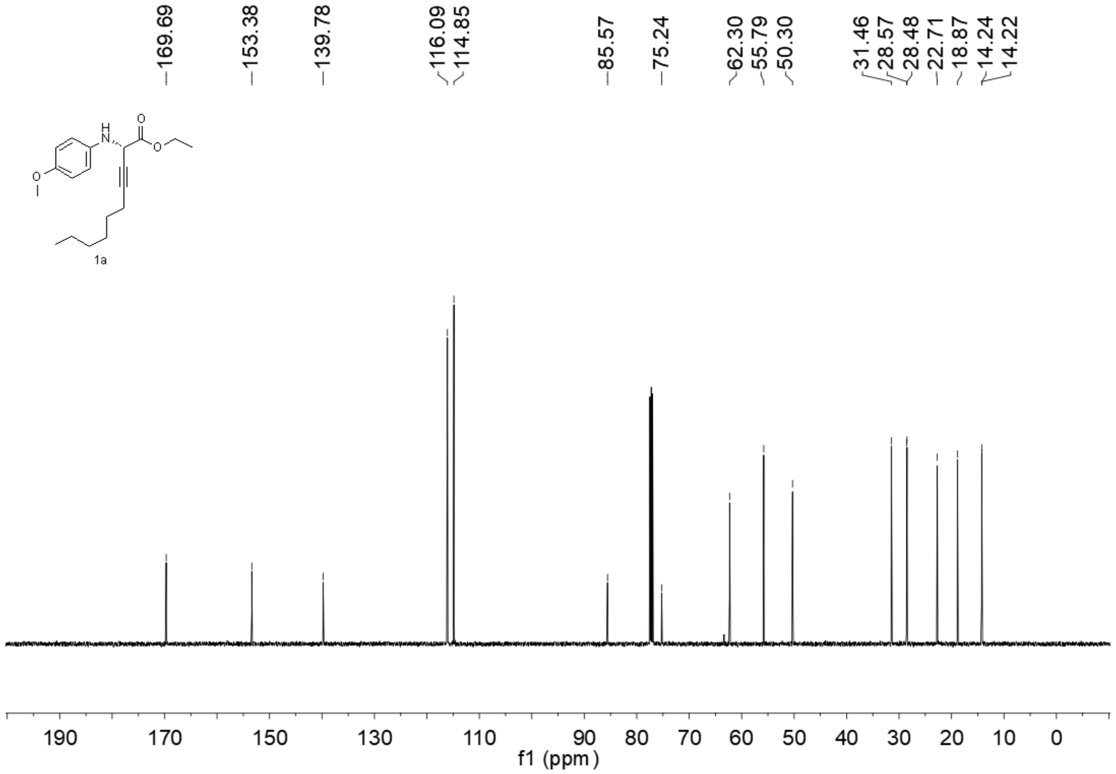

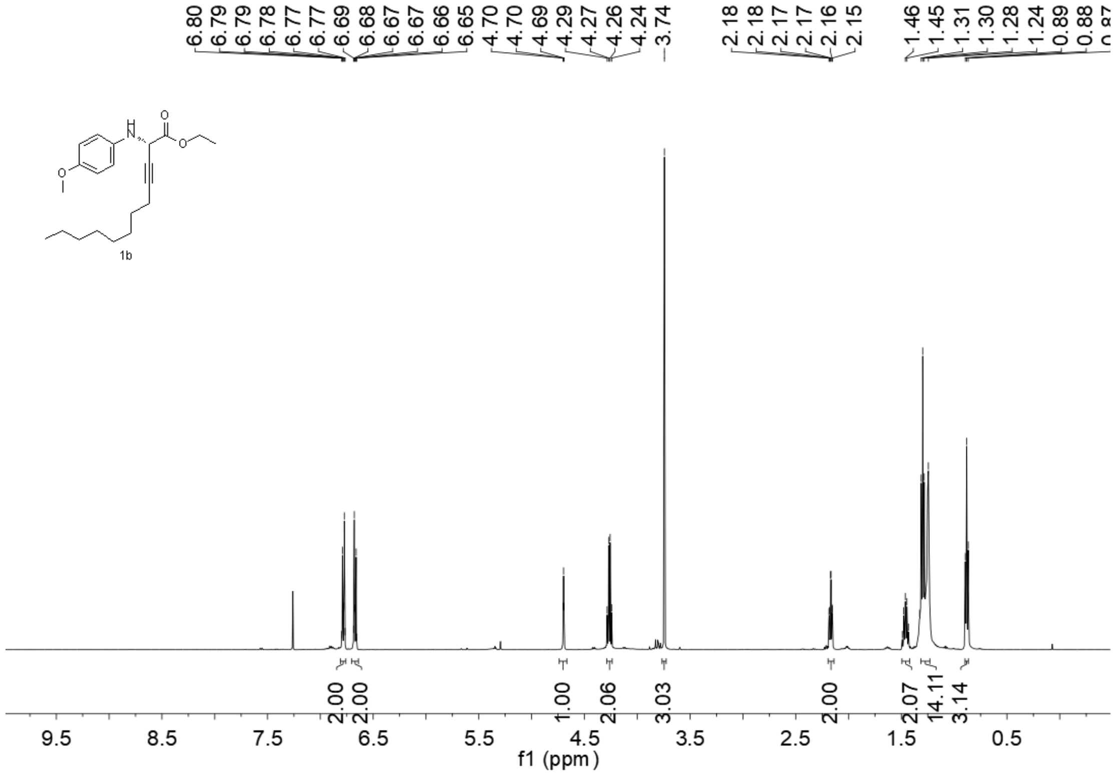
6.80
6.79
6.79
6.78
6.77
6.77
6.69
6.68
6.67
6.67
6.66
6.65
4.70
4.70
4.69
4.29
4.27
4.26
4.24
3.74
2.18
2.18
2.17
2.17
2.16
2.15
1.46
1.45
1.31
1.30
1.28
1.24
0.89
0.88
1b
2.00
2.00
1.00
2.06
3.03
2.00
2.07
14.11
3.14
9.5
8.5
7.5
6.5
5.5
4.5
3.5
2.5
1.5
0.5
f1 (ppm)

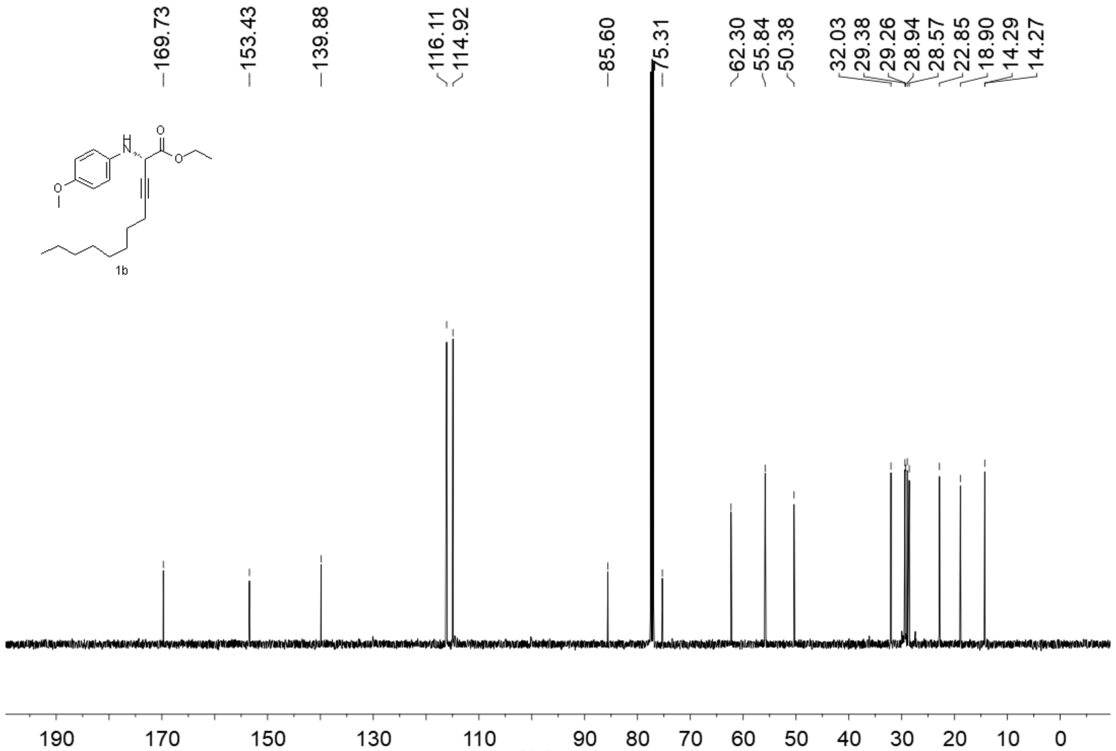
169.73
153.43
139.88
116.11
114.92
85.60
75.31
62.30
55.84
50.38
32.03
29.38
29.26
28.94
28.57
22.85
18.90
14.29
14.27
1b
190
170
150
130
110
90
80
70
60
50
40
30
20
10
0
f1 (ppm)

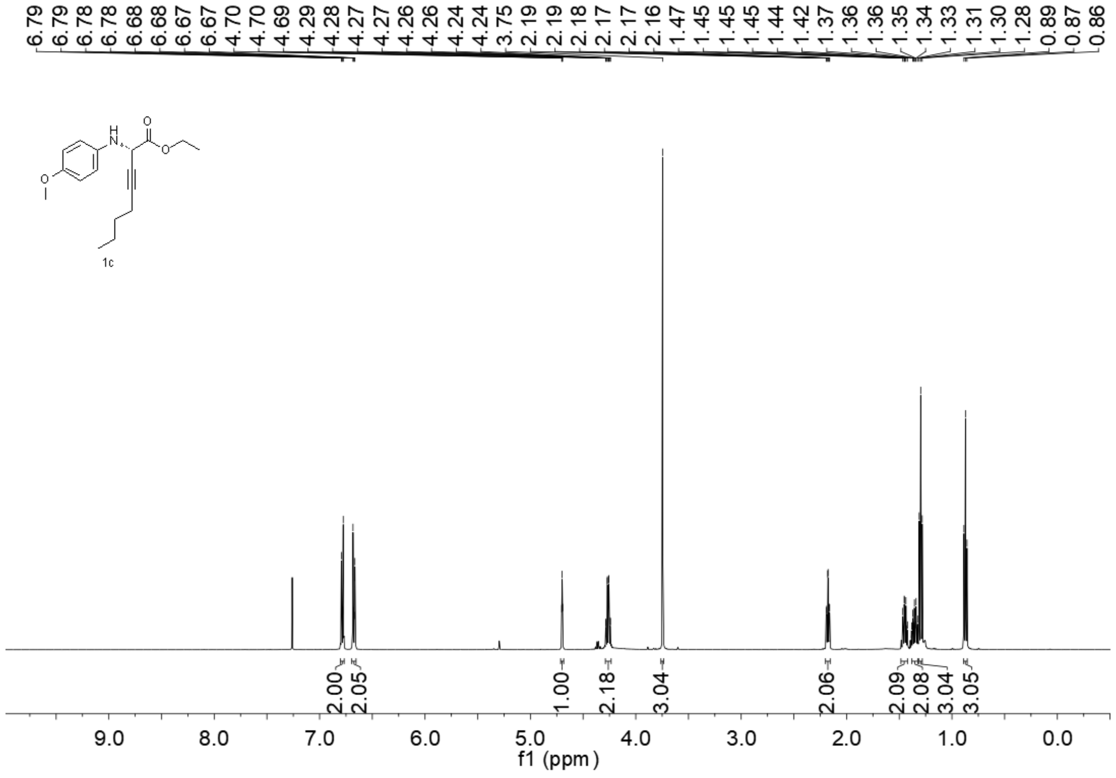
6.79 6.79 6.78 6.78 6.68 6.68 6.67 6.67 4.70 4.70 4.69 4.29 4.28 4.27 4.27 4.26 4.26 4.24 4.24 3.75 2.19 2.19 2.18 2.17 2.17 2.16 1.47 1.45 1.45 1.45 1.44 1.42 1.37 1.36 1.36 1.35 1.34 1.33 1.31 1.30 1.28 0.89 0.87 0.86
1c
2.00 2.05 1.00 2.18 3.04 2.06 2.09 2.08 3.04 3.05
9.0 8.0 7.0 6.0 5.0 4.0 3.0 2.0 1.0 0.0
f1 (ppm)

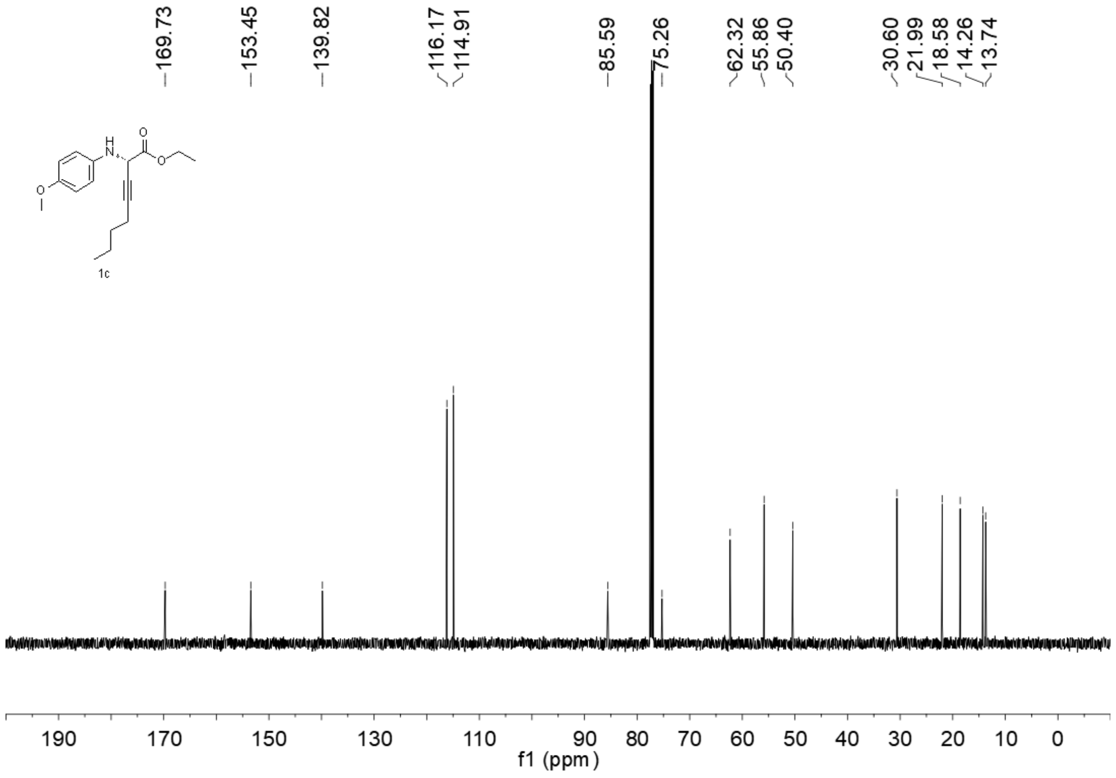
169.73 153.45 139.82 116.17 114.91 85.59 75.26 62.32 55.86 50.40 30.60 21.99 18.58 14.26 13.74
1c
190 170 150 130 110 90 80 70 60 50 40 30 20 10 0
f1 (ppm)

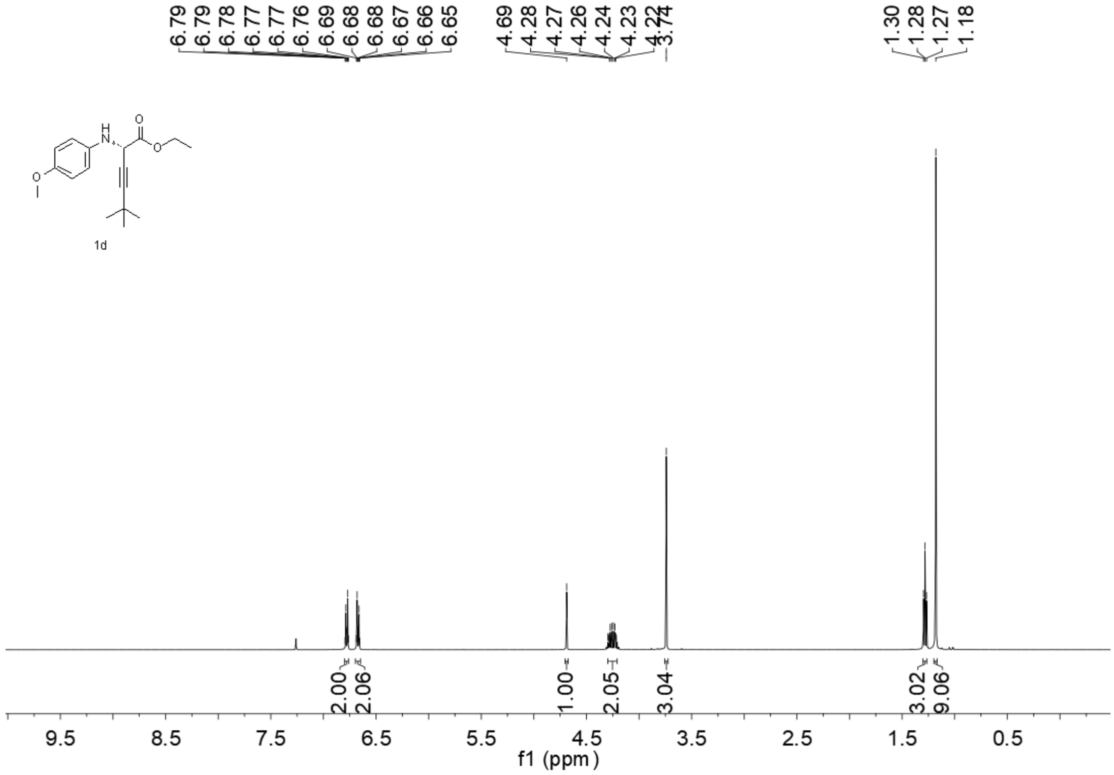
6.79 6.79 6.78 6.77 6.77 6.76 6.69 6.68 6.68 6.67 6.66 6.65
4.69 4.28 4.27 4.26 4.24 4.23 4.22 3.74
1.30 1.28 1.27 1.18
1d
2.00 2.06 1.00 2.05 3.04 3.02 9.06
9.5 8.5 7.5 6.5 5.5 4.5 3.5 2.5 1.5 0.5
f1 (ppm)

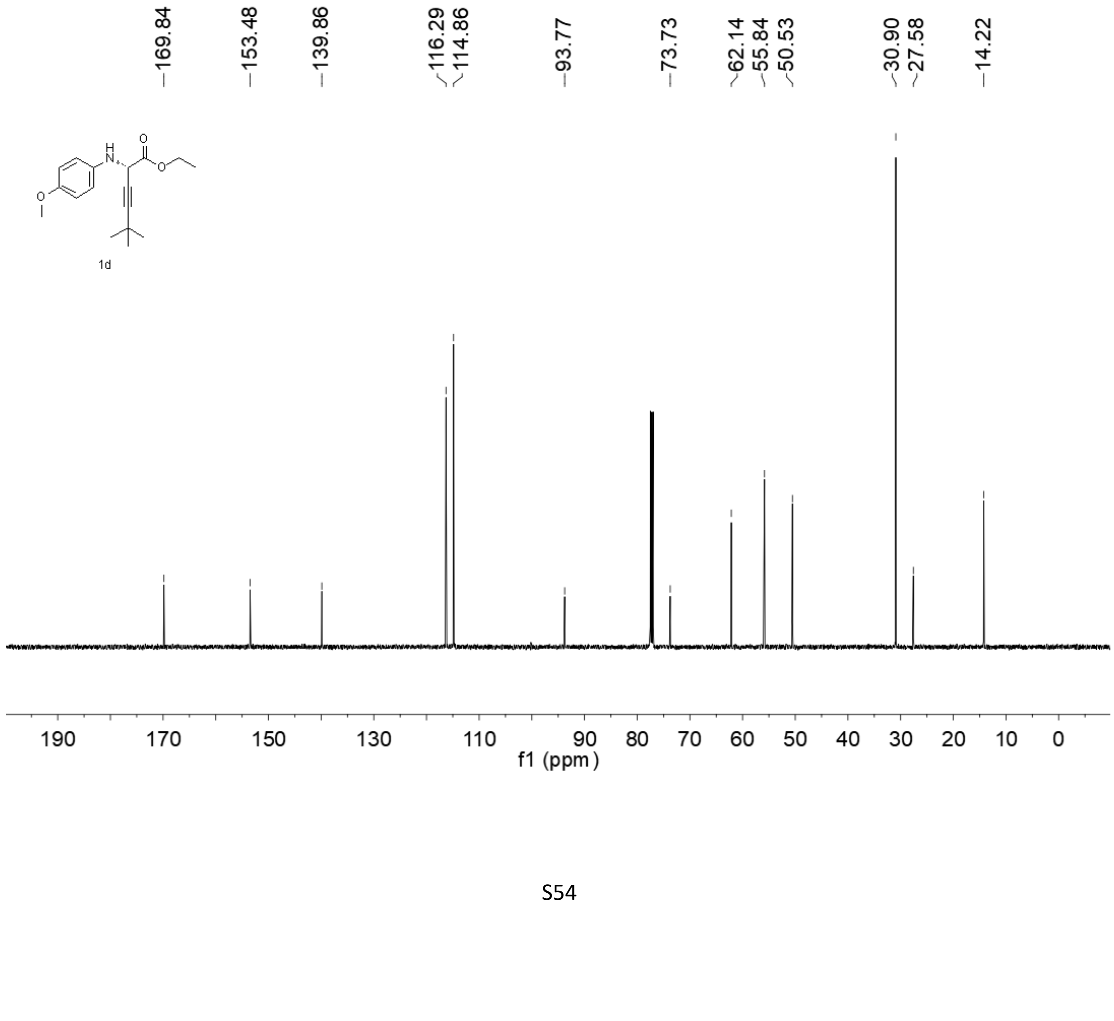
169.84 153.48 139.86 116.29 114.86 93.77 73.73 62.14 55.84 50.53 30.90 27.58 14.22
1d
190 170 150 130 110 90 80 70 60 50 40 30 20 10 0
f1 (ppm)

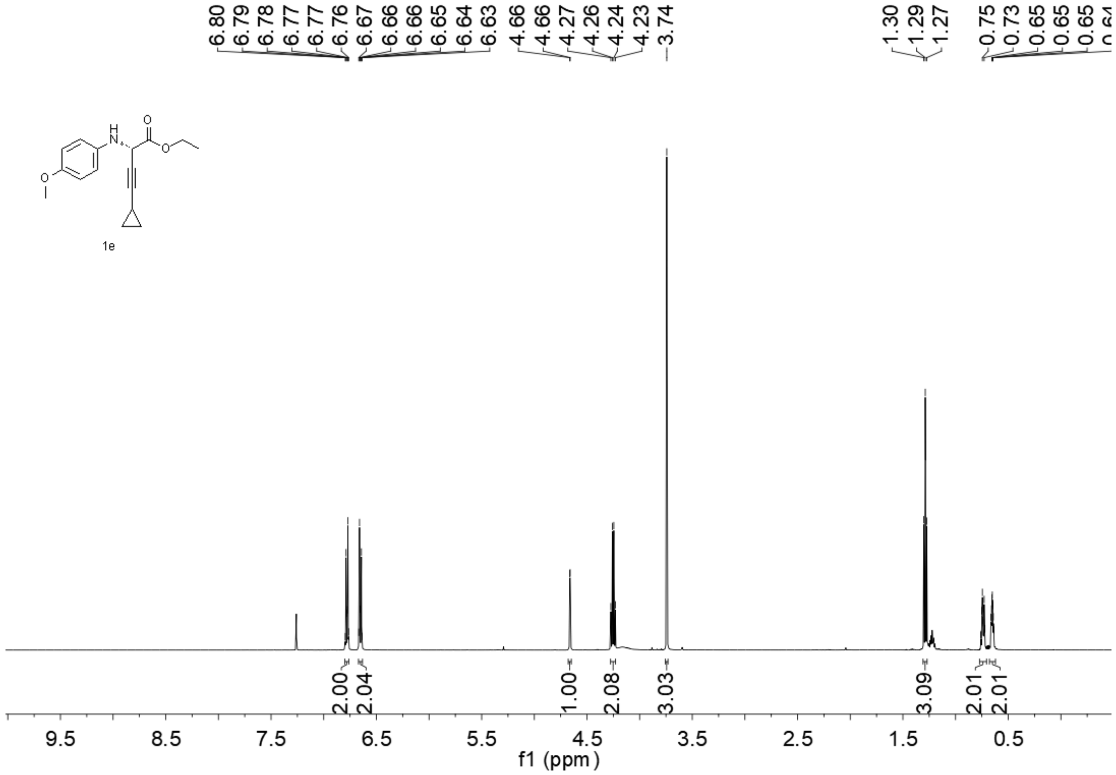
6.80 6.79 6.78 6.77 6.77 6.76 6.67 6.66 6.66 6.65 6.64 6.63 4.66 4.66 4.27 4.26 4.24 4.23 3.74 1.30 1.29 1.27 0.75 0.73 0.65 0.65 0.65
1e
2.00 2.04 1.00 2.08 3.03 3.09 2.01 2.01
9.5 8.5 7.5 6.5 5.5 4.5 3.5 2.5 1.5 0.5
f1 (ppm)

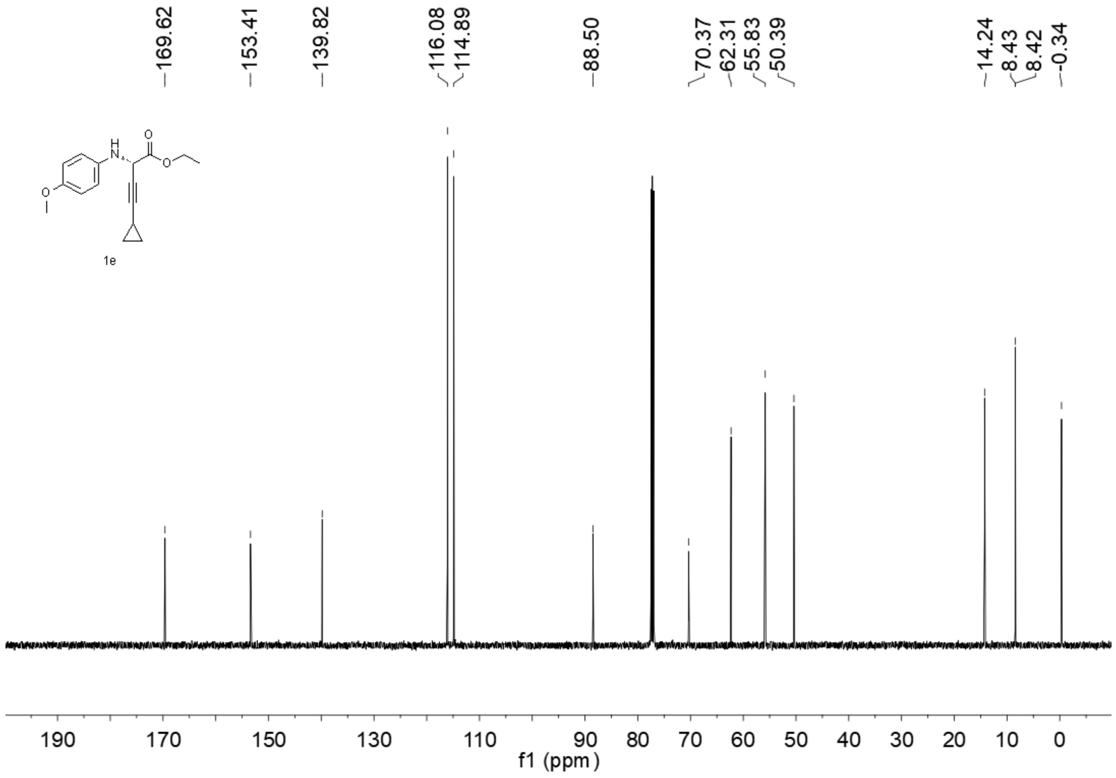
169.62 153.41 139.82 116.08 114.89 88.50 70.37 62.31 55.83 50.39 14.24 8.43 8.42 0.34
1e
190 170 150 130 110 90 80 70 60 50 40 30 20 10 0
f1 (ppm)

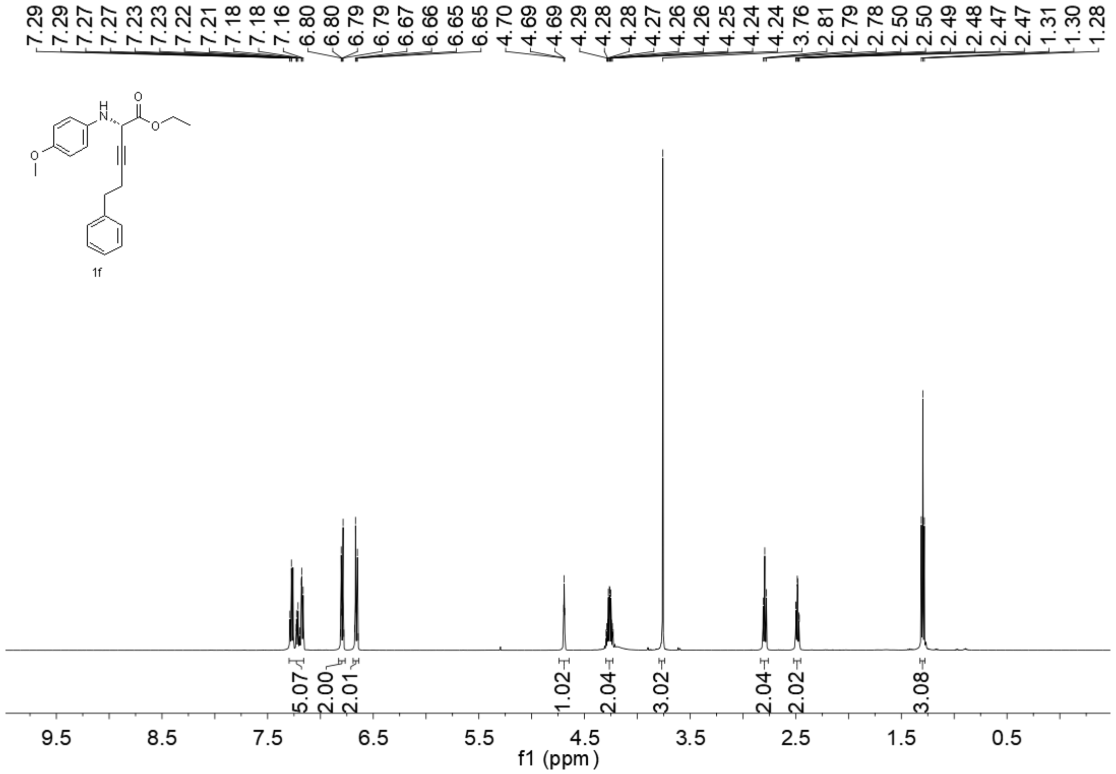
7.29 7.29 7.27 7.27 7.23 7.23 7.22 7.21 7.18 7.18 7.16 6.80 6.80 6.79 6.79 6.67 6.66 6.65 6.65 4.70 4.69 4.69 4.29 4.28 4.28 4.27 4.26 4.26 4.25 4.24 4.24 3.76 2.81 2.79 2.78 2.50 2.50 2.49 2.48 2.47 2.47 1.31 1.30 1.28
H
N
O
O
O
1f
5.07
2.00
2.01
1.02
2.04
3.02
2.04
2.02
3.08
9.5 8.5 7.5 6.5 5.5 4.5 3.5 2.5 1.5 0.5
f1 (ppm)

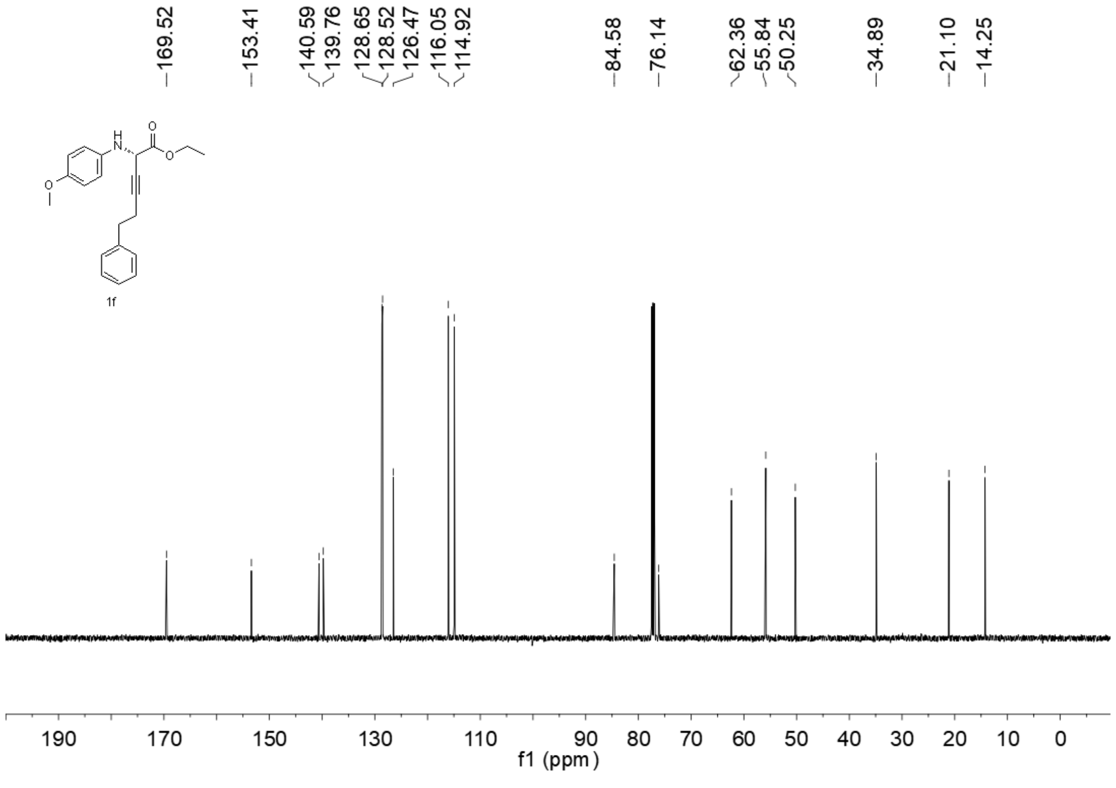
169.52 153.41 140.59 139.76 128.65 128.52 126.47 116.05 114.92 84.58 76.14 62.36 55.84 50.25 34.89 21.10 14.25
H
N
O
O
O
1f
190 170 150 130 110 90 80 70 60 50 40 30 20 10 0
f1 (ppm)

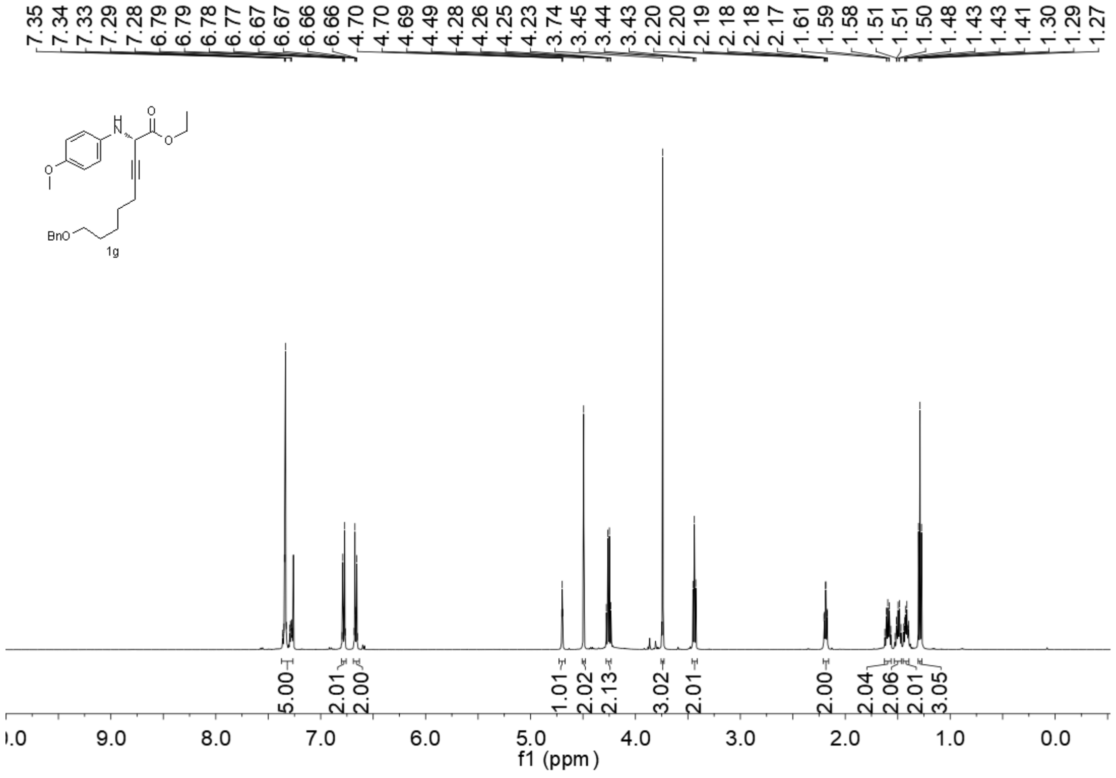

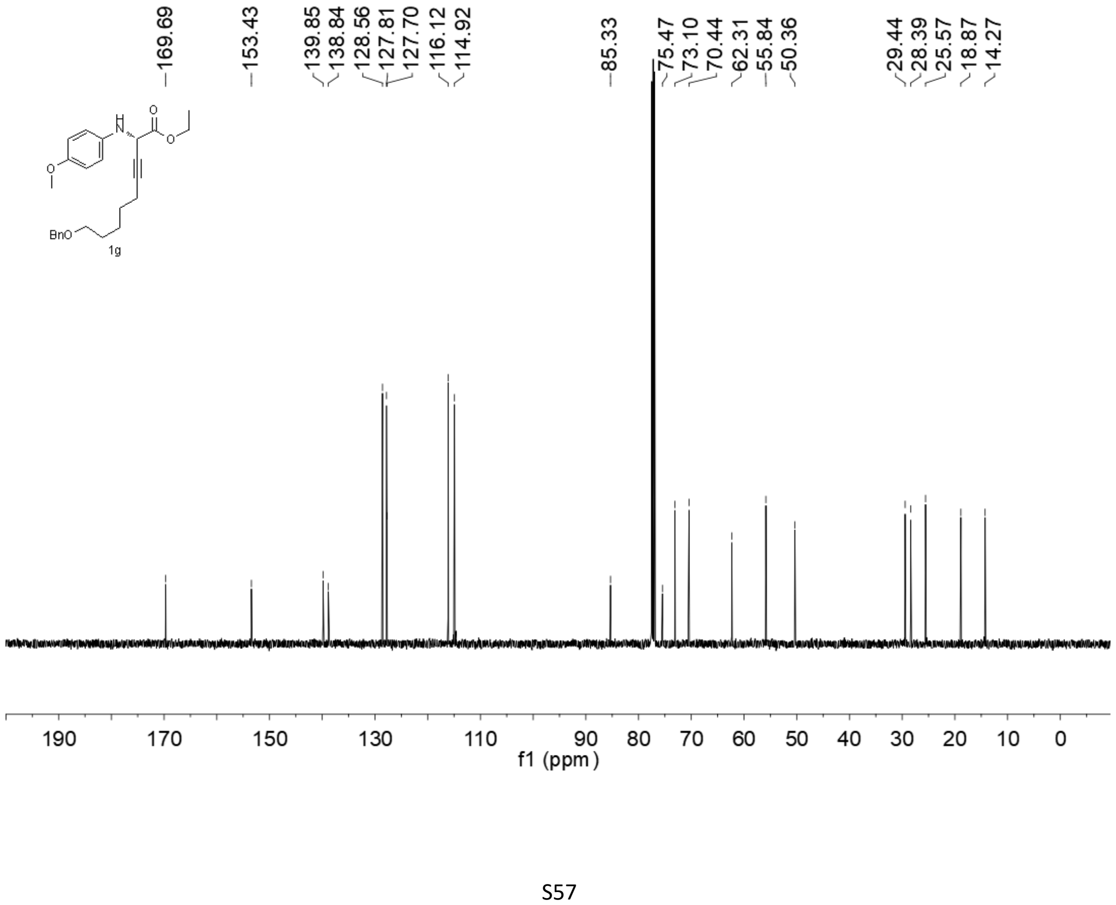

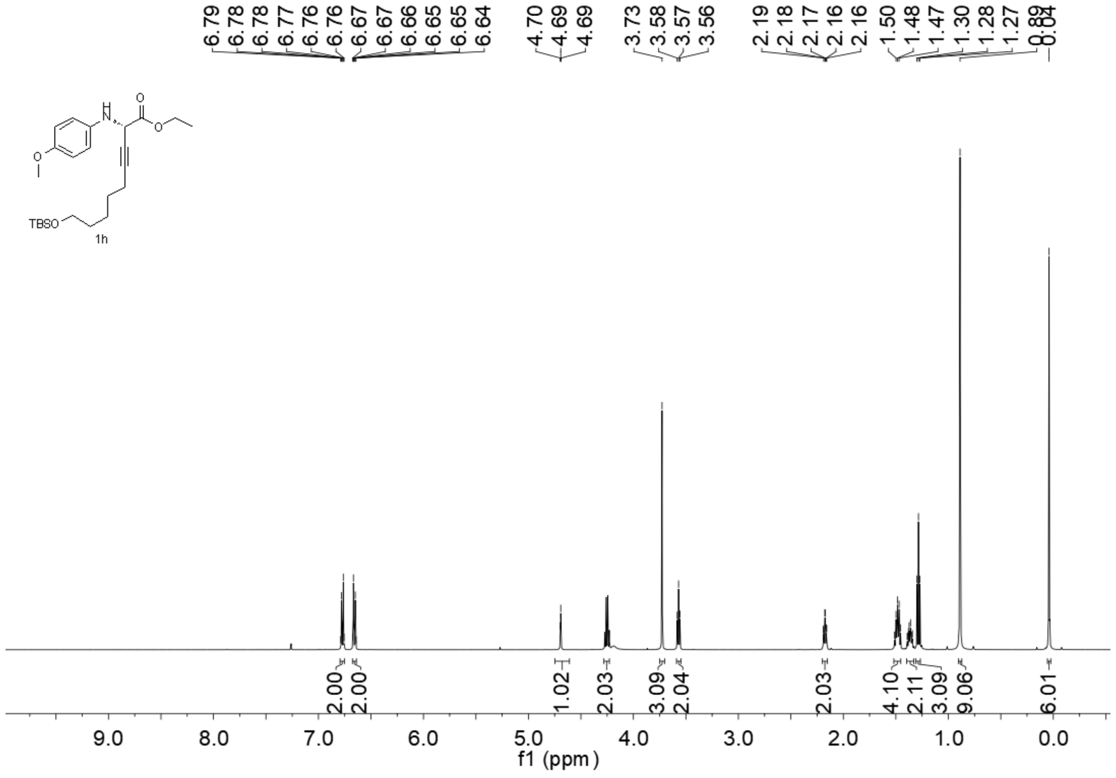
6.79 6.78 6.78 6.77 6.76 6.76 6.67 6.67 6.66 6.65 6.65 6.64
4.70 4.69 4.69
3.73 3.58 3.57 3.56
2.19 2.18 2.17 2.16 2.16 1.50 1.48 1.47 1.30 1.28 1.27 0.89 0.04
TBSO
1h
2.00 2.00 1.02 2.03 3.09 2.04 2.03 4.10 2.11 3.09 9.06 6.01
9.0 8.0 7.0 6.0 5.0 4.0 3.0 2.0 1.0 0.0
f1 (ppm)

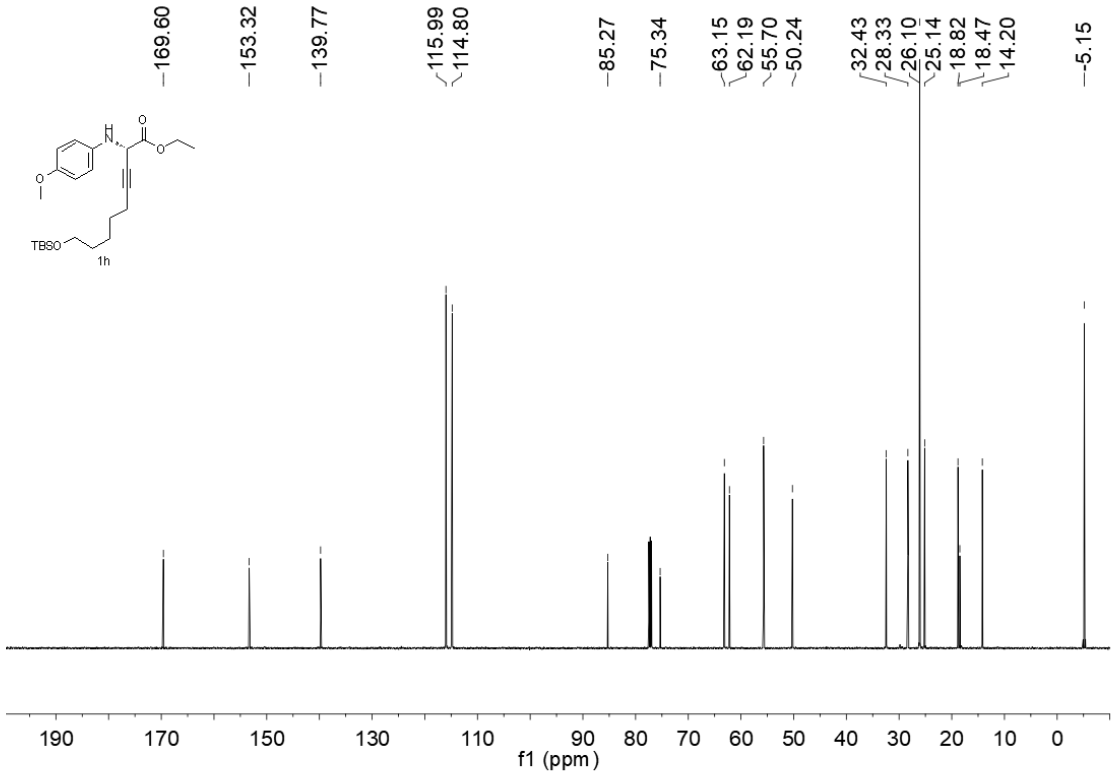
169.60
153.32
139.77
115.99 114.80
85.27
75.34
63.15 62.19 55.70 50.24
32.43 28.33 26.10 25.14 18.82 18.47 14.20
-5.15
TBSO
1h
190 170 150 130 110 90 80 70 60 50 40 30 20 10 0
f1 (ppm)

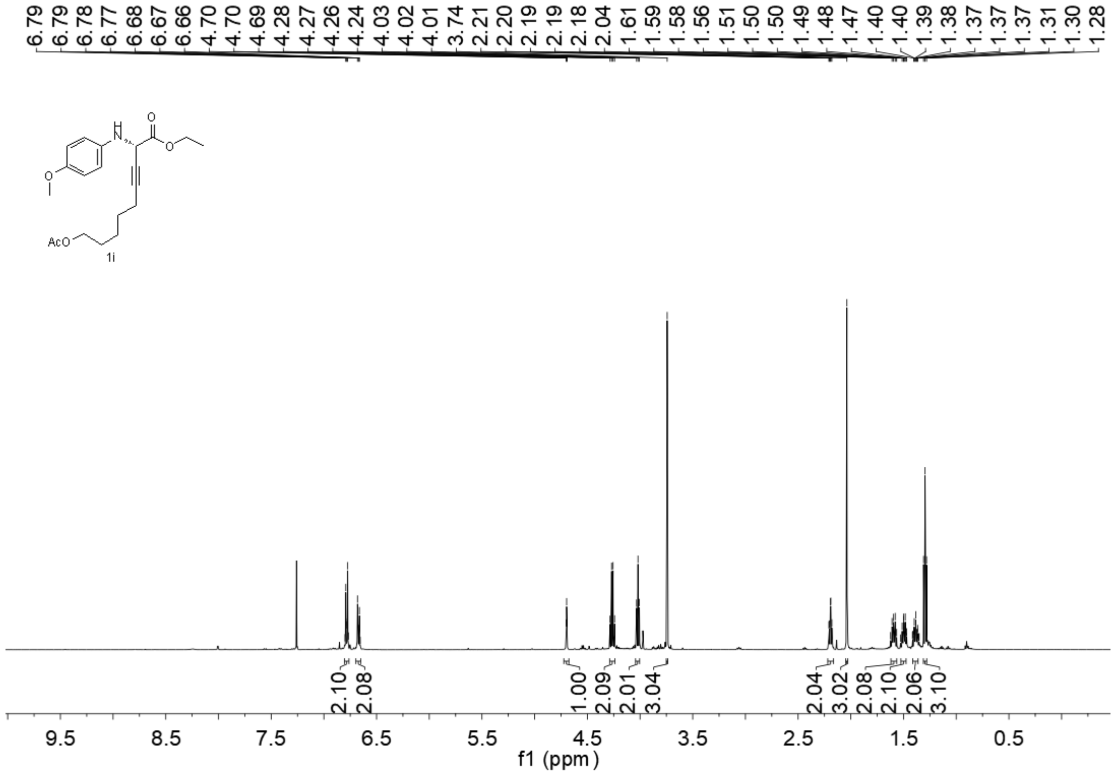
6.79 6.79 6.78 6.77 6.68 6.67 6.66 4.70 4.70 4.69 4.28 4.27 4.26 4.24 4.03 4.02 4.01 3.74 2.21 2.20 2.19 2.19 2.18 2.04 1.61 1.59 1.58 1.56 1.51 1.50 1.50 1.49 1.48 1.47 1.40 1.40 1.39 1.38 1.37 1.37 1.37 1.31 1.30 1.28
AcO
1i
2.10 2.08 1.00 2.09 2.01 3.04 2.04 3.02 2.08 2.10 2.06 3.10
9.5 8.5 7.5 6.5 5.5 4.5 3.5 2.5 1.5 0.5
f1 (ppm)

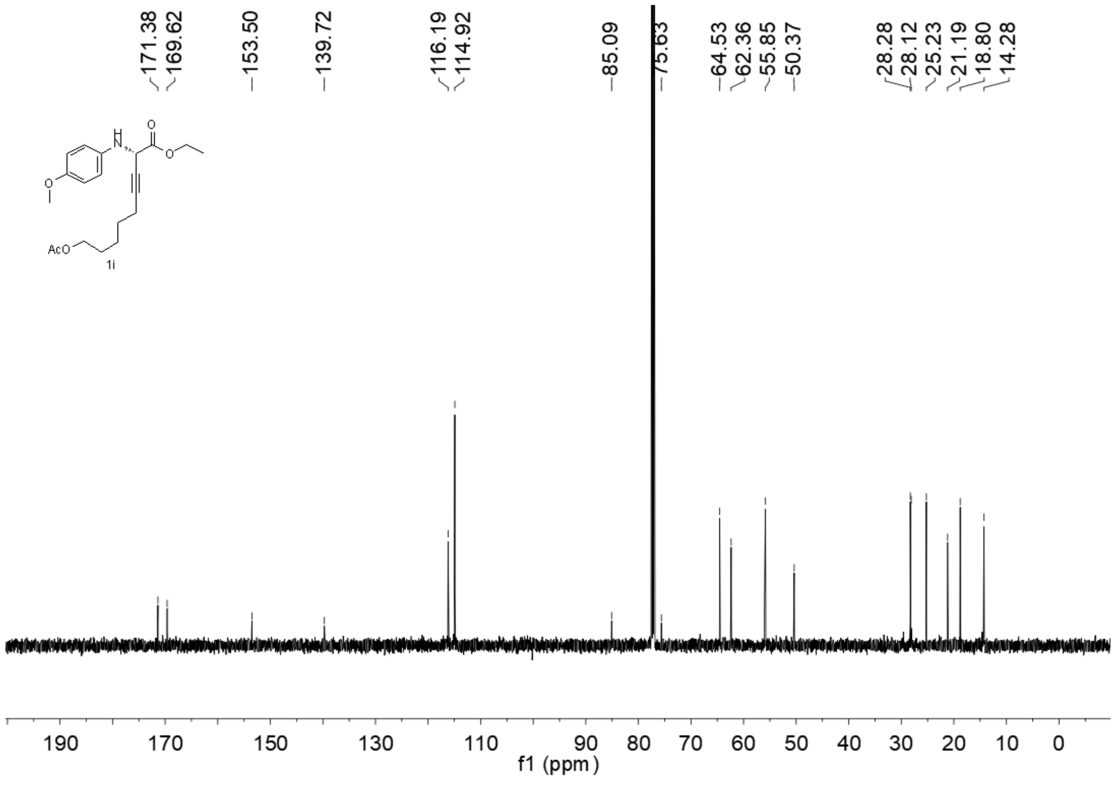
171.38 169.62 153.50 139.72 116.19 114.92 85.09 75.63 64.53 62.36 55.85 50.37 28.28 28.12 25.23 21.19 18.80 14.28
AcO
1i
190 170 150 130 110 90 80 70 60 50 40 30 20 10 0
f1 (ppm)

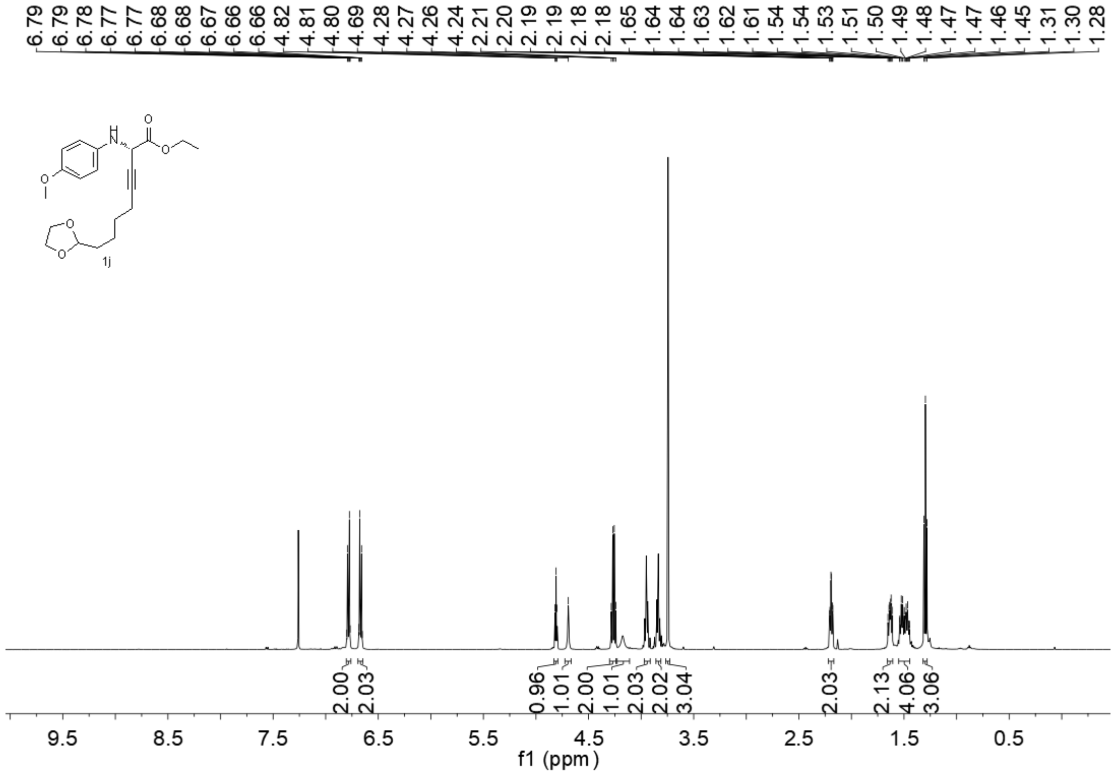
6.79 6.79 6.78 6.77 6.77 6.68 6.68 6.67 6.66 6.66 4.82 4.81 4.80 4.69 4.28 4.27 4.26 4.24 2.21 2.20 2.19 2.19 2.18 2.18 1.65 1.64 1.64 1.63 1.62 1.61 1.54 1.54 1.53 1.51 1.50 1.49 1.48 1.47 1.47 1.46 1.45 1.31 1.30 1.28
1j
2.00 2.03 0.96 1.01 2.00 1.01 2.03 2.02 3.04 2.03 2.13 4.06 3.06
9.5 8.5 7.5 6.5 5.5 4.5 3.5 2.5 1.5 0.5
f1 (ppm)

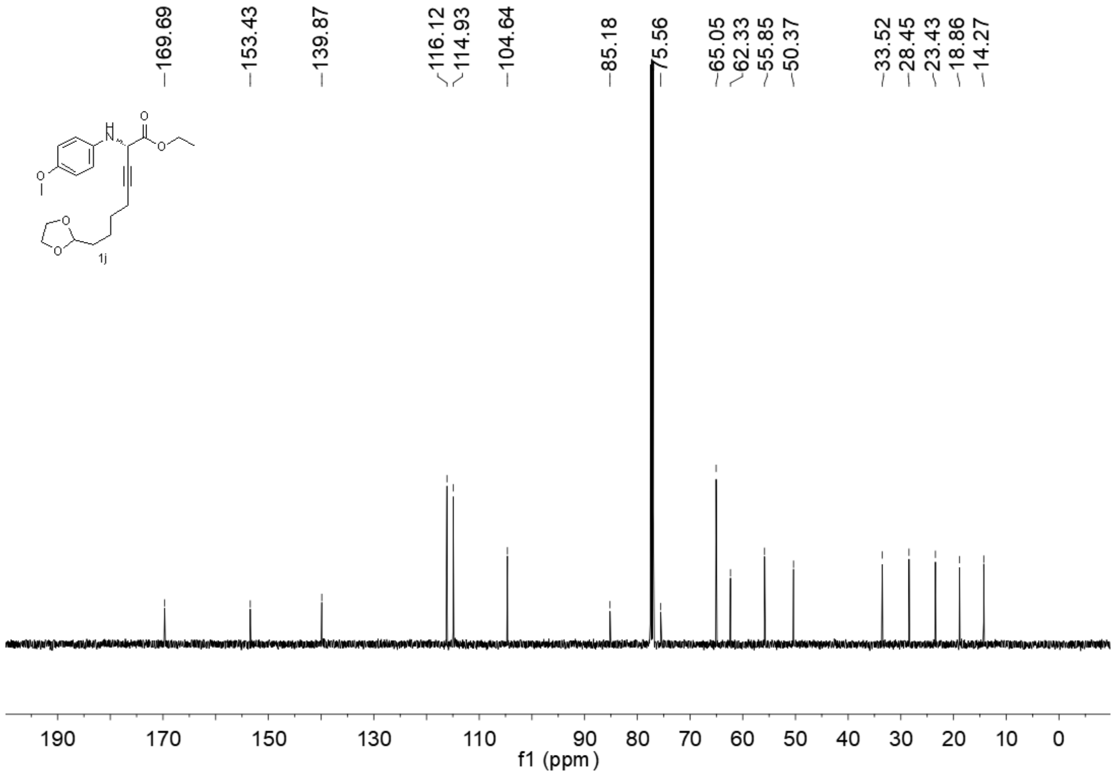
169.69 153.43 139.87 116.12 114.93 104.64 85.18 75.56 65.05 62.33 55.85 50.37 33.52 28.45 23.43 18.86 14.27
1j
190 170 150 130 110 90 80 70 60 50 40 30 20 10 0
f1 (ppm)

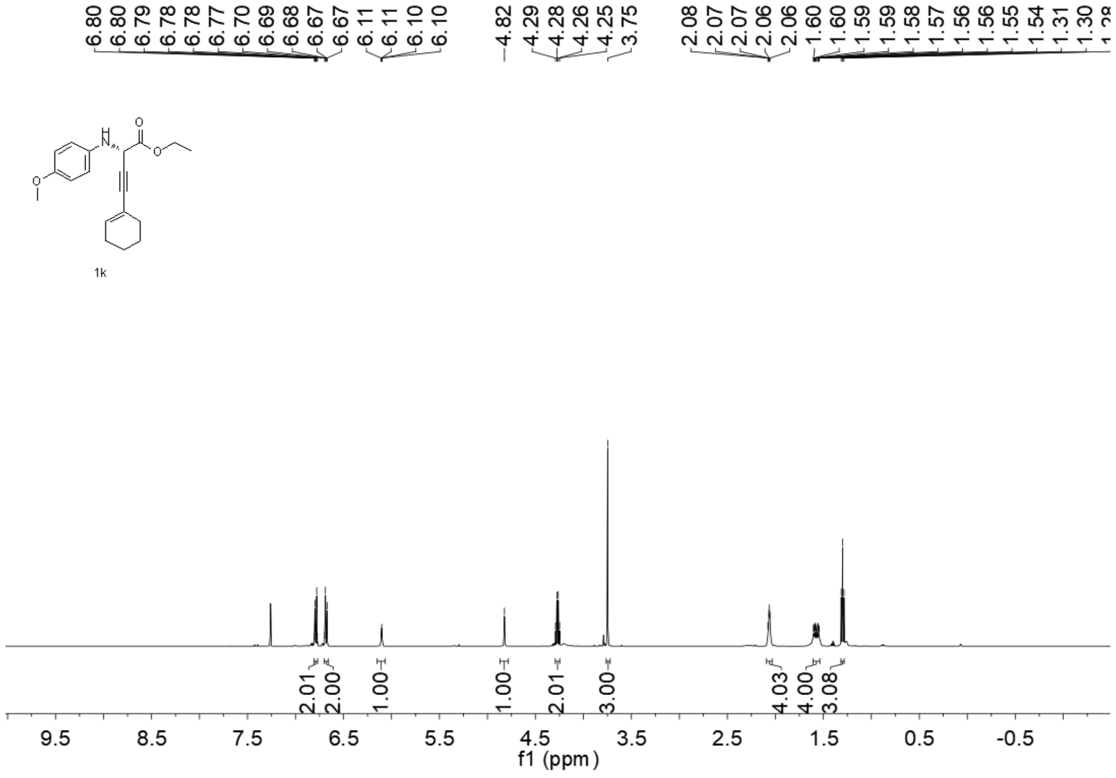
6.80
6.80
6.79
6.78
6.78
6.77
6.70
6.69
6.68
6.67
6.67
6.11
6.11
6.10
6.10
4.82
4.29
4.28
4.26
4.25
3.75
2.08
2.07
2.07
2.06
2.06
1.60
1.60
1.59
1.59
1.58
1.57
1.56
1.56
1.55
1.54
1.31
1.30
1k
2.01
2.00
1.00
1.00
2.01
3.00
4.03
4.00
3.08
9.5
8.5
7.5
6.5
5.5
4.5
3.5
2.5
1.5
0.5
-0.5
f1 (ppm)

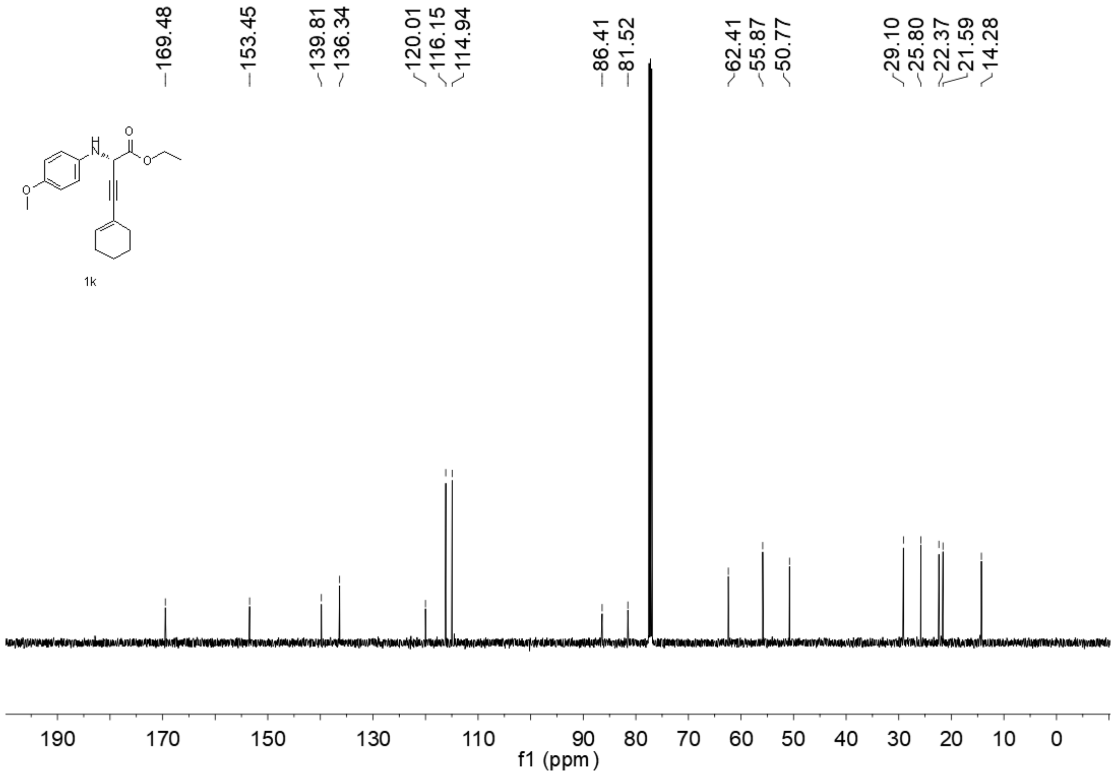
169.48
153.45
139.81
136.34
120.01
116.15
114.94
86.41
81.52
62.41
55.87
50.77
29.10
25.80
22.37
21.59
14.28
1k
190
170
150
130
110
90
80
70
60
50
40
30
20
10
0
f1 (ppm)

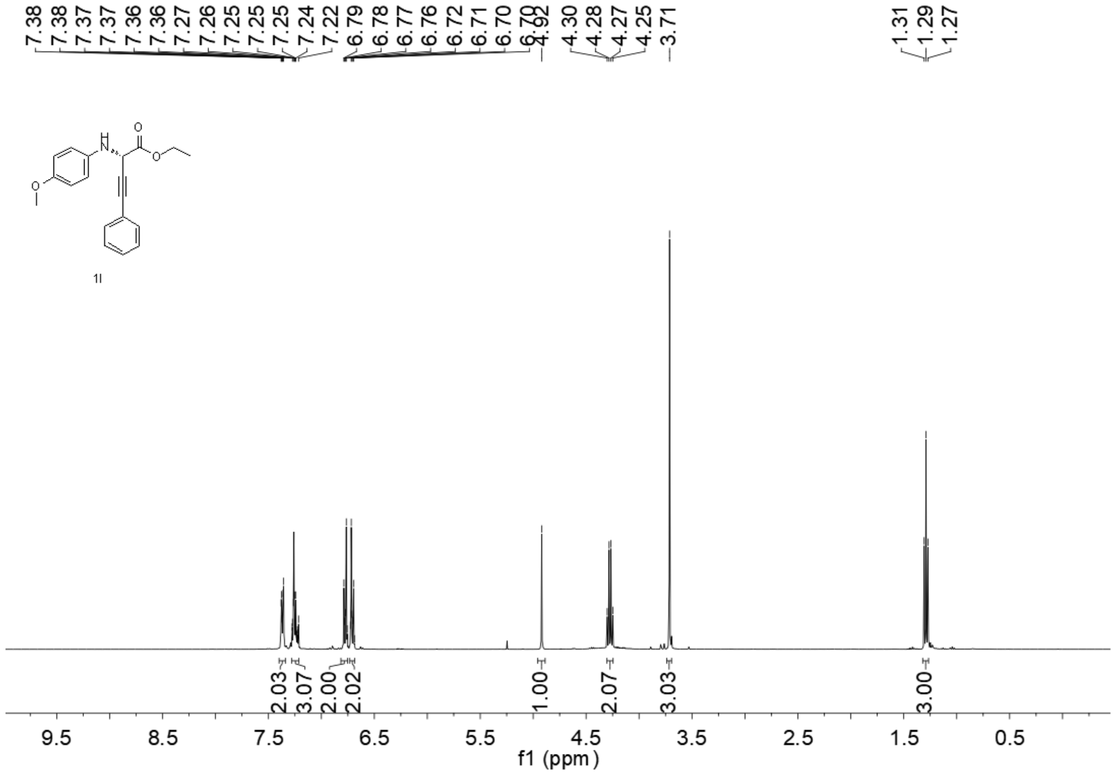
7.38
7.38
7.37
7.37
7.36
7.36
7.27
7.26
7.25
7.25
7.25
7.24
7.22
6.79
6.78
6.77
6.76
6.72
6.71
6.70
6.70
4.92
4.30
4.28
4.27
4.25
3.71
1.31
1.29
1.27
1l
2.03
3.07
2.00
2.02
1.00
2.07
3.03
3.00
f1 (ppm)

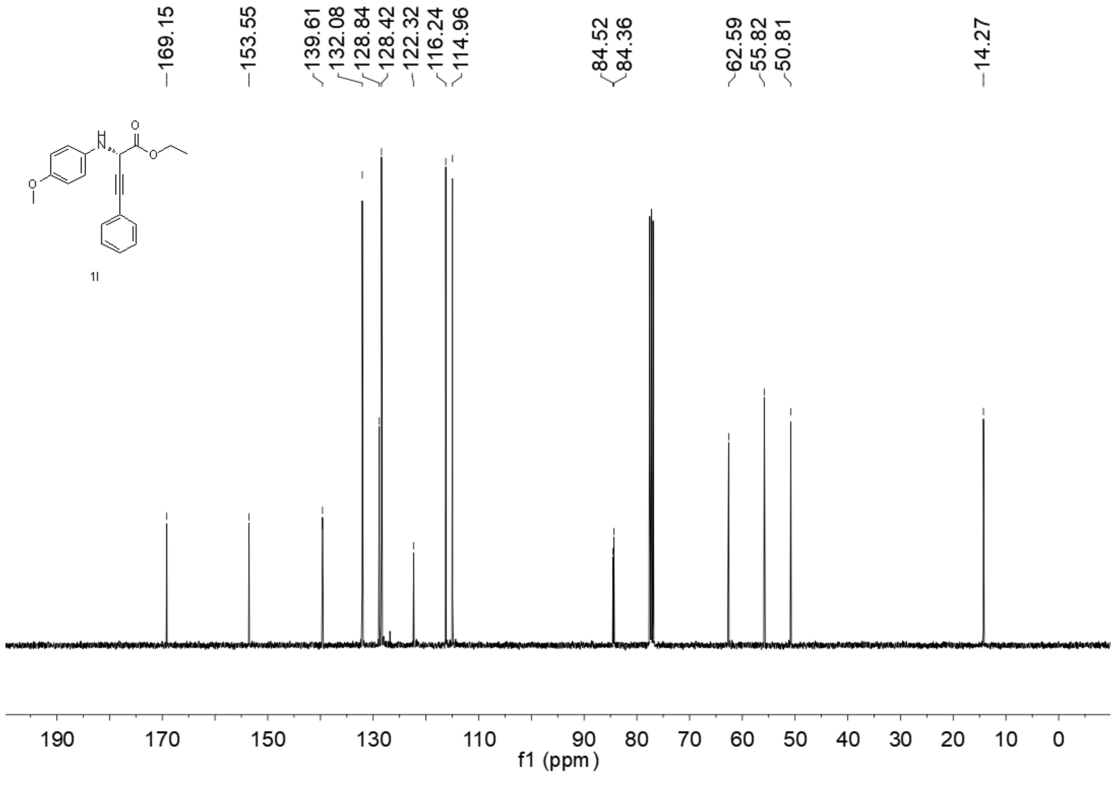
169.15
153.55
139.61
132.08
128.84
128.42
122.32
116.24
114.96
84.52
84.36
62.59
55.82
50.81
14.27
1l
f1 (ppm)

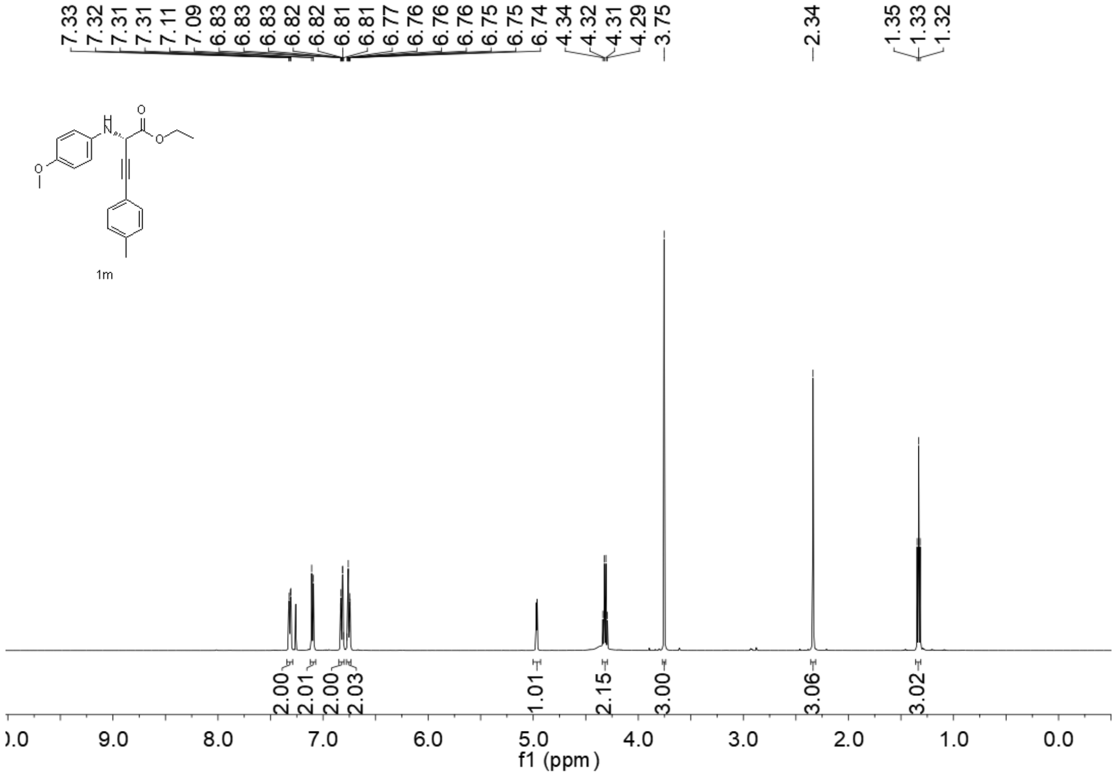
7.33 7.32 7.31 7.31 7.11 7.09 6.83 6.83 6.83 6.82 6.82 6.81 6.81 6.77 6.76 6.76 6.76 6.75 6.75 6.74 4.34 4.32 4.31 4.29 3.75 2.34 1.35 1.33 1.32
1m
2.00 2.01 2.00 2.03 1.01 2.15 3.00 3.06 3.02
f1 (ppm)

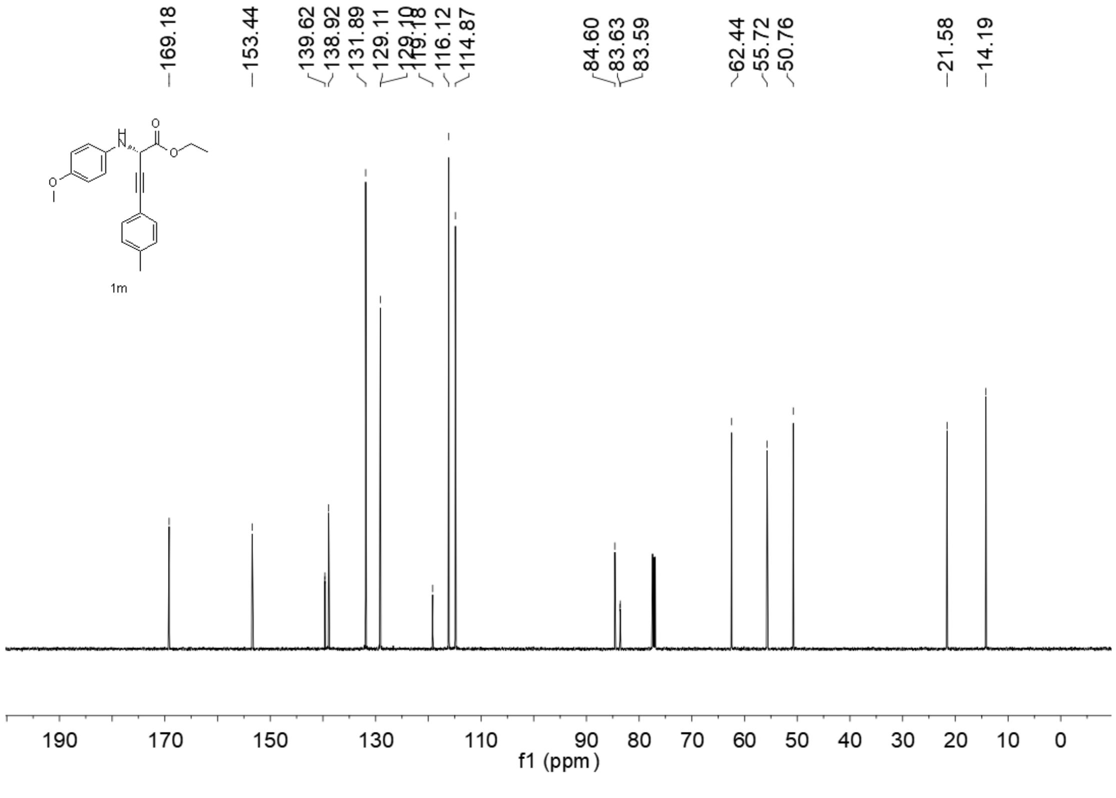
169.18 153.44 139.62 138.92 131.89 129.11 129.10 119.18 116.12 114.87 84.60 83.63 83.59 62.44 55.72 50.76 21.58 14.19
1m
190 170 150 130 110 90 80 70 60 50 40 30 20 10 0
f1 (ppm)

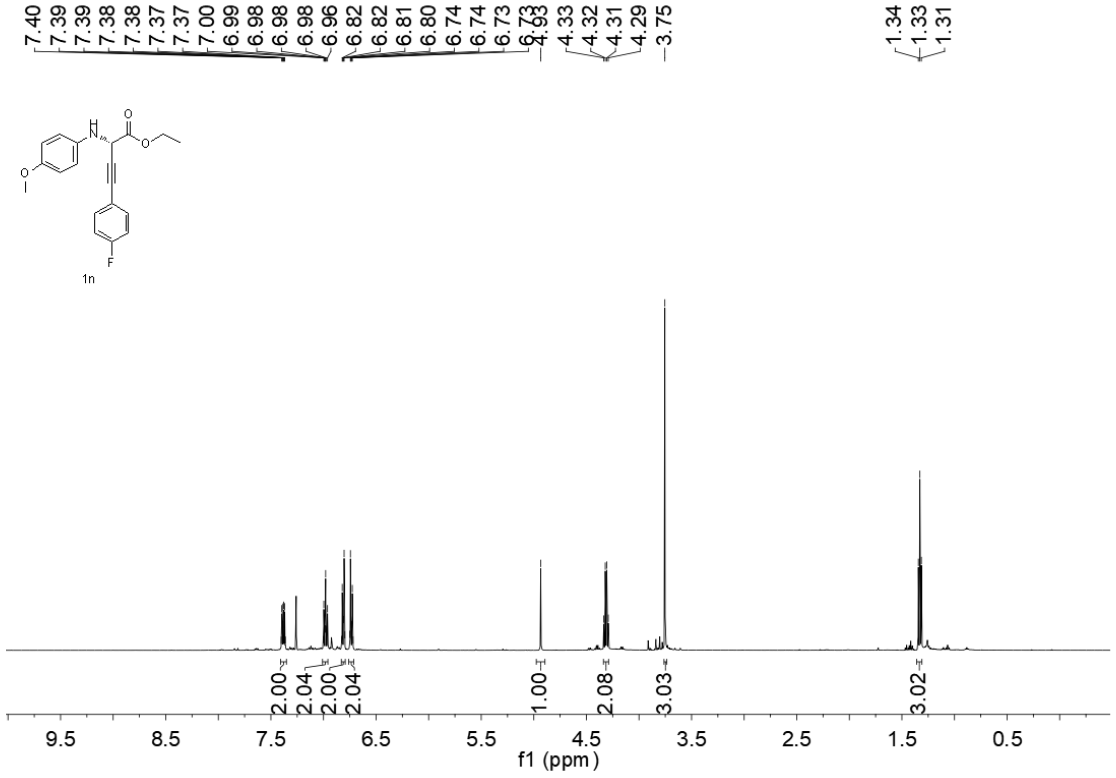
7.40
7.39
7.39
7.38
7.38
7.37
7.37
7.00
6.99
6.98
6.98
6.96
6.82
6.82
6.81
6.80
6.74
6.74
6.73
6.73
4.93
4.33
4.32
4.31
4.29
3.75
1.34
1.33
1.31
1n
2.00
2.04
2.00
2.04
1.00
2.08
3.03
3.02
9.5
8.5
7.5
6.5
5.5
4.5
3.5
2.5
1.5
0.5
f1 (ppm)

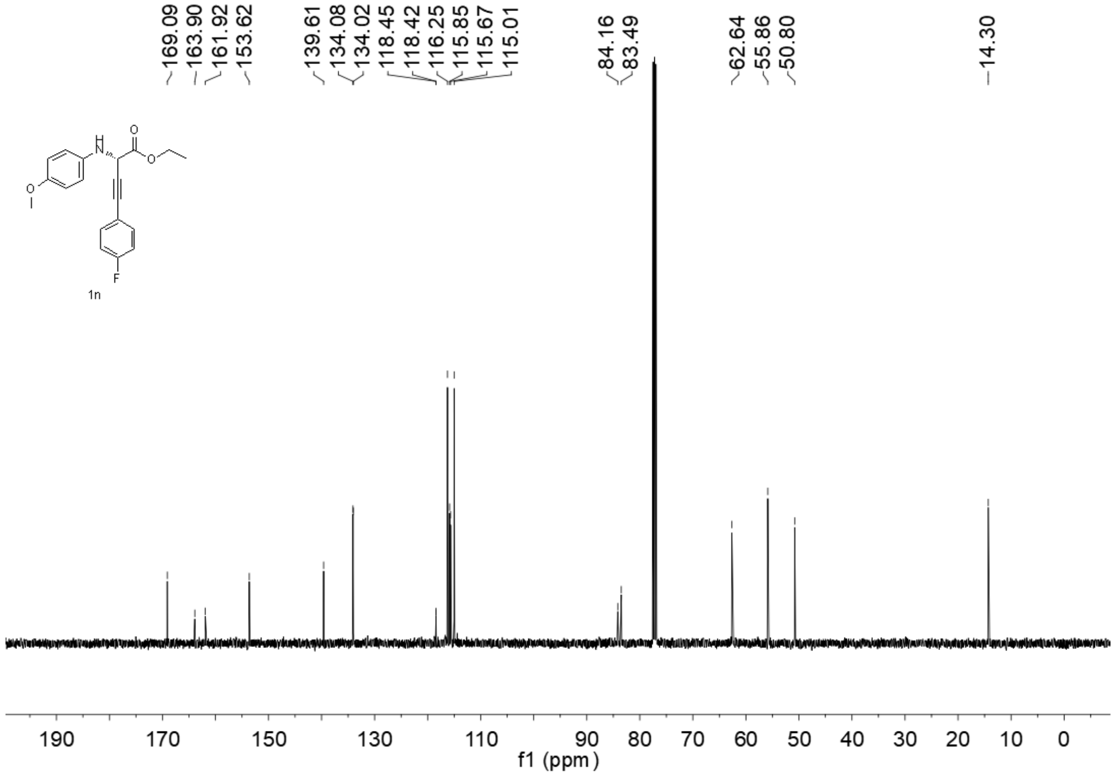
169.09
163.90
161.92
153.62
139.61
134.08
134.02
118.45
118.42
116.25
115.85
115.67
115.01
84.16
83.49
62.64
55.86
50.80
14.30
1n
190
170
150
130
110
90
80
70
60
50
40
30
20
10
0
f1 (ppm)

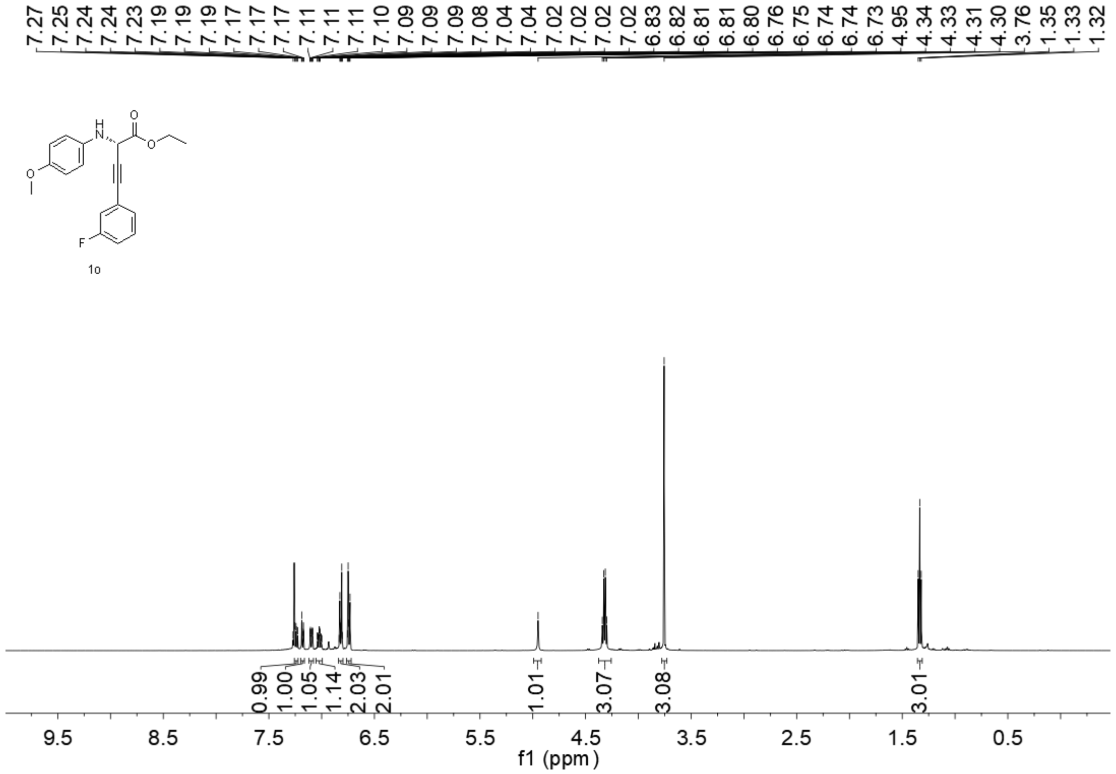
7.27 7.25 7.24 7.24 7.23 7.19 7.19 7.19 7.17 7.17 7.17 7.11 7.11 7.11 7.10 7.09 7.09 7.09 7.08 7.04 7.04 7.02 7.02 7.02 7.02 6.83 6.82 6.81 6.81 6.80 6.76 6.75 6.74 6.74 6.73 4.95 4.34 4.33 4.31 4.30 3.76 1.35 1.33 1.32
1o
0.99 1.00 1.05 1.14 2.03 2.01
1.01 3.07 3.08 3.01
9.5 8.5 7.5 6.5 5.5 4.5 3.5 2.5 1.5 0.5
f1 (ppm)

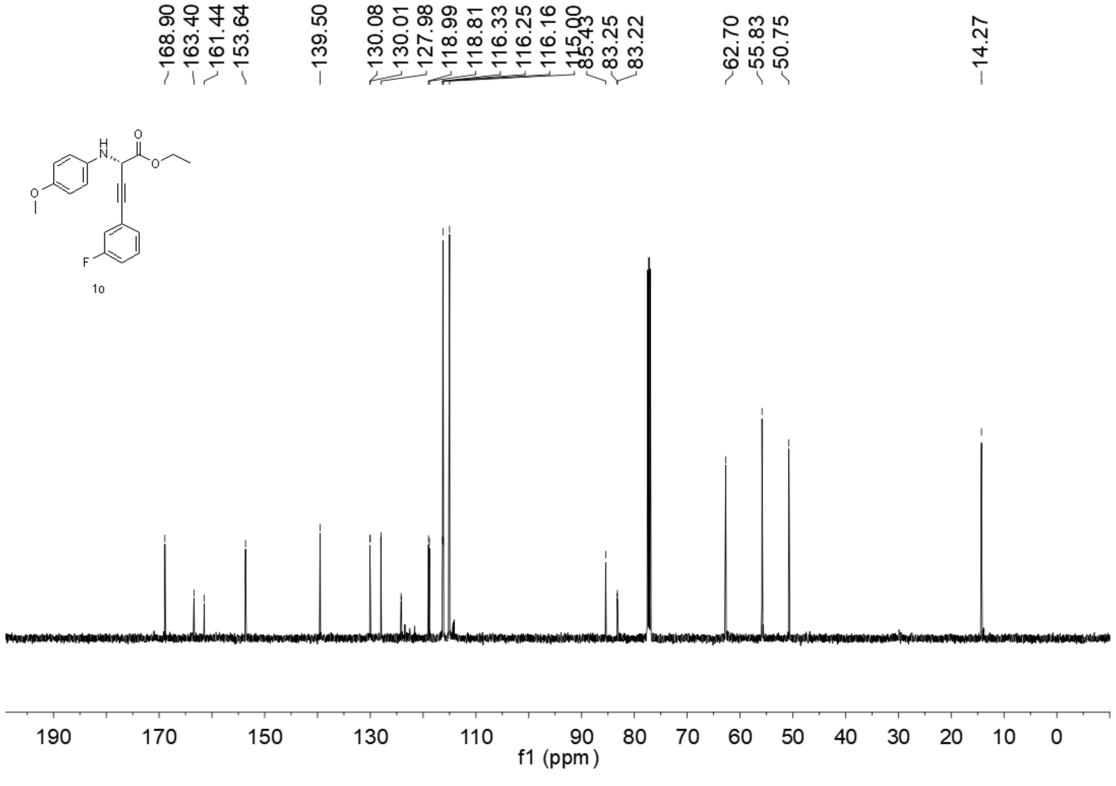
168.90 163.40 161.44 153.64 139.50 130.08 130.01 127.98 118.99 118.81 116.33 116.25 116.16 115.00 85.43 83.25 83.22 62.70 55.83 50.75 14.27
1o
190 170 150 130 110 90 80 70 60 50 40 30 20 10 0
f1 (ppm)

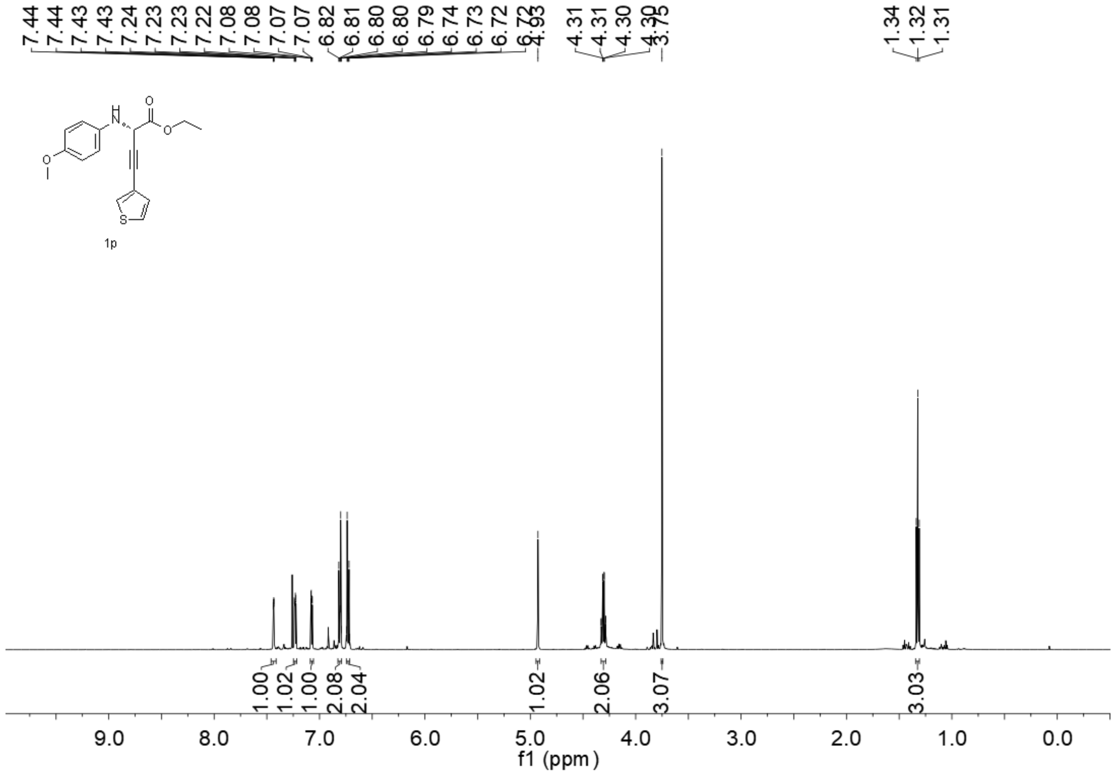
7.44
7.44
7.43
7.43
7.24
7.23
7.23
7.22
7.08
7.08
7.07
7.07
6.82
6.81
6.80
6.80
6.79
6.74
6.73
6.72
6.72
4.93
4.31
4.31
4.30
4.30
3.75
1.34
1.32
1.31
1p
1.00
1.02
1.00
2.08
2.04
1.02
2.06
3.07
3.03
9.0
8.0
7.0
6.0
5.0
4.0
3.0
2.0
1.0
0.0
f1 (ppm)

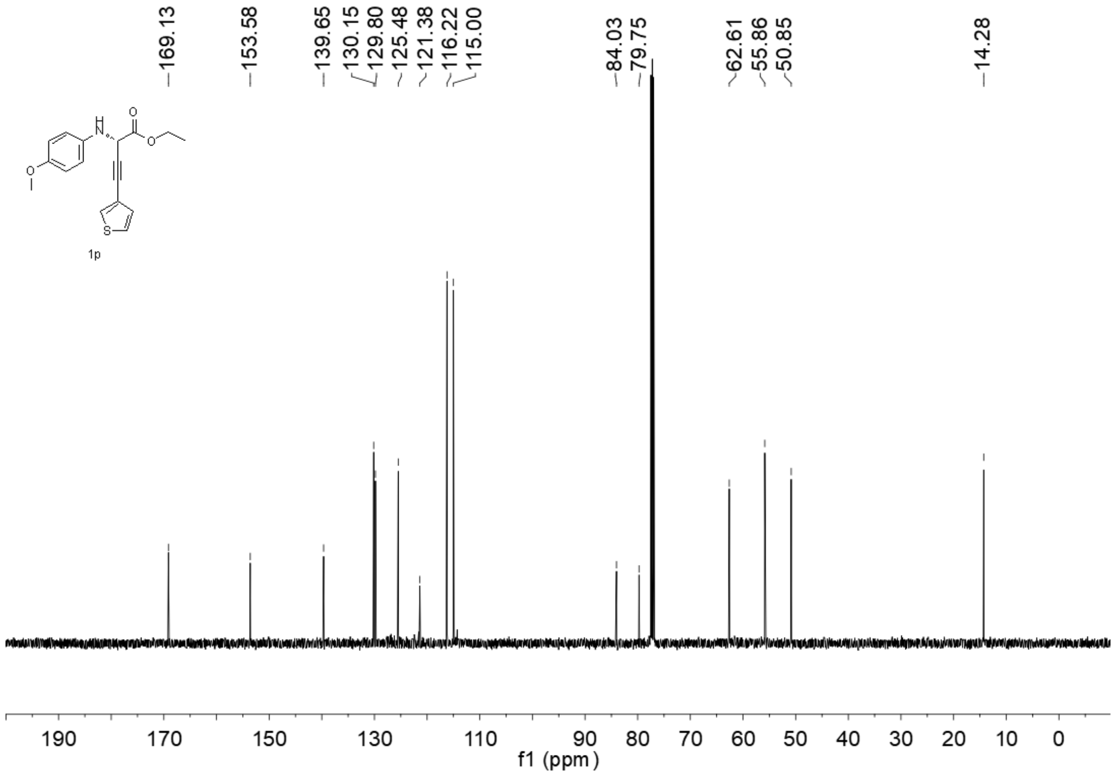
169.13
153.58
139.65
130.15
129.80
125.48
121.38
116.22
115.00
84.03
79.75
62.61
55.86
50.85
14.28
1p
190
170
150
130
110
90
80
70
60
50
40
30
20
10
0
f1 (ppm)

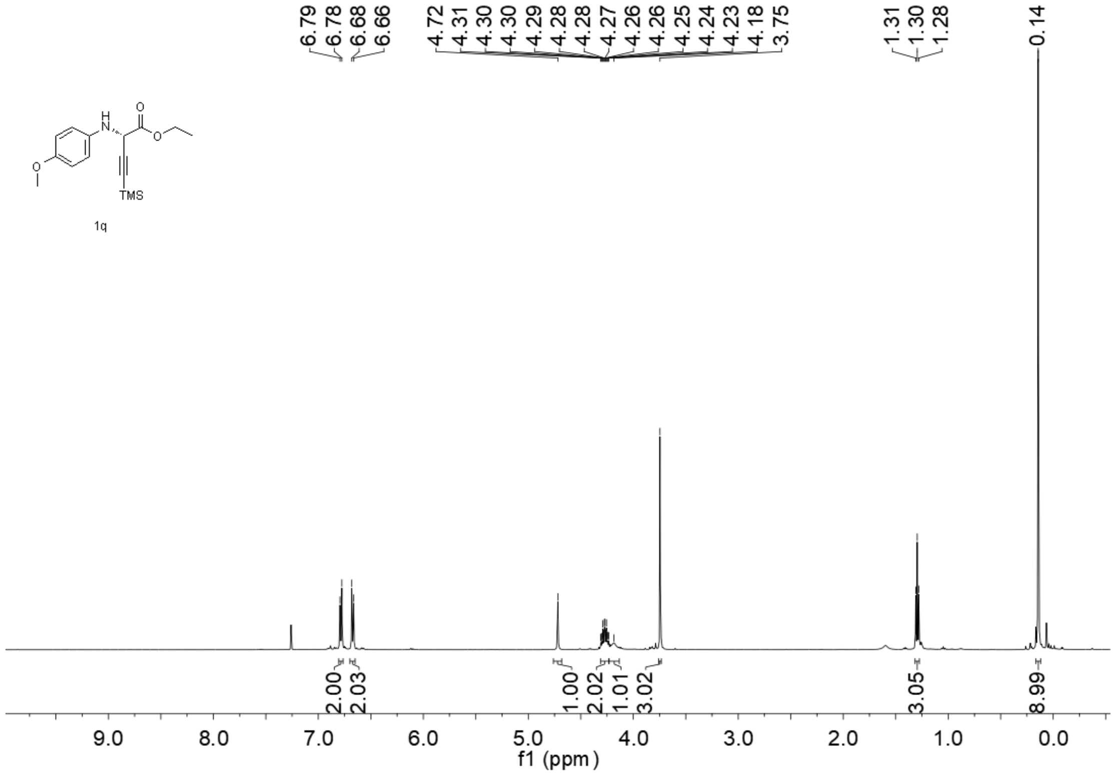
6.79
6.78
6.68
6.66
4.72
4.31
4.30
4.30
4.29
4.28
4.28
4.27
4.26
4.26
4.25
4.24
4.23
4.18
3.75
1.31
1.30
1.28
0.14
TMS
1q
2.00
2.03
1.00
2.02
1.01
3.02
3.05
8.99
9.0
8.0
7.0
6.0
5.0
4.0
3.0
2.0
1.0
0.0
f1 (ppm)

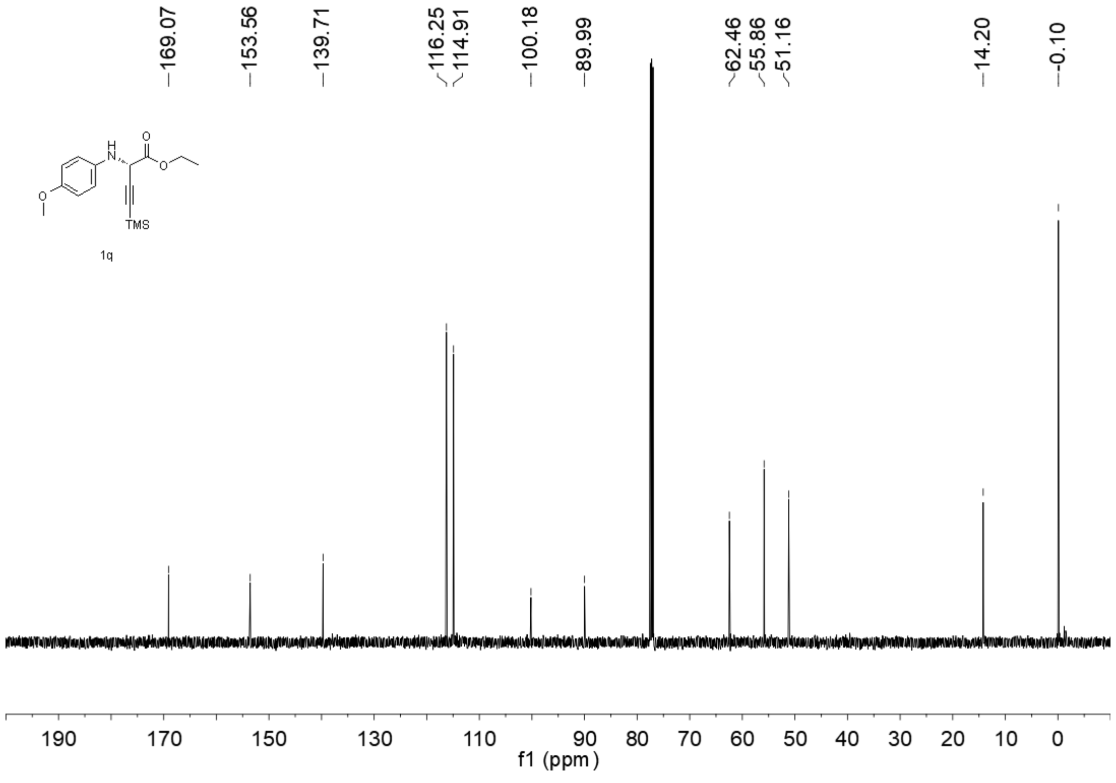
169.07
153.56
139.71
116.25
114.91
100.18
89.99
62.46
55.86
51.16
14.20
-0.10
TMS
1q
190
170
150
130
110
90
80
70
60
50
40
30
20
10
0
f1 (ppm)

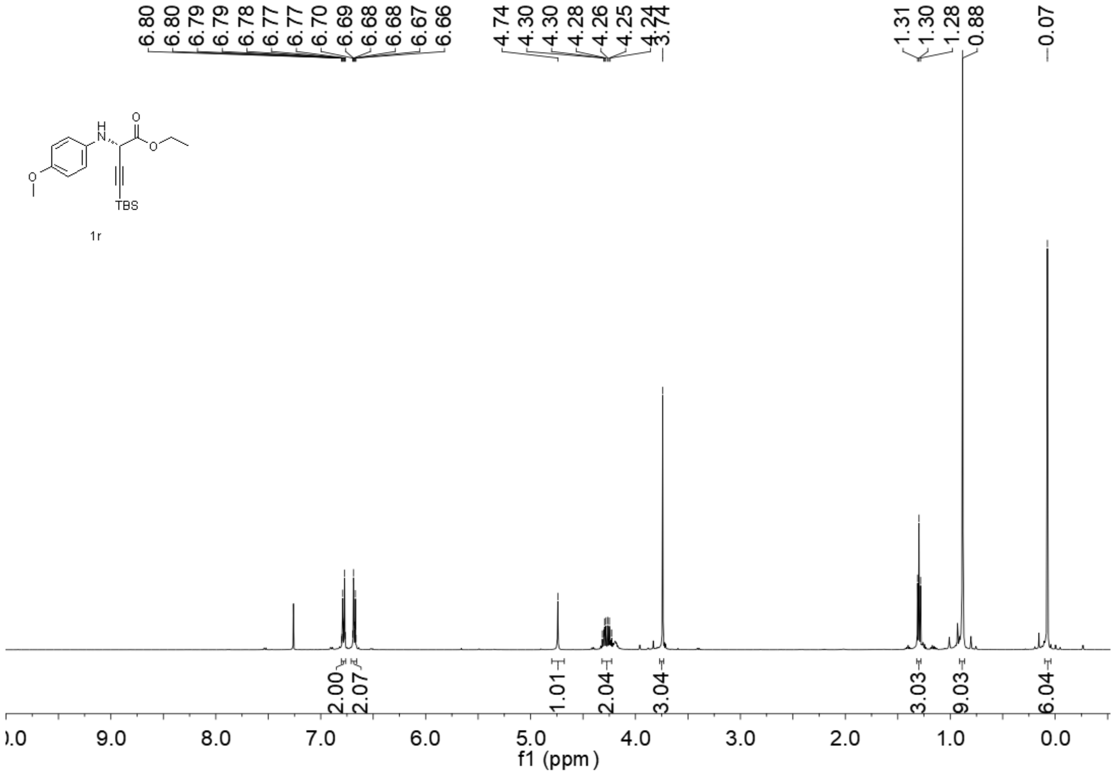
6.80 6.80 6.79 6.79 6.78 6.77 6.77 6.70 6.69 6.68 6.68 6.67 6.66
4.74 4.30 4.30 4.28 4.26 4.25 4.24 3.74
1.31 1.30 1.28 0.88
0.07
TBS
1r
2.00 2.07 1.01 2.04 3.04 3.03 9.03 6.04
f1 (ppm)

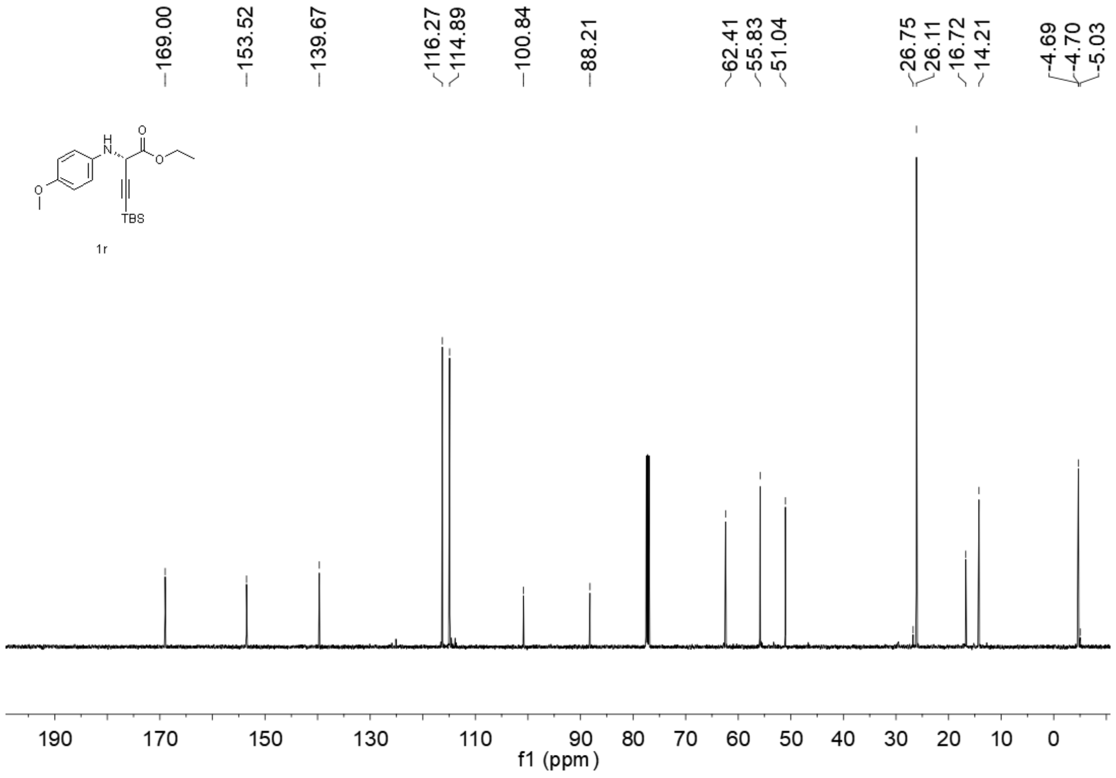
169.00
153.52
139.67
116.27 114.89
100.84
88.21
62.41 55.83 51.04
26.75 26.11 16.72 14.21
-4.69 -4.70 -5.03
TBS
1r
f1 (ppm)

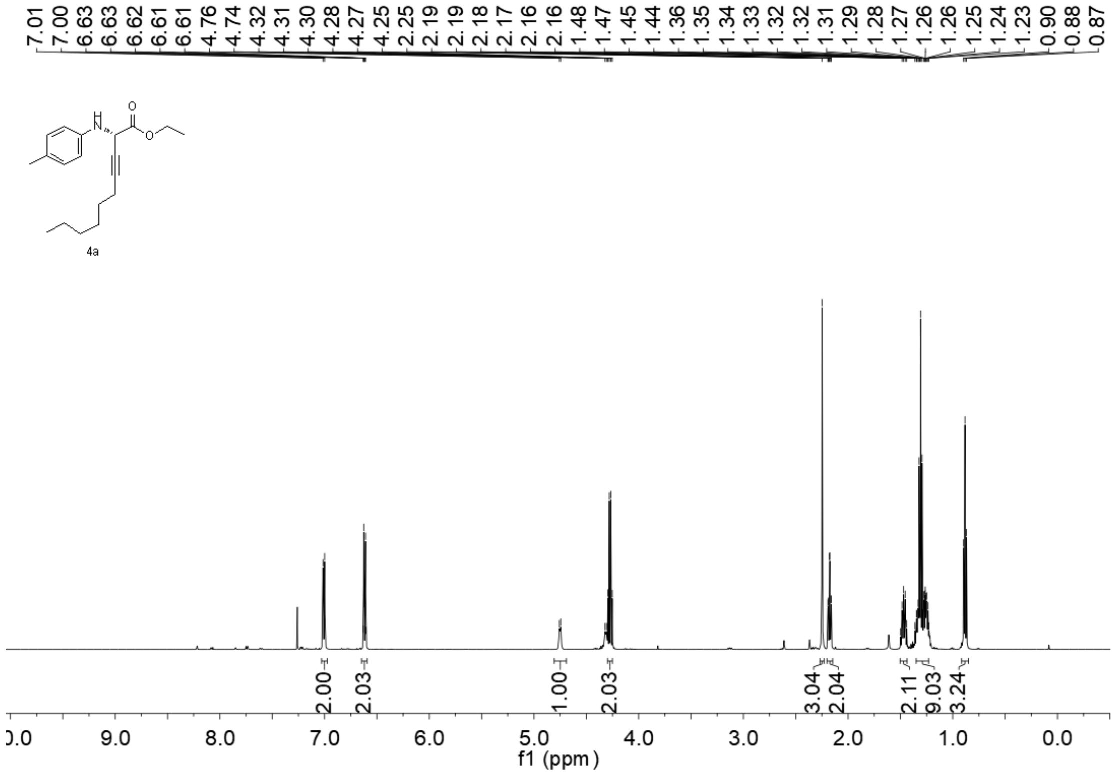
7.01 7.00 6.63 6.63 6.62 6.61 6.61 4.76 4.74 4.32 4.31 4.30 4.28 4.27 4.25 2.25 2.19 2.19 2.18 2.17 2.16 2.16 1.48 1.47 1.45 1.44 1.36 1.35 1.34 1.33 1.32 1.32 1.31 1.29 1.28 1.27 1.26 1.26 1.25 1.24 1.23 0.90 0.88 0.87
4a
2.00 2.03 1.00 2.03 3.04 2.04 2.11 9.03 3.24
9.0 8.0 7.0 6.0 5.0 4.0 3.0 2.0 1.0 0.0
f1 (ppm)

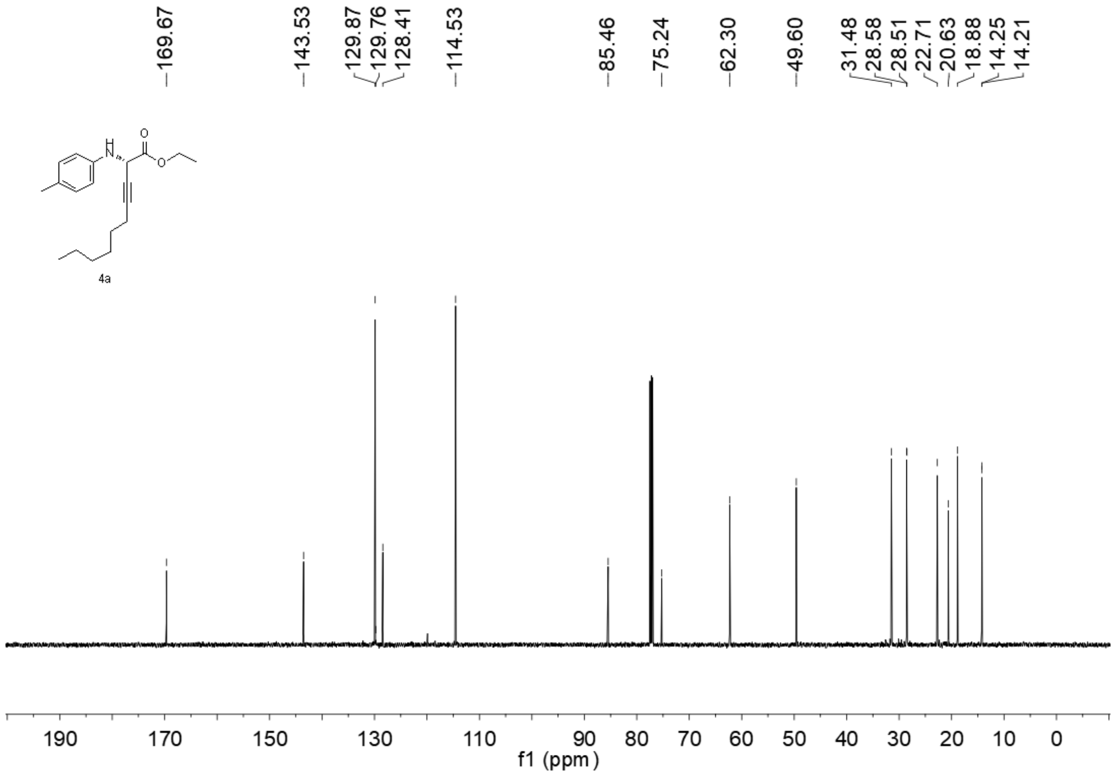
169.67 143.53 129.87 129.76 128.41 114.53 85.46 75.24 62.30 49.60 31.48 28.58 28.51 22.71 20.63 18.88 14.25 14.21
4a
190 170 150 130 110 90 80 70 60 50 40 30 20 10 0
f1 (ppm)

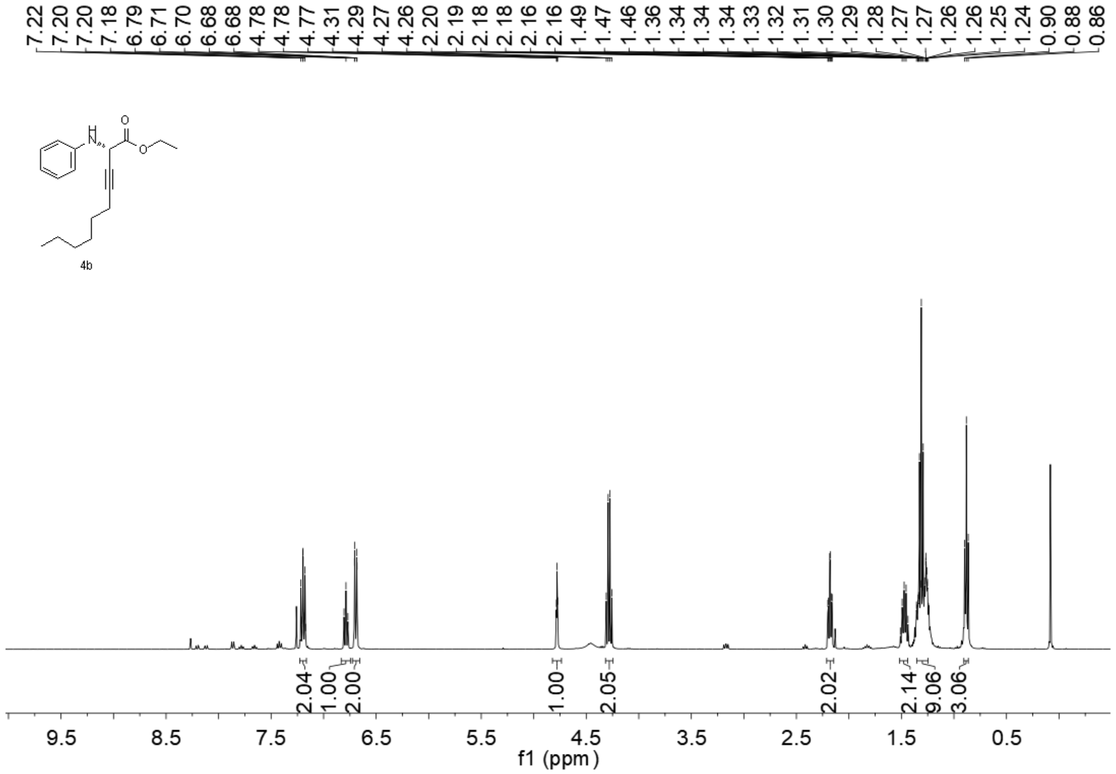

7.22
7.20
7.20
7.18
6.79
6.71
6.70
6.68
6.68
4.78
4.78
4.77
4.31
4.29
4.27
4.26
2.20
2.19
2.18
2.18
2.16
2.16
1.49
1.47
1.46
1.36
1.34
1.34
1.34
1.33
1.32
1.31
1.30
1.29
1.28
1.27
1.27
1.27
1.26
1.26
1.25
1.24
0.90
0.88
0.86
4b
2.04
1.00
2.00
1.00
2.05
2.02
2.14
9.06
3.06
9.5
8.5
7.5
6.5
5.5
4.5
3.5
2.5
1.5
0.5
f1 (ppm)

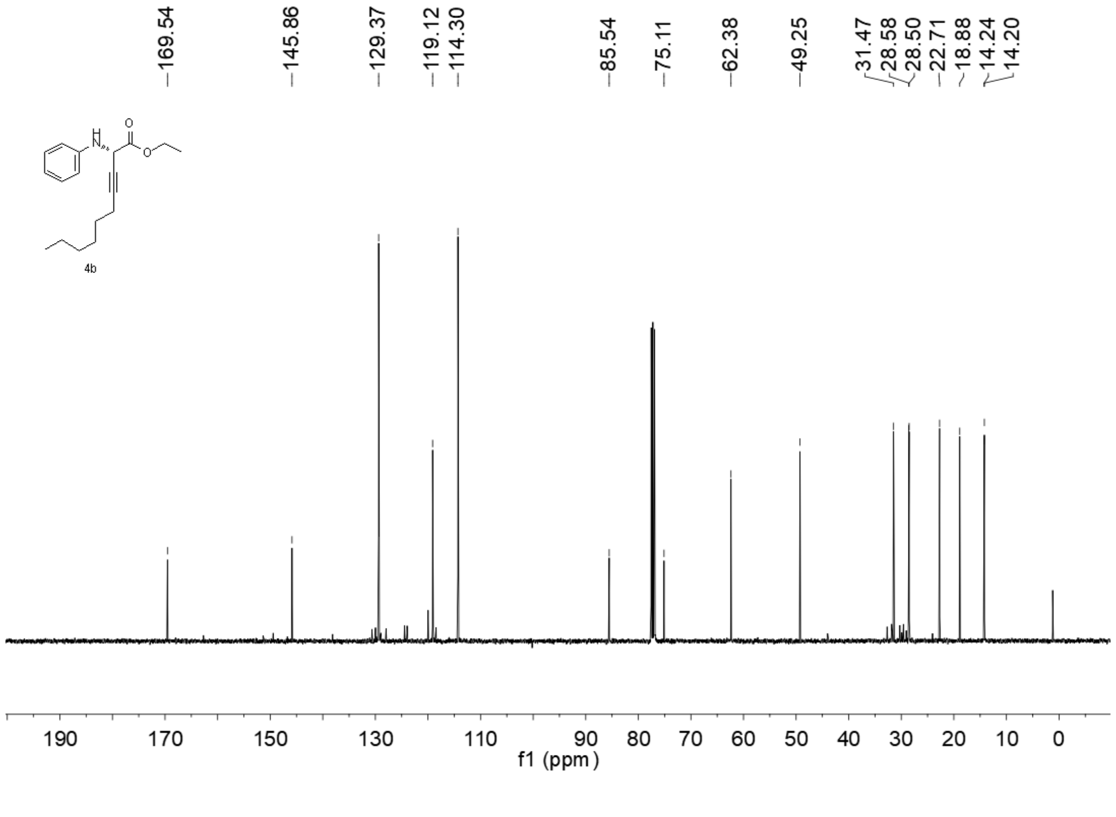

169.54
145.86
129.37
119.12
114.30
85.54
75.11
62.38
49.25
31.47
28.58
28.50
22.71
18.88
14.24
14.20
4b
190
170
150
130
110
90
80
70
60
50
40
30
20
10
0
f1 (ppm)

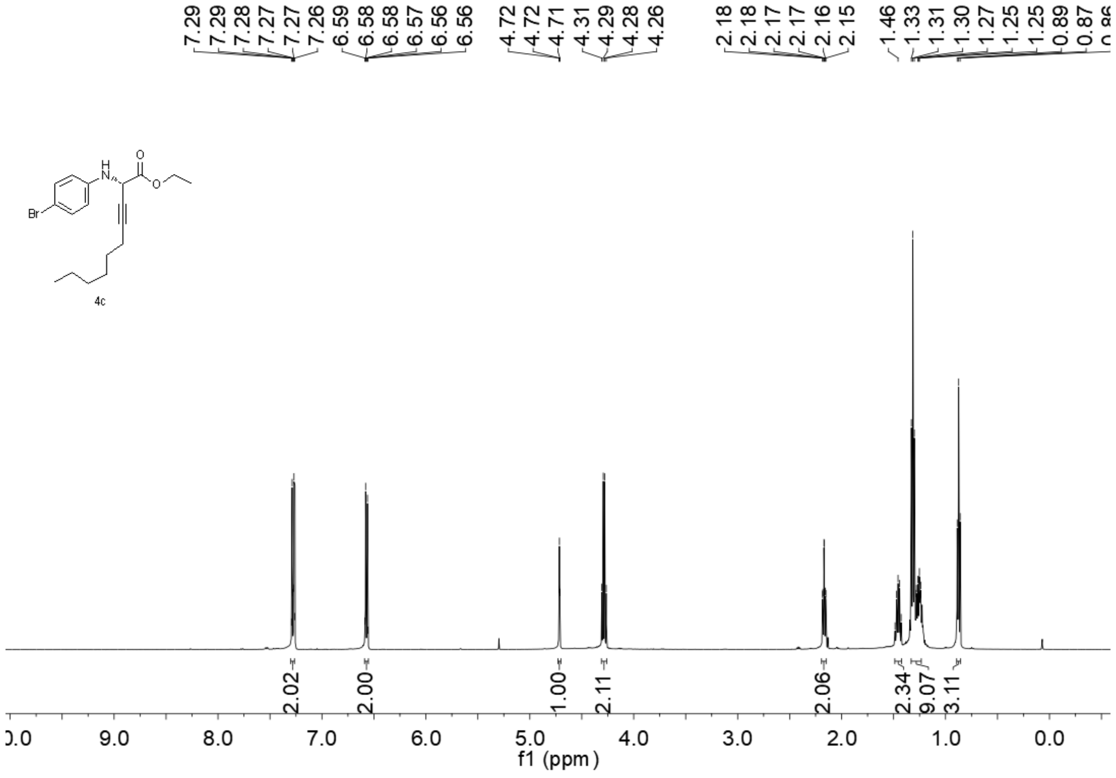
7.29
7.29
7.28
7.27
7.27
7.26
6.59
6.58
6.58
6.57
6.56
6.56
4.72
4.72
4.71
4.31
4.29
4.28
4.26
2.18
2.18
2.17
2.17
2.16
2.15
1.46
1.33
1.31
1.30
1.27
1.25
1.25
0.89
0.87
Br
N
H
O
O
4c
2.02
2.00
1.00
2.11
2.06
2.34
9.07
3.11
9.0
8.0
7.0
6.0
5.0
4.0
3.0
2.0
1.0
0.0
f1 (ppm)

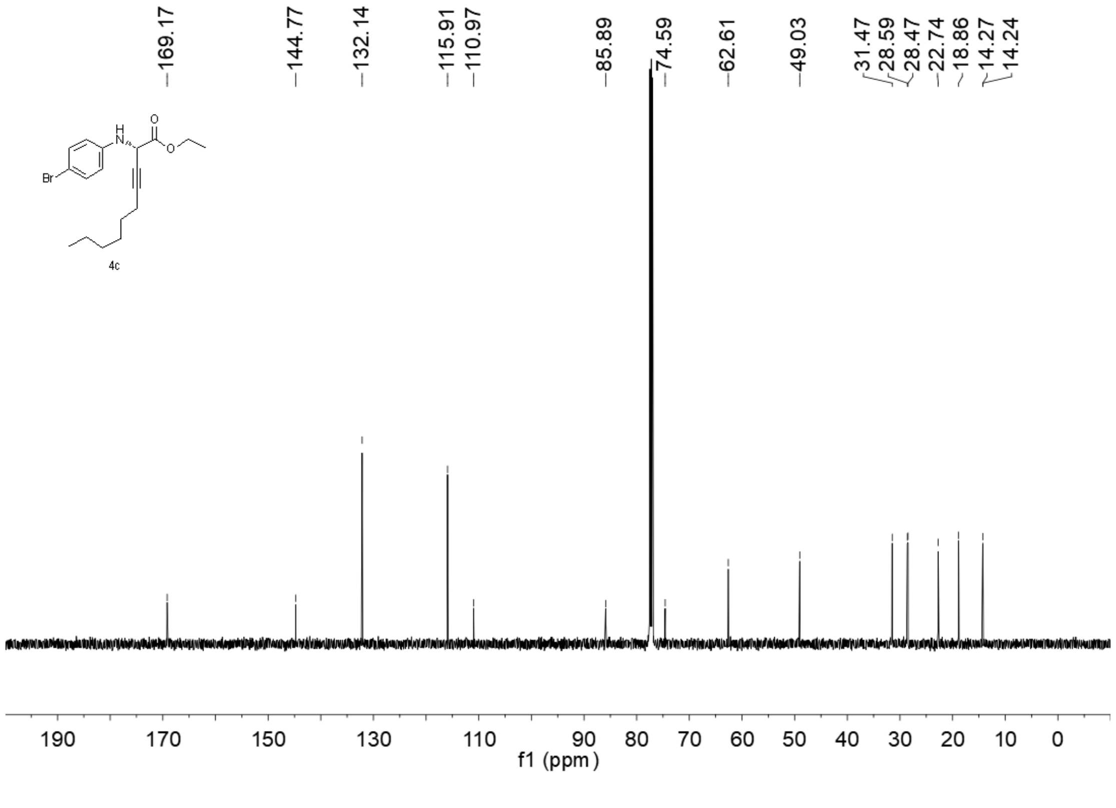
169.17
144.77
132.14
115.91
110.97
85.89
74.59
62.61
49.03
31.47
28.59
28.47
22.74
18.86
14.27
14.24
Br
N
H
O
O
4c
190
170
150
130
110
90
80
70
60
50
40
30
20
10
0
f1 (ppm)

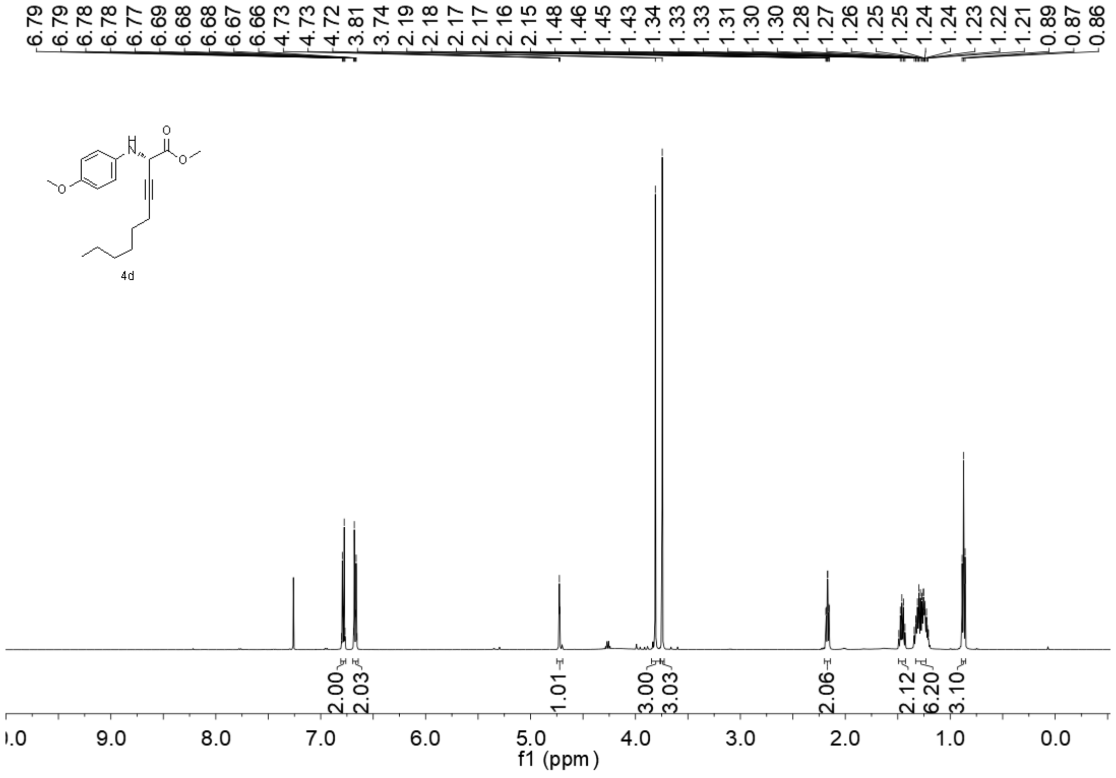

6.79
6.79
6.78
6.78
6.77
6.69
6.68
6.68
6.67
6.66
4.73
4.73
4.72
3.81
3.74
2.19
2.18
2.17
2.17
2.16
2.15
1.48
1.46
1.45
1.43
1.34
1.33
1.33
1.31
1.30
1.30
1.28
1.27
1.26
1.25
1.25
1.24
1.24
1.23
1.22
1.21
0.89
0.87
0.86
4d
2.00
2.03
1.01
3.00
3.03
2.06
2.12
6.20
3.10
9.0
8.0
7.0
6.0
5.0
4.0
3.0
2.0
1.0
0.0
f1 (ppm)

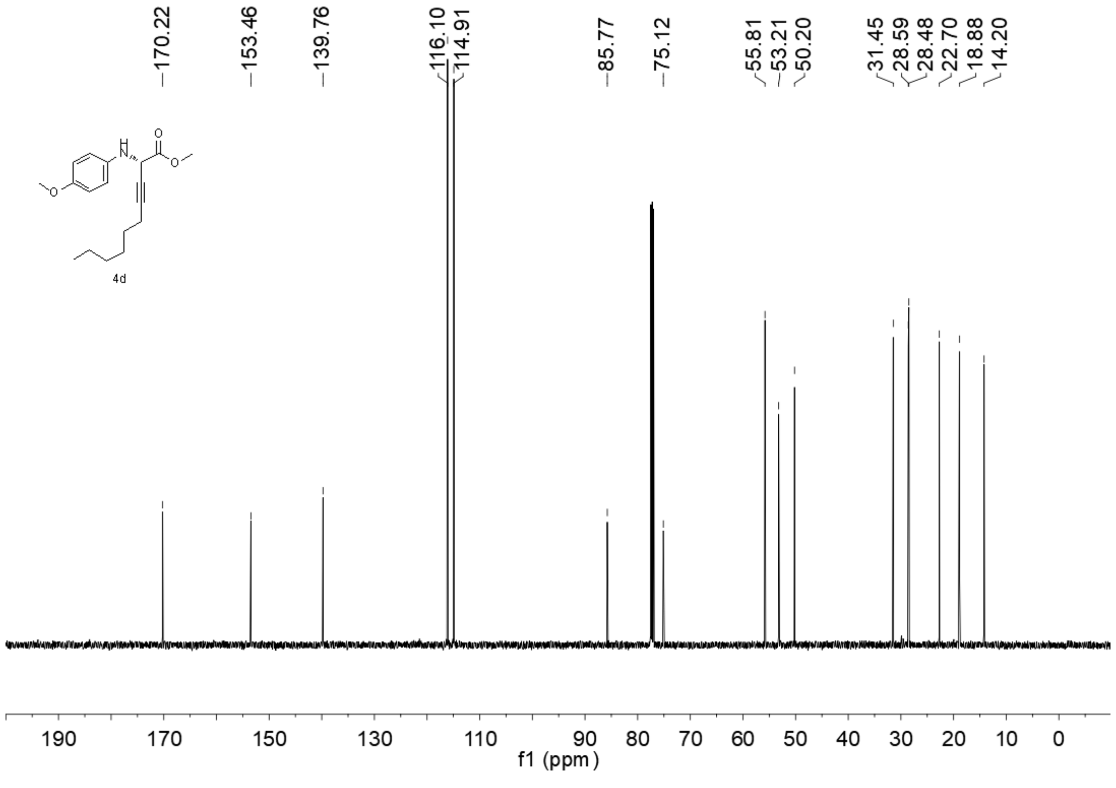

170.22
153.46
139.76
116.10
114.91
85.77
75.12
55.81
53.21
50.20
31.45
28.59
28.48
22.70
18.88
14.20
4d
190
170
150
130
110
90
80
70
60
50
40
30
20
10
0
f1 (ppm)

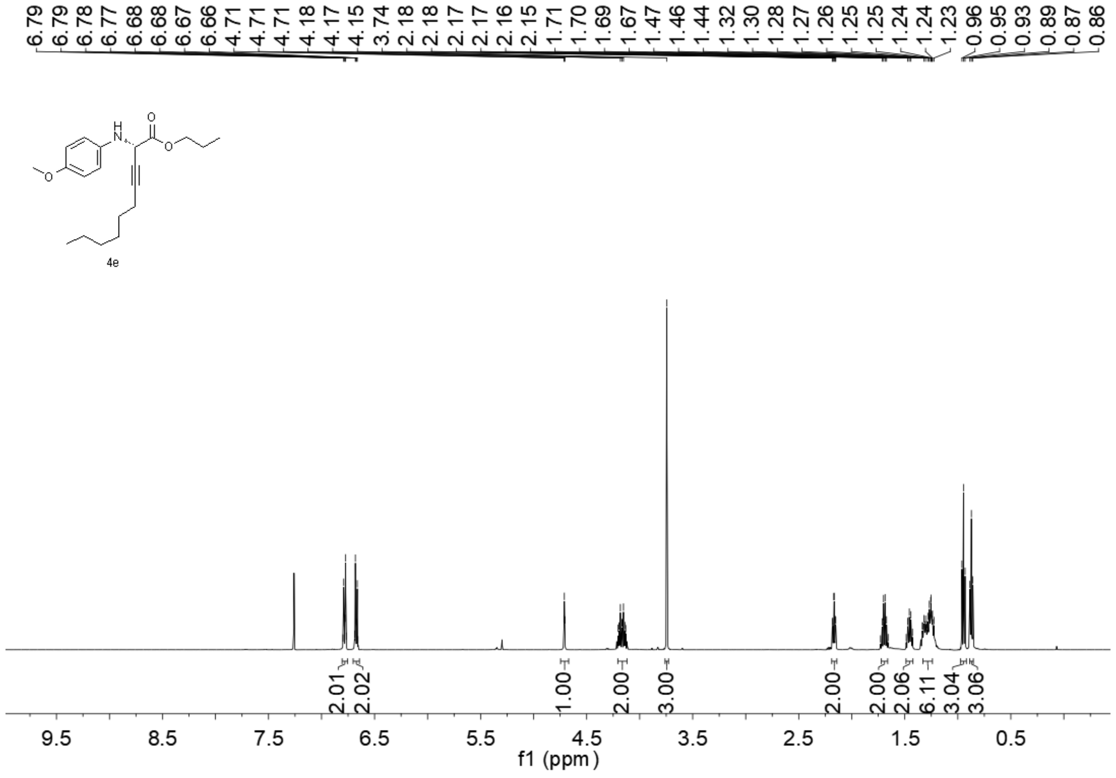
6.79 6.79 6.78 6.77 6.68 6.68 6.67 6.66 4.71 4.71 4.71 4.18 4.17 4.15 3.74 2.18 2.18 2.17 2.17 2.16 2.15 1.71 1.70 1.69 1.67 1.47 1.46 1.44 1.32 1.30 1.28 1.27 1.26 1.25 1.25 1.24 1.24 1.23 0.96 0.95 0.93 0.89 0.87 0.86
4e
2.01 2.02 1.00 2.00 3.00 2.00 2.00 2.06 6.11 3.04 3.06
9.5 8.5 7.5 6.5 5.5 4.5 3.5 2.5 1.5 0.5
f1 (ppm)

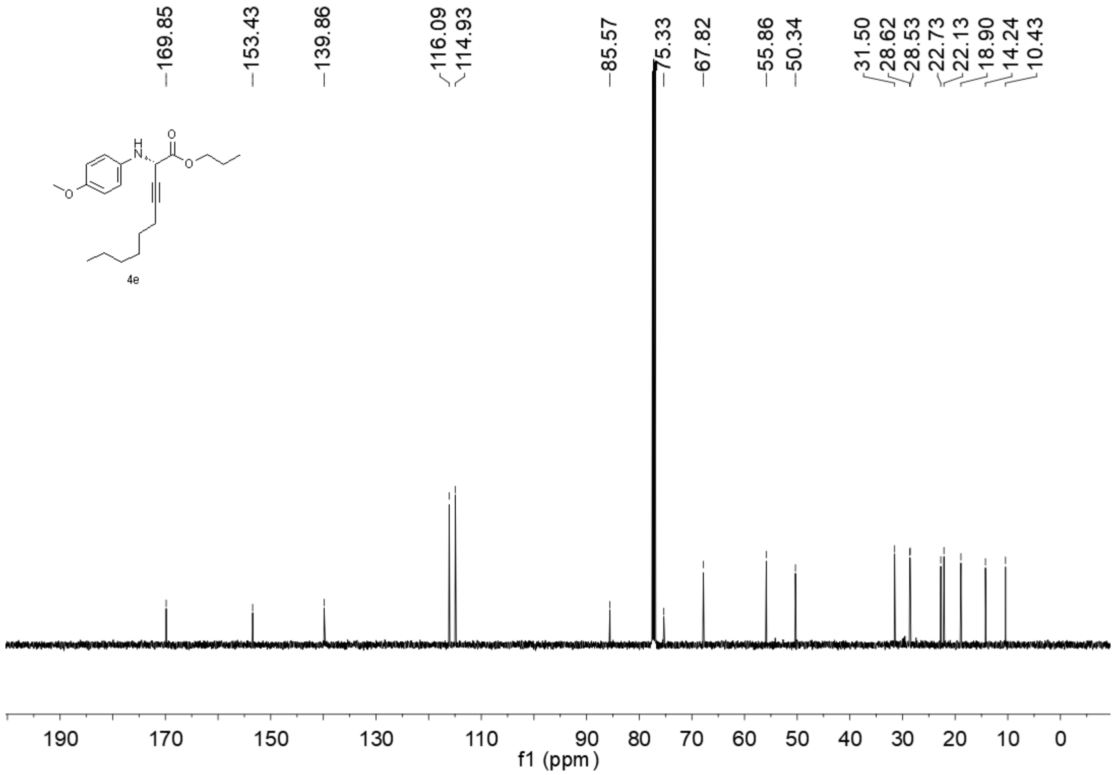
169.85 153.43 139.86 116.09 114.93 85.57 75.33 67.82 55.86 50.34 31.50 28.62 28.53 22.73 22.13 18.90 14.24 10.43
4e
190 170 150 130 110 90 80 70 60 50 40 30 20 10 0
f1 (ppm)

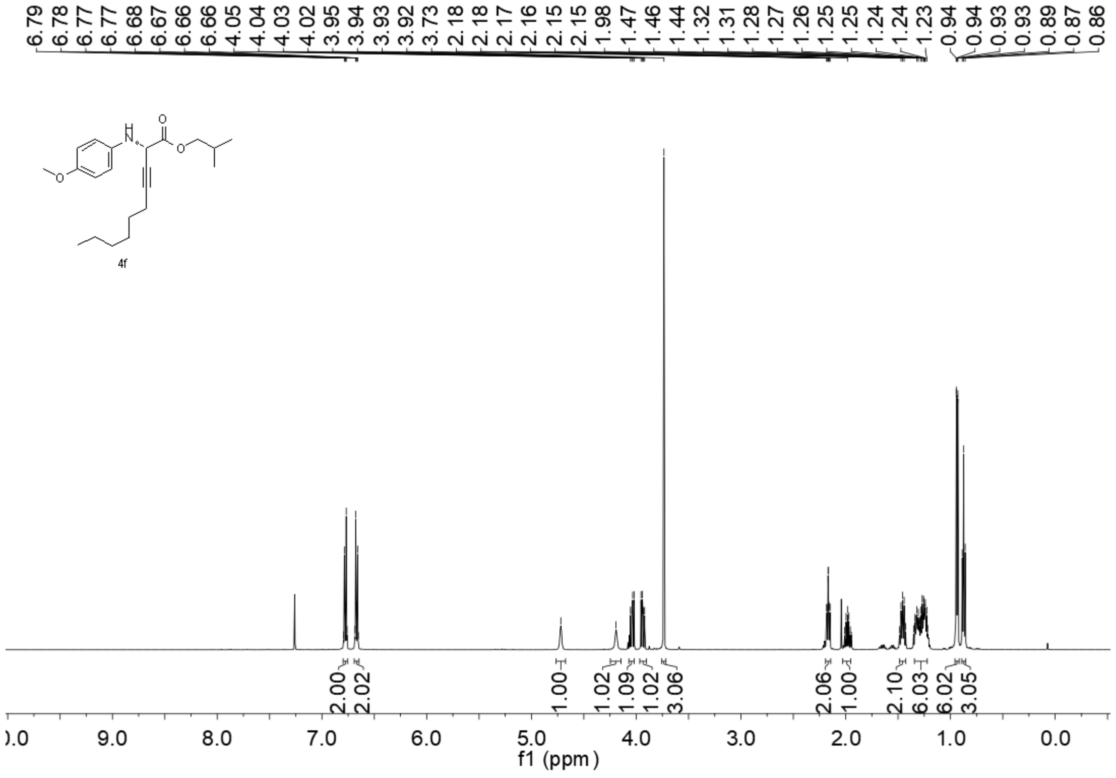
6.79 6.78 6.77 6.77 6.68 6.67 6.66 6.66 4.05 4.04 4.03 4.02 3.95 3.94 3.93 3.92 3.73 2.18 2.18 2.17 2.16 2.15 2.15 1.98 1.47 1.46 1.44 1.32 1.31 1.28 1.27 1.26 1.25 1.25 1.24 1.24 1.23 0.94 0.94 0.93 0.93 0.89 0.87 0.86
4f
2.00 2.02 1.00 1.02 1.09 1.02 3.06 2.06 1.00 2.10 6.03 6.02 3.05
f1 (ppm)

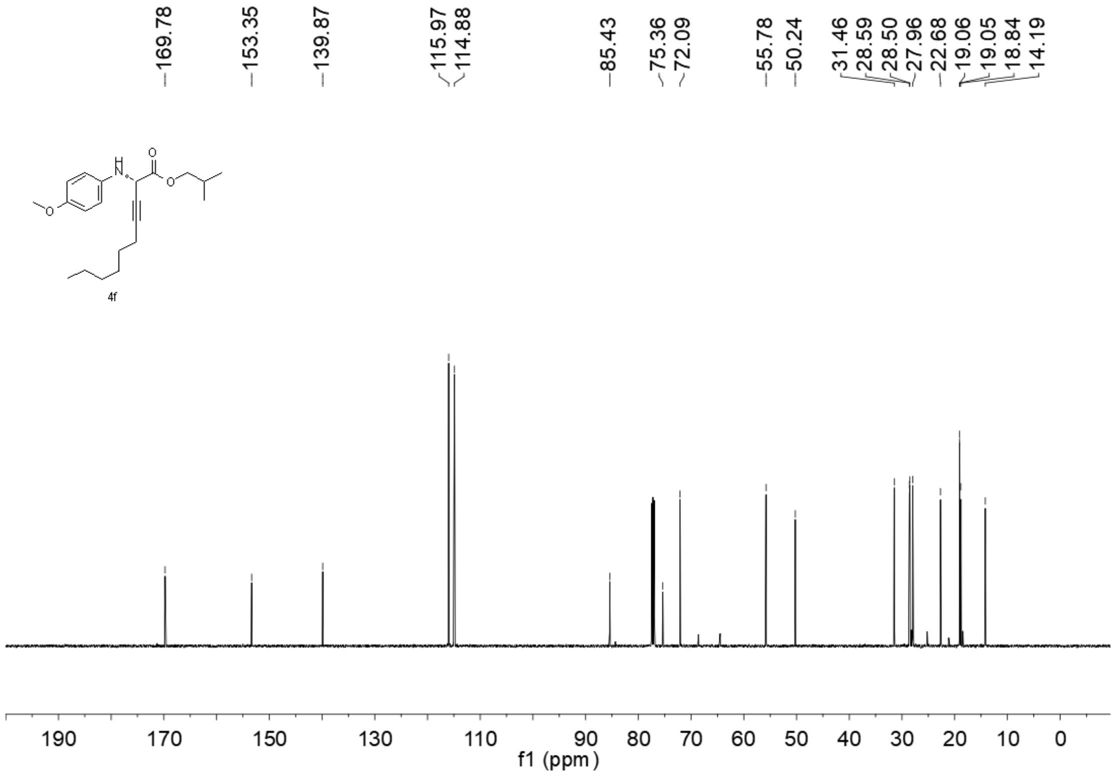
169.78 153.35 139.87 115.97 114.88 85.43 75.36 72.09 55.78 50.24 31.46 28.59 28.50 27.96 22.68 19.06 19.05 18.84 14.19
4f
f1 (ppm)

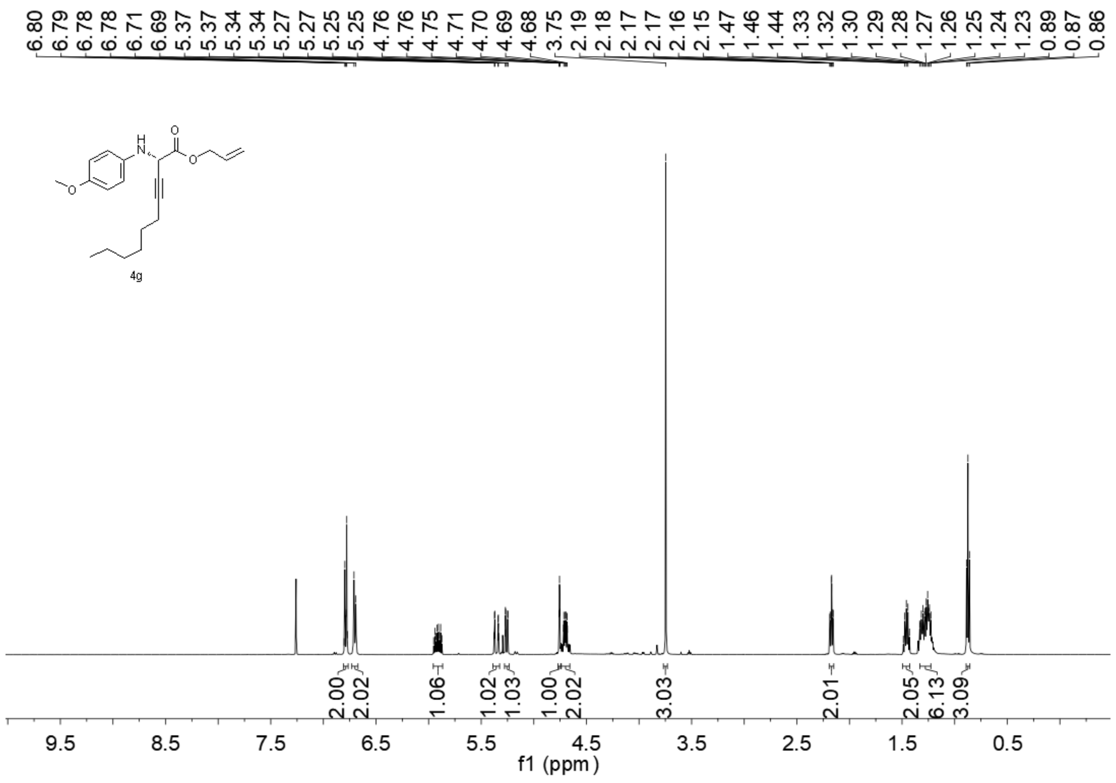
6.80
6.79
6.78
6.78
6.71
6.69
5.37
5.37
5.34
5.34
5.27
5.25
5.25
4.76
4.76
4.75
4.71
4.70
4.69
4.68
3.75
2.19
2.18
2.17
2.17
2.16
2.15
1.47
1.46
1.44
1.33
1.32
1.30
1.29
1.28
1.27
1.26
1.25
1.24
1.23
0.89
0.87
0.86
4g
2.00
2.02
1.06
1.02
1.03
1.00
2.02
3.03
2.01
2.05
6.13
3.09
9.5
8.5
7.5
6.5
5.5
4.5
3.5
2.5
1.5
0.5
f1 (ppm)

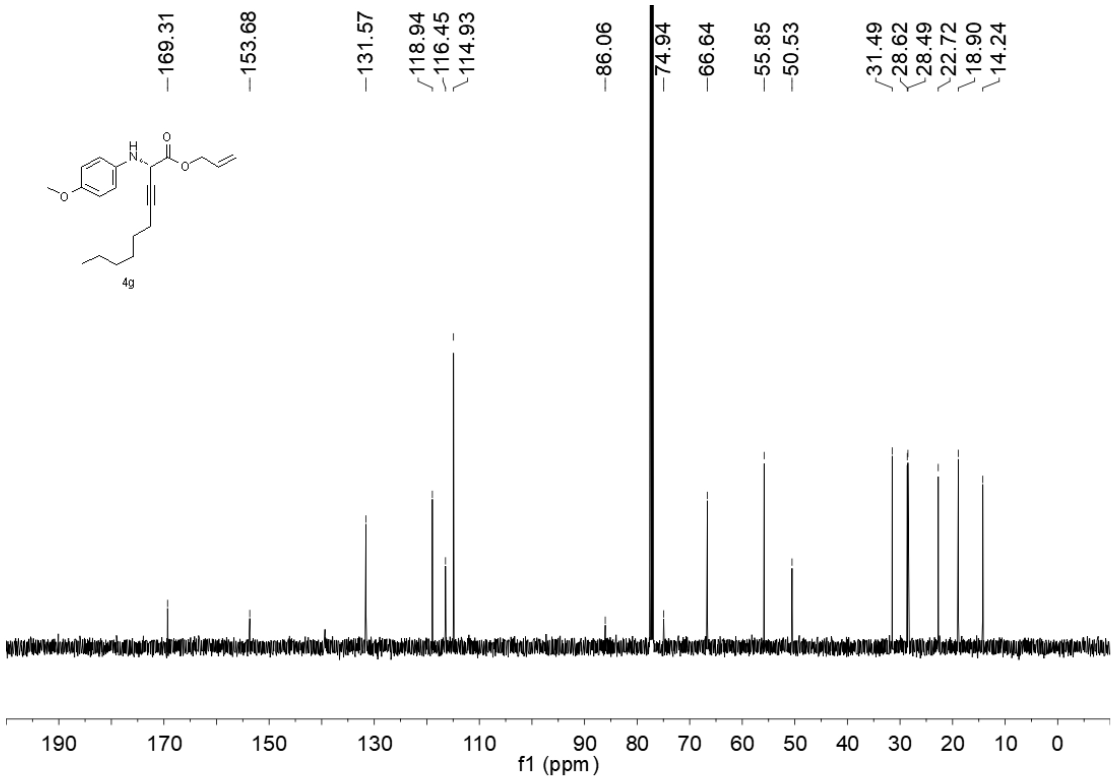
169.31
153.68
131.57
118.94
116.45
114.93
86.06
74.94
66.64
55.85
50.53
31.49
28.62
28.49
22.72
18.90
14.24
4g
190
170
150
130
110
90
80
70
60
50
40
30
20
10
0
f1 (ppm)

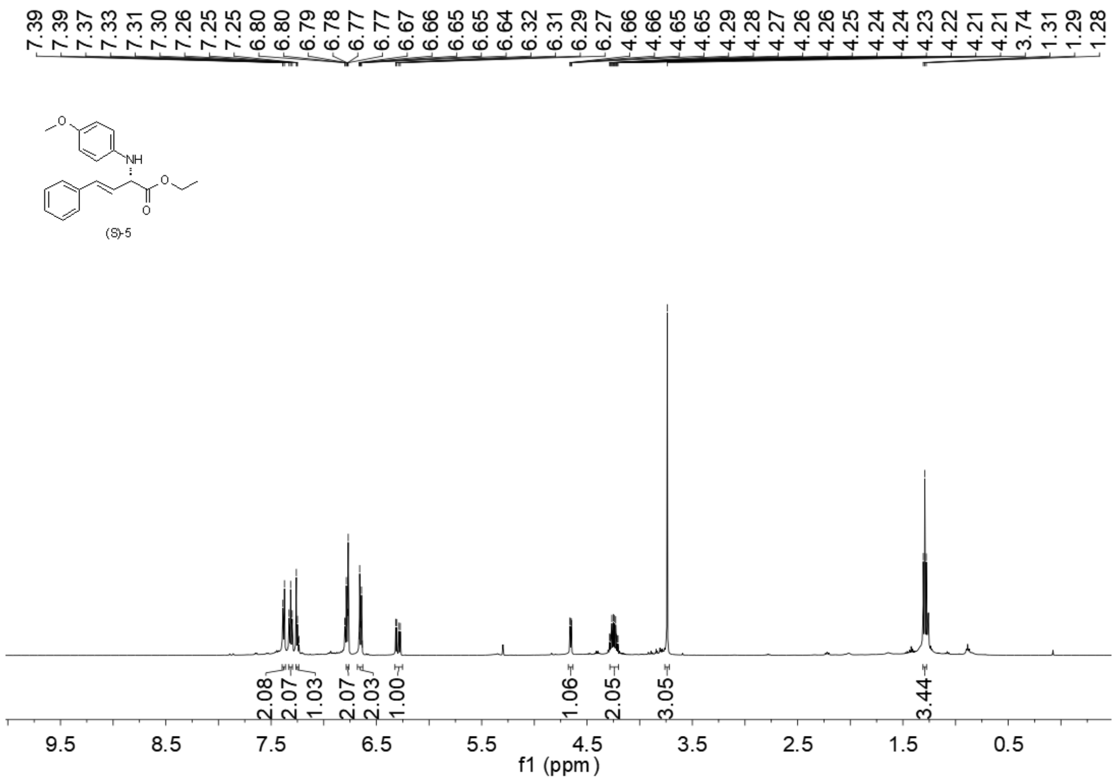
7.39 7.39 7.37 7.33 7.31 7.30 7.26 7.25 7.25 6.80 6.80 6.79 6.78 6.77 6.77 6.67 6.66 6.65 6.65 6.64 6.32 6.31 6.29 6.27 4.66 4.66 4.65 4.65 4.29 4.28 4.27 4.26 4.26 4.25 4.24 4.24 4.23 4.22 4.21 4.21 3.74 1.31 1.29 1.28
NH
O
(S)-5
2.08
2.07
1.03
2.07
2.03
1.00
1.06
2.05
3.05
3.44
9.5 8.5 7.5 6.5 5.5 4.5 3.5 2.5 1.5 0.5
f1 (ppm)

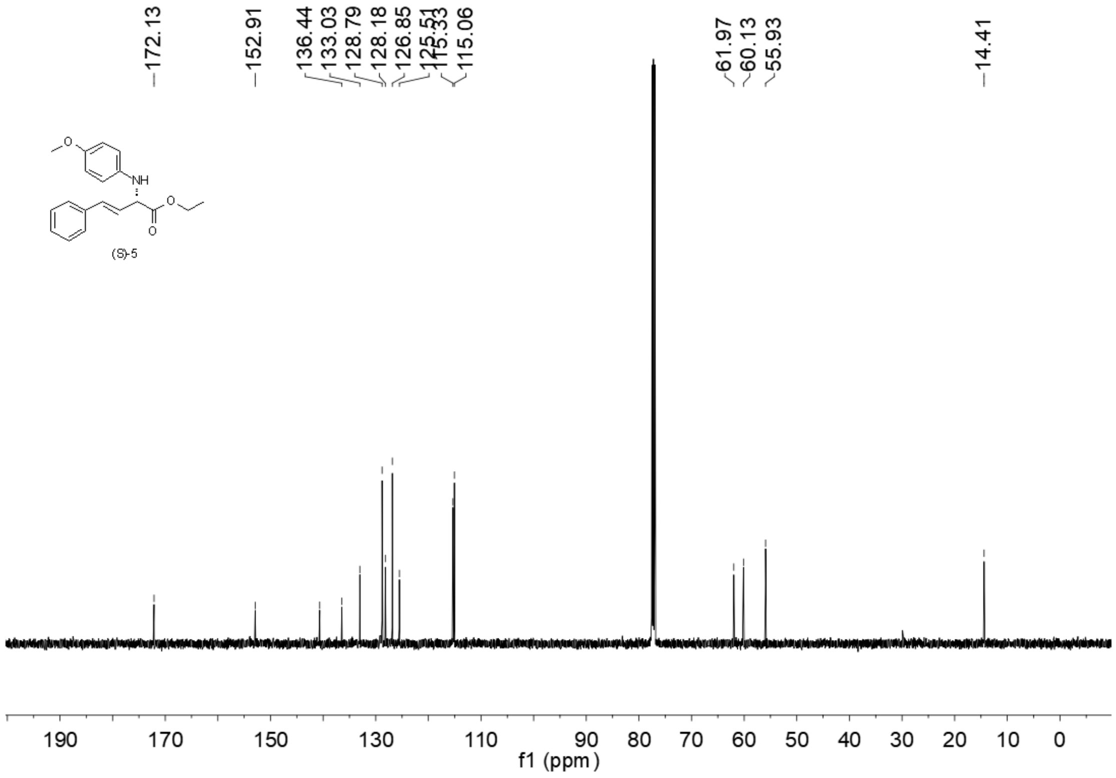
172.13
152.91
136.44 133.03 128.79 128.18 126.85 125.51 115.33 115.06
61.97 60.13 55.93
14.41
NH
O
(S)-5
190 170 150 130 110 90 80 70 60 50 40 30 20 10 0
f1 (ppm)

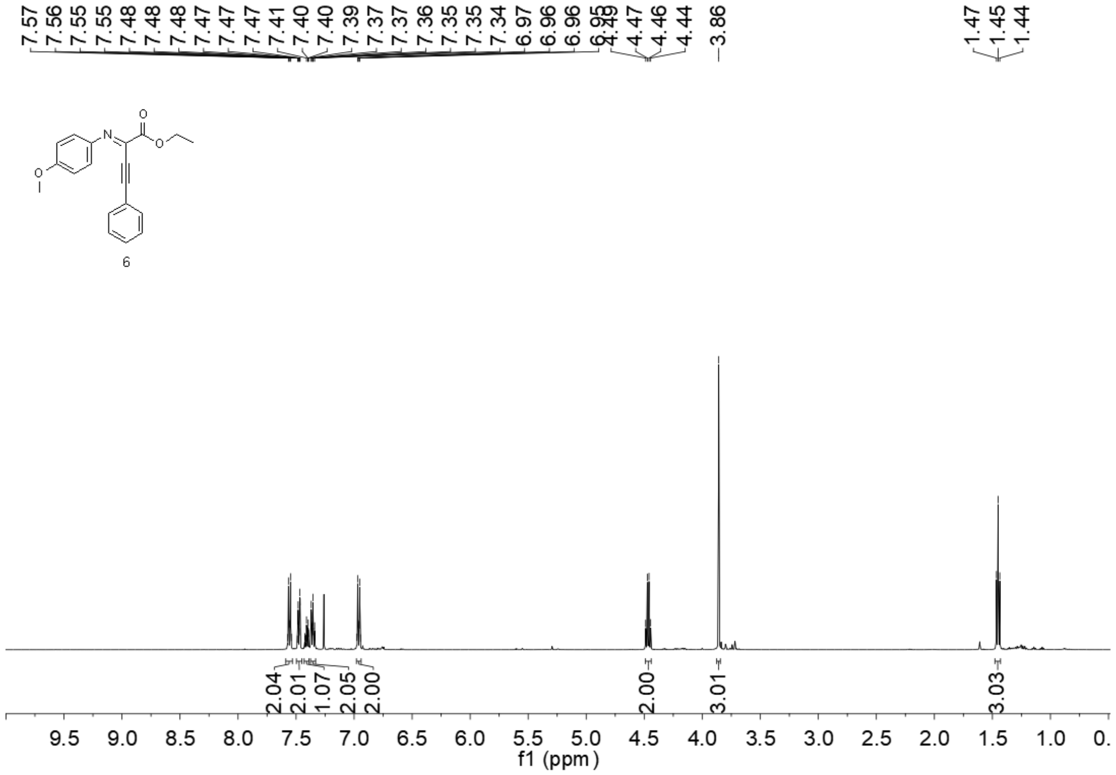
7.57
7.56
7.55
7.55
7.48
7.48
7.48
7.47
7.47
7.47
7.41
7.40
7.40
7.39
7.37
7.37
7.36
7.35
7.35
7.34
6.97
6.96
6.96
6.95
4.49
4.47
4.46
4.44
3.86
1.47
1.45
1.44
6
2.04
2.01
1.07
2.05
2.00
2.00
3.01
3.03
9.5 9.0 8.5 8.0 7.5 7.0 6.5 6.0 5.5 5.0 4.5 4.0 3.5 3.0 2.5 2.0 1.5 1.0 0.
f1 (ppm)

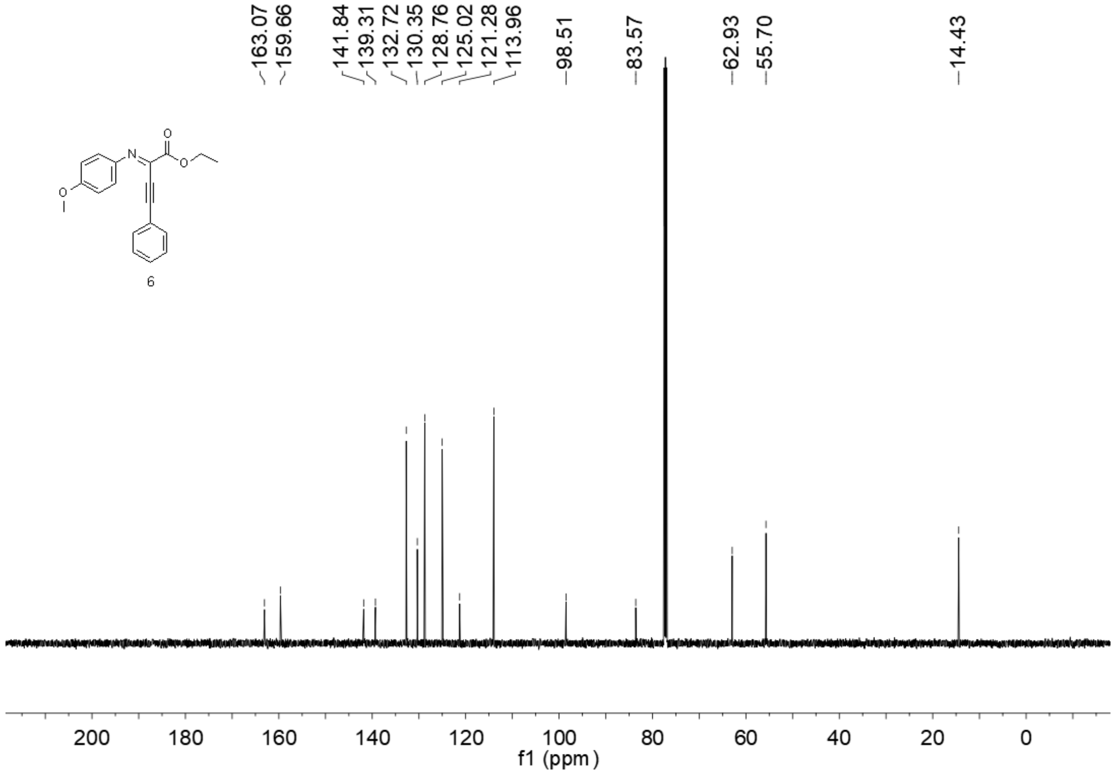
163.07
159.66
141.84
139.31
132.72
130.35
128.76
125.02
121.28
113.96
98.51
83.57
62.93
55.70
14.43
6
200 180 160 140 120 100 80 60 40 20 0
f1 (ppm)

HPLC spectra for ee determination

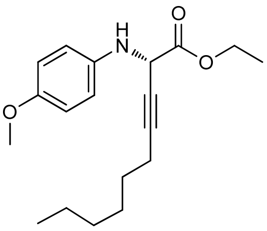

(1a)

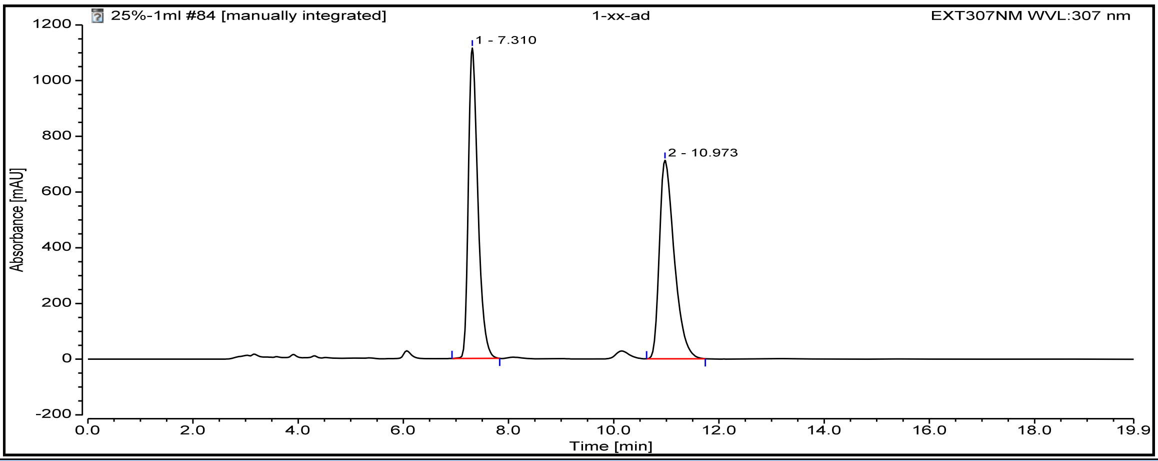

Integration Results

No.	Peak Name	Retention Time<br>min	Area<br>mAU*min	Relative Area<br>%	Amount<br>n.a.
1		7.310	233.965	50.00	n.a.
2		10.973	233.934	50.00	n.a.
**Total:**			**467.899**	**100.00**	

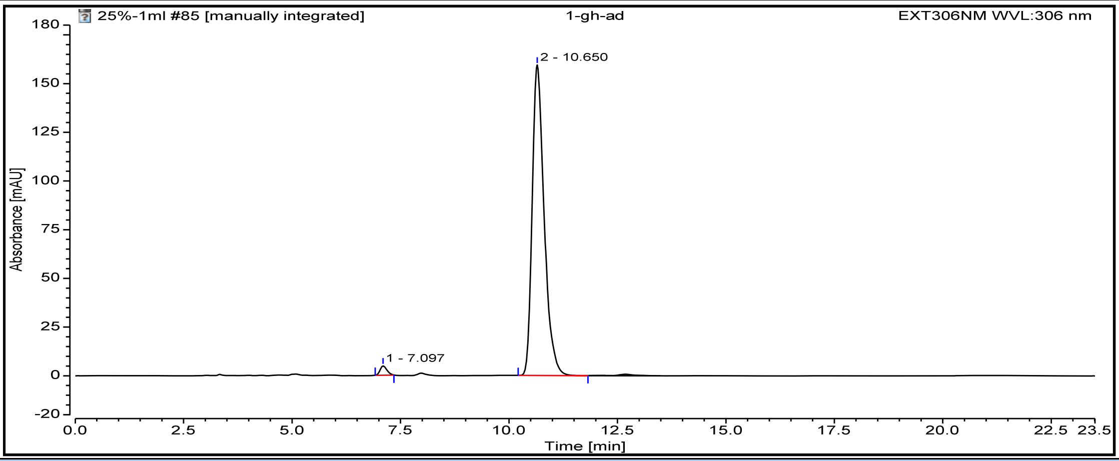

Integration Results

No.	Peak Name	Retention Time<br>min	Area<br>mAU*min	Relative Area<br>%	Amount<br>n.a.
1		7.097	0.881	1.69	n.a.
2		10.650	51.123	98.31	n.a.
**Total:**			**52.004**	**100.00**	

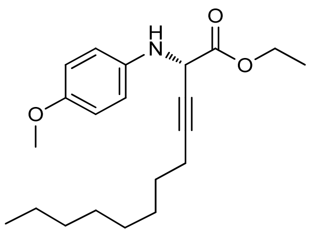

(1b)

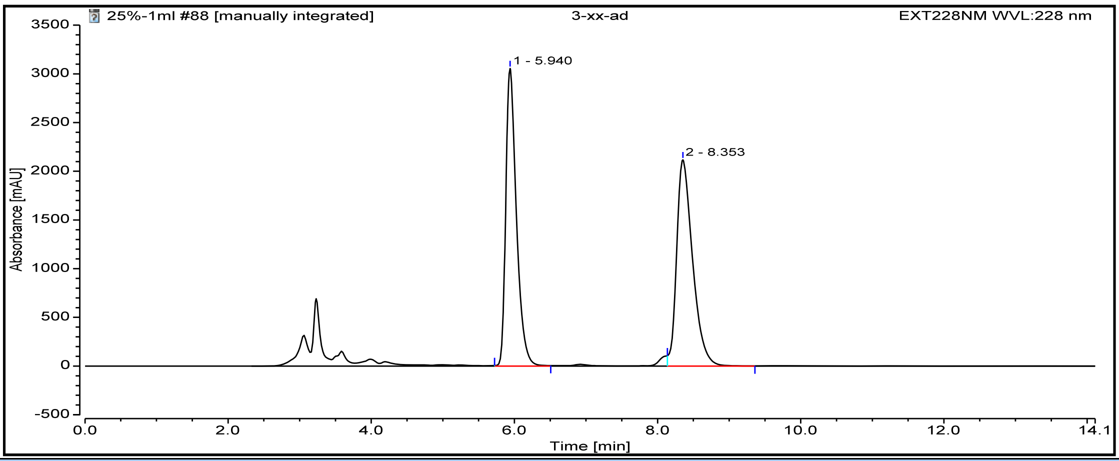

Integration Results					
No.	Peak Name	Retention Time<br>min	Area<br>mAU*min	Relative Area<br>%	Amount<br>n.a.
1		5.940	527.576	49.17	n.a.
2		8.353	545.452	50.83	n.a.
**Total:**			**1073.029**	**100.00**	

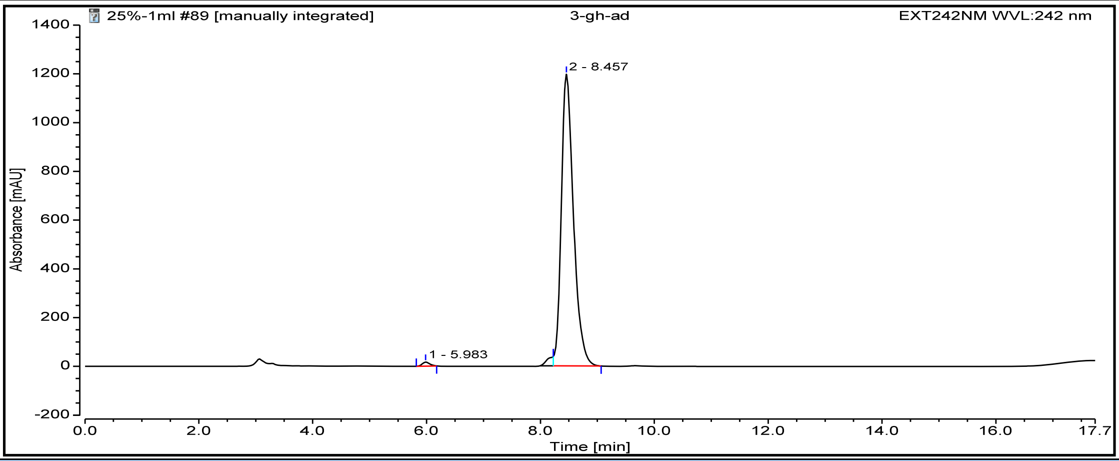

Integration Results					
No.	Peak Name	Retention Time<br>min	Area<br>mAU*min	Relative Area<br>%	Amount<br>n.a.
1		5.983	2.423	0.81	n.a.
2		8.457	296.469	99.19	n.a.
**Total:**			**298.893**	**100.00**	

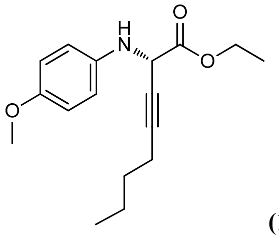

(1c)

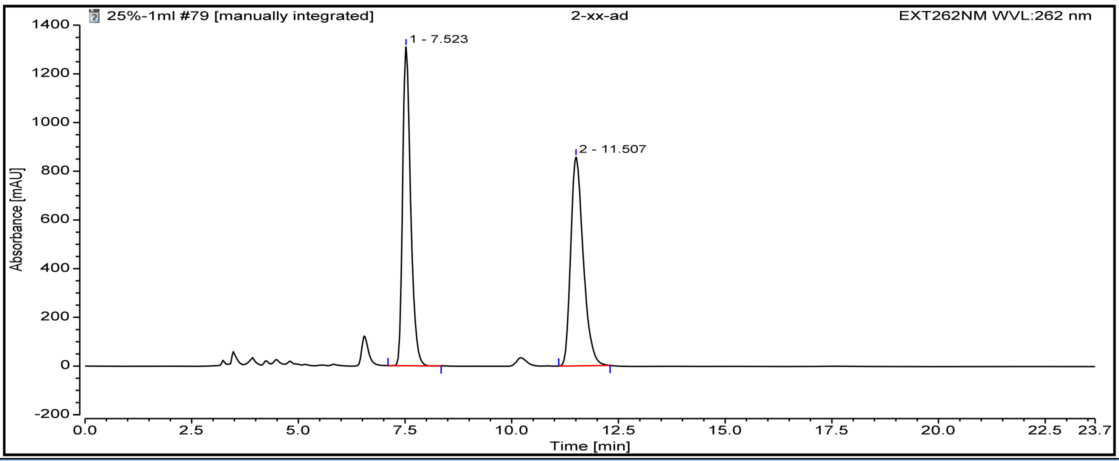

Integration Results					
No.	Peak Name	Retention Time min	Area mAU*min	Relative Area %	Amount n.a.
1		7.523	285.104	49.31	n.a.
2		11.507	293.084	50.69	n.a.
**Total:**			**578.187**	**100.00**	

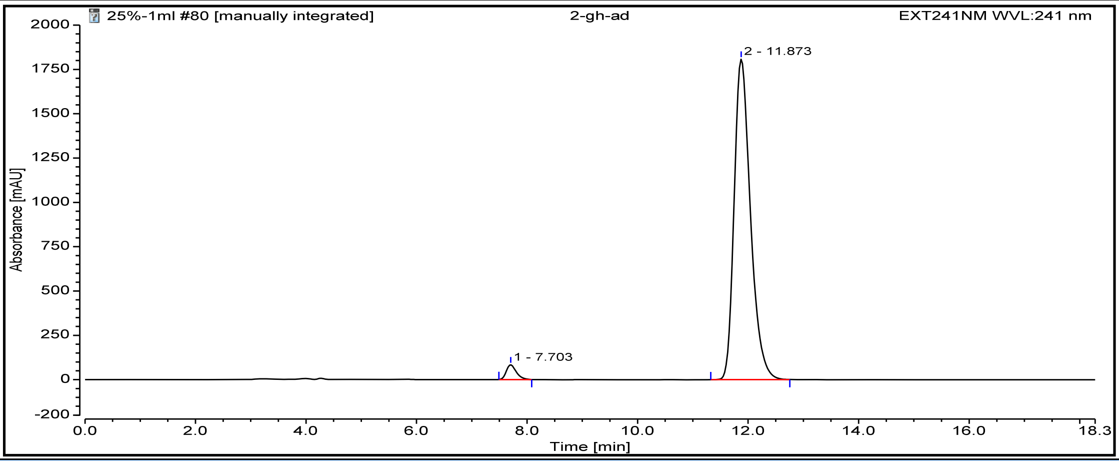

Integration Results					
No.	Peak Name	Retention Time min	Area mAU*min	Relative Area %	Amount n.a.
1		7.703	17.428	2.74	n.a.
2		11.873	618.833	97.26	n.a.
**Total:**			**636.261**	**100.00**	

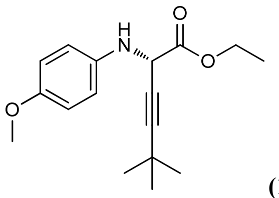

(1d)

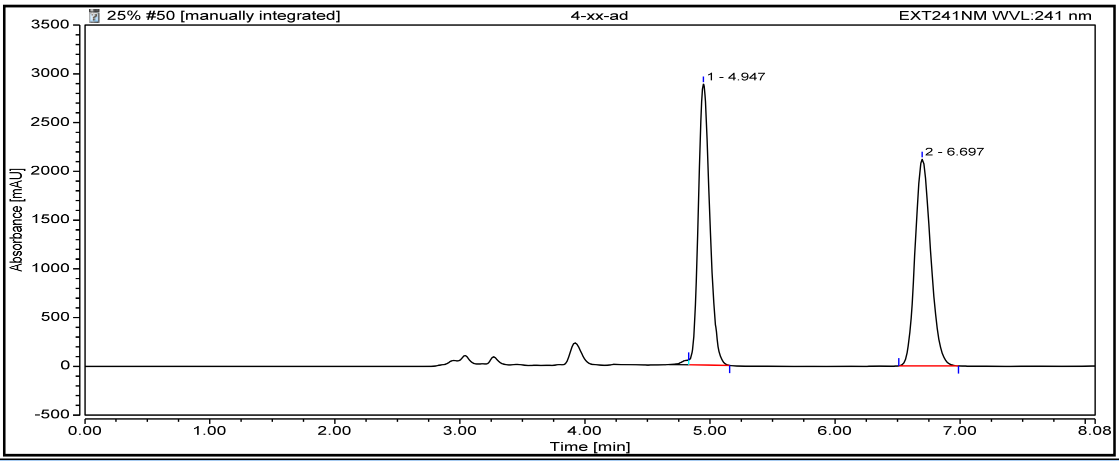

**Integration Results**					
No.	Peak Name	Retention Time<br>min	Area<br>mAU*min	Relative Area<br>%	Amount<br>n.a.
1		4.947	304.953	49.67	n.a.
2		6.697	308.990	50.33	n.a.
**Total:**			**613.943**	**100.00**	

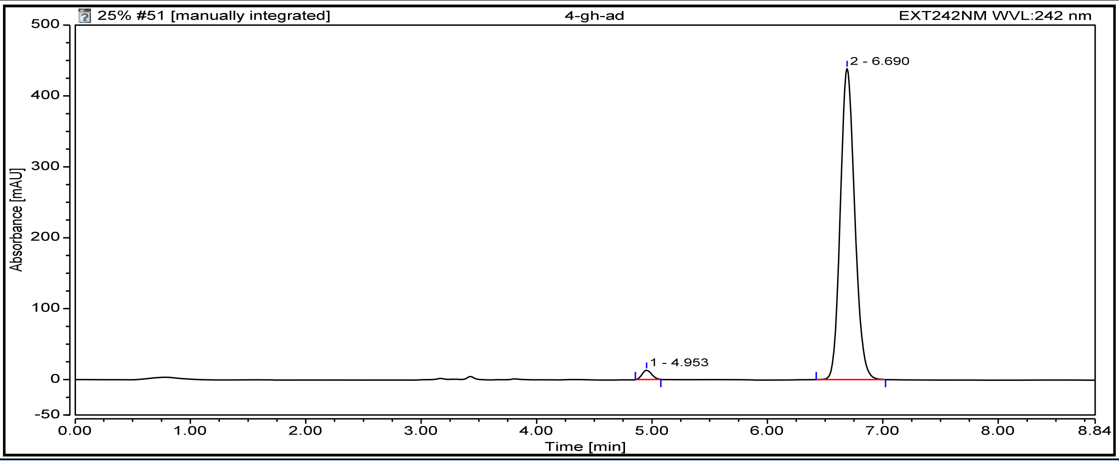

**Integration Results**					
No.	Peak Name	Retention Time<br>min	Area<br>mAU*min	Relative Area<br>%	Amount<br>n.a.
1		4.953	1.273	1.97	n.a.
2		6.690	63.380	98.03	n.a.
**Total:**			**64.652**	**100.00**	

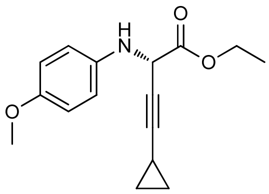

(1e)

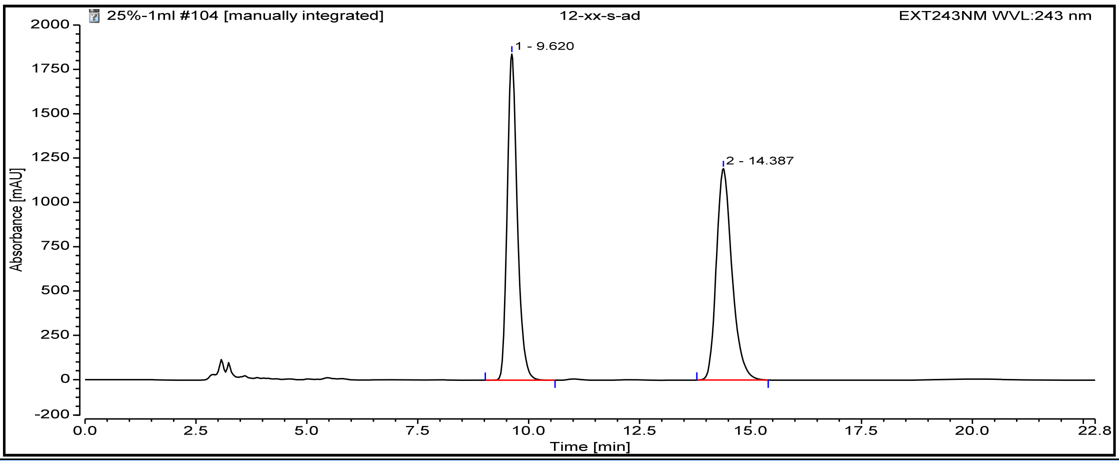

Integration Results					
No.	Peak Name	Retention Time<br>min	Area<br>mAU*min	Relative Area<br>%	Amount<br>n.a.
1		9.620	487.754	49.91	n.a.
2		14.387	489.602	50.09	n.a.
**Total:**			**977.356**	**100.00**	

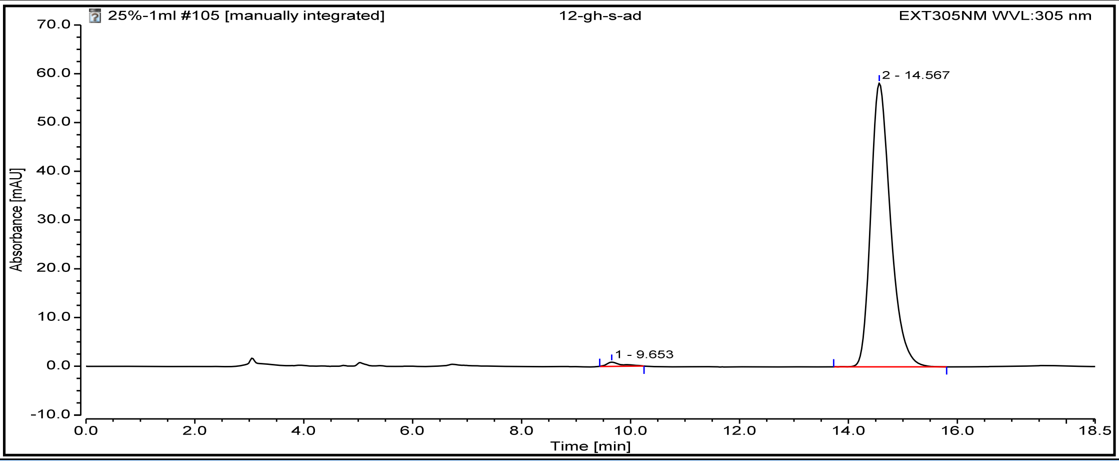

Integration Results					
No.	Peak Name	Retention Time<br>min	Area<br>mAU*min	Relative Area<br>%	Amount<br>n.a.
1		9.653	0.278	1.12	n.a.
2		14.567	24.562	98.88	n.a.
**Total:**			**24.840**	**100.00**	

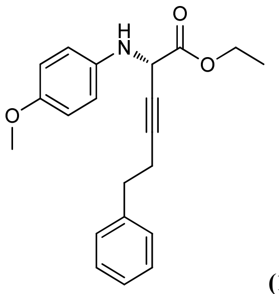

(1f)

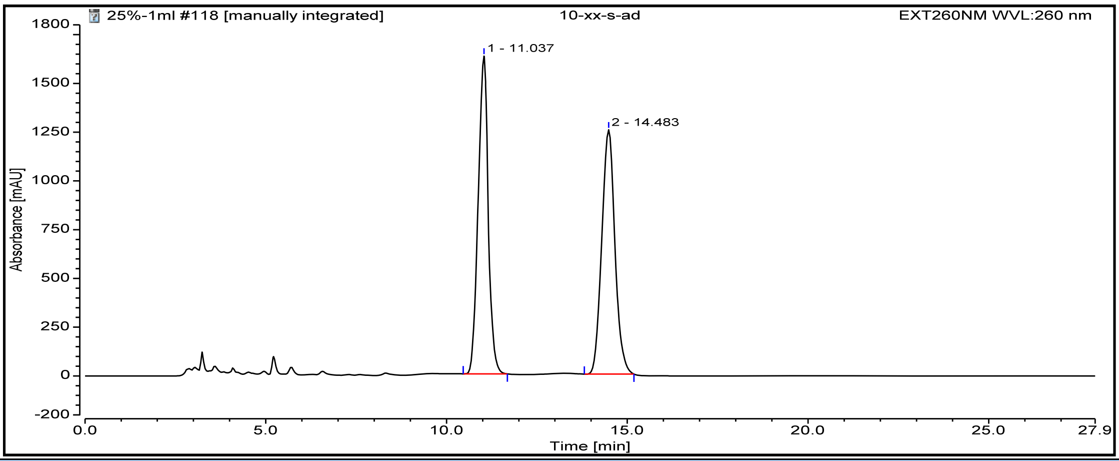

Integration Results					
No.	Peak Name	Retention Time min	Area mAU*min	Relative Area %	Amount n.a.
1		11.037	522.123	49.82	n.a.
2		14.483	525.979	50.18	n.a.
**Total:**			**1048.102**	**100.00**	

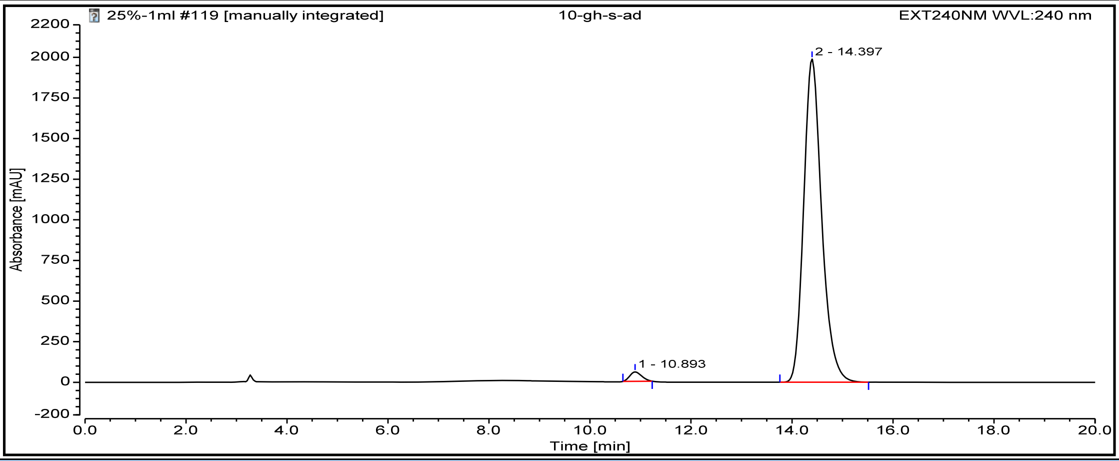

Integration Results					
No.	Peak Name	Retention Time min	Area mAU*min	Relative Area %	Amount n.a.
1		10.893	15.700	1.87	n.a.
2		14.397	824.985	98.13	n.a.
**Total:**			**840.685**	**100.00**	

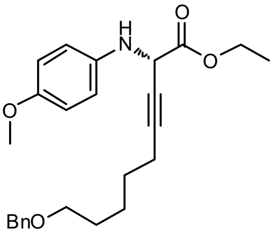

(1g)

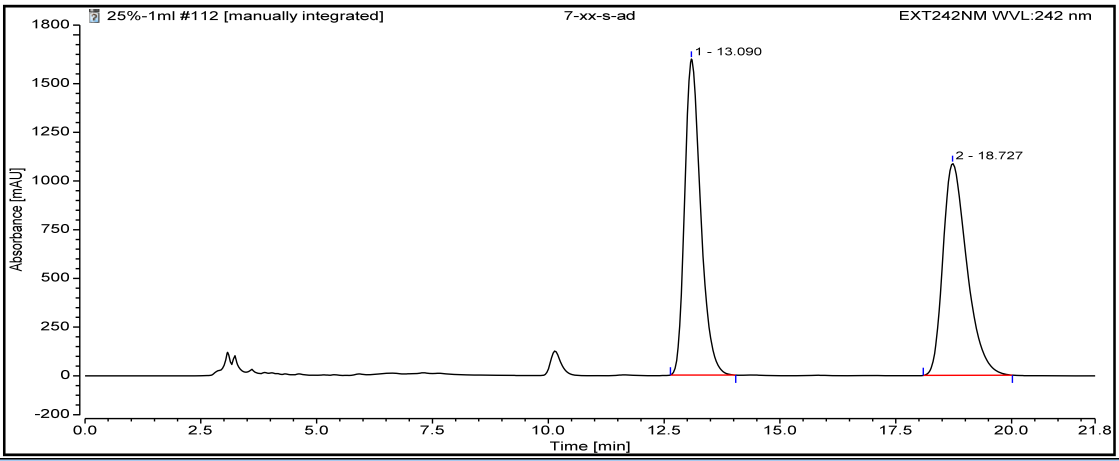

Integration Results					
No.	Peak Name	Retention Time<br>min	Area<br>mAU*min	Relative Area<br>%	Amount<br>n.a.
1		13.090	650.662	49.93	n.a.
2		18.727	652.501	50.07	n.a.
**Total:**			**1303.163**	**100.00**	

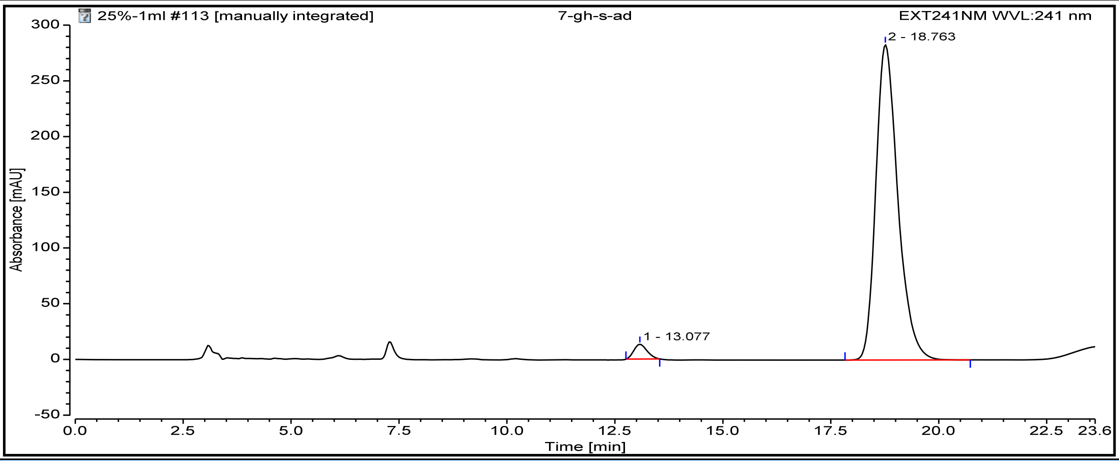

Integration Results					
No.	Peak Name	Retention Time<br>min	Area<br>mAU*min	Relative Area<br>%	Amount<br>n.a.
1		13.077	4.694	2.73	n.a.
2		18.763	167.356	97.27	n.a.
**Total:**			**172.050**	**100.00**	

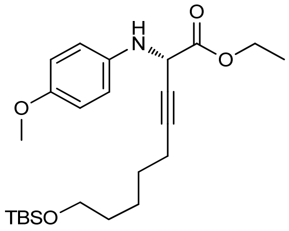

(1h)

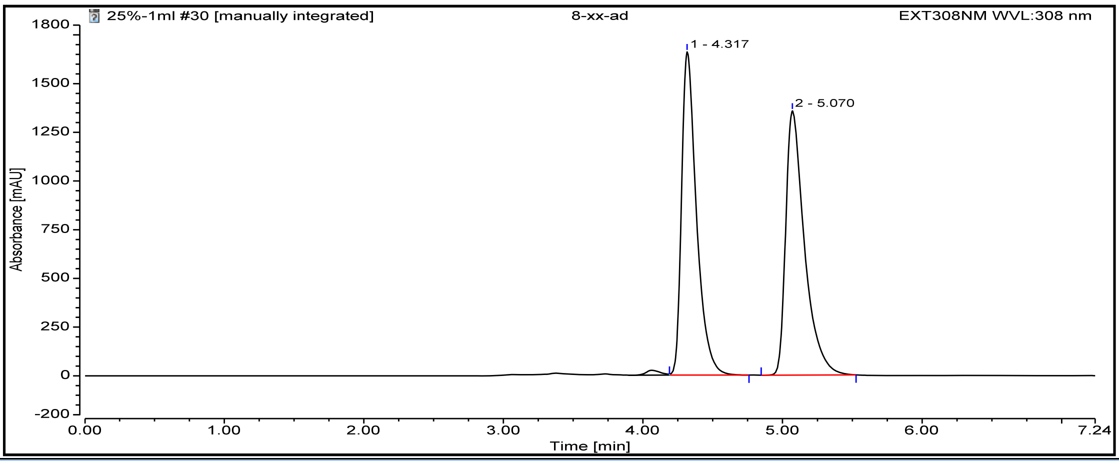

Integration Results					
No.	Peak Name	Retention Time<br>min	Area<br>mAU*min	Relative Area<br>%	Amount<br>n.a.
1		4.317	205.693	49.29	n.a.
2		5.070	211.581	50.71	n.a.
**Total:**			**417.274**	**100.00**	

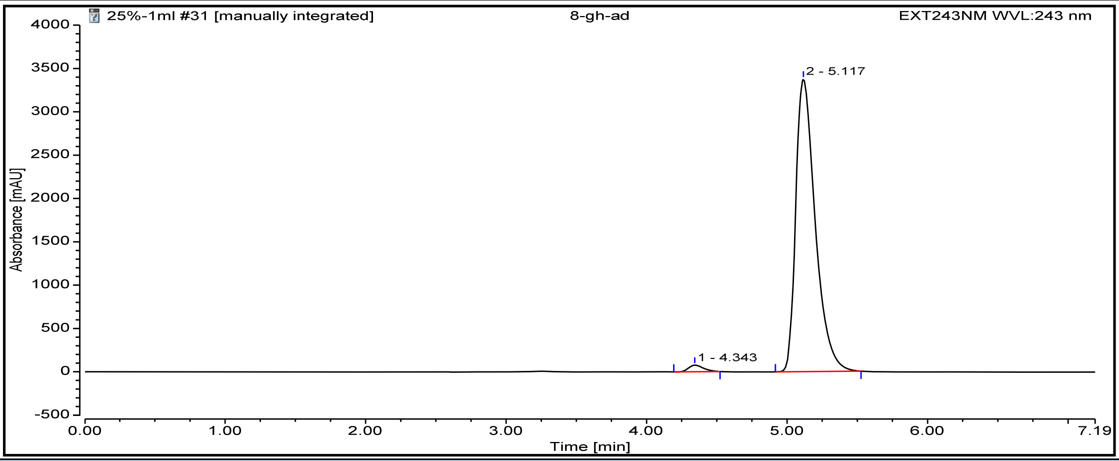

Integration Results					
No.	Peak Name	Retention Time<br>min	Area<br>mAU*min	Relative Area<br>%	Amount<br>n.a.
1		4.343	9.387	1.70	n.a.
2		5.117	542.404	98.30	n.a.
**Total:**			**551.791**	**100.00**	

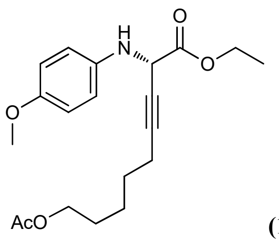

(1i)

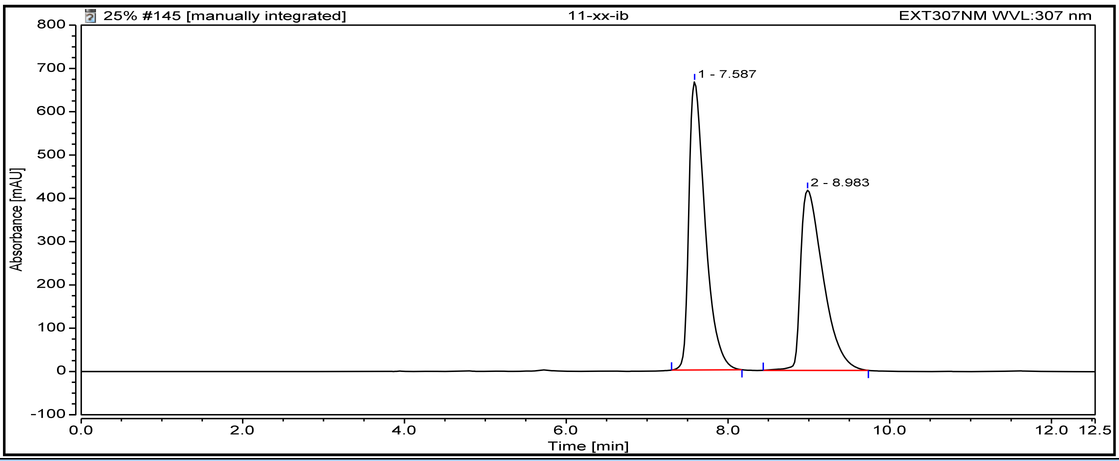

Integration Results					
No.	Peak Name	Retention Time min	Area mAU*min	Relative Area %	Amount n.a.
1		7.587	155.433	53.62	n.a.
2		8.983	134.441	46.38	n.a.
**Total:**			**289.874**	**100.00**	

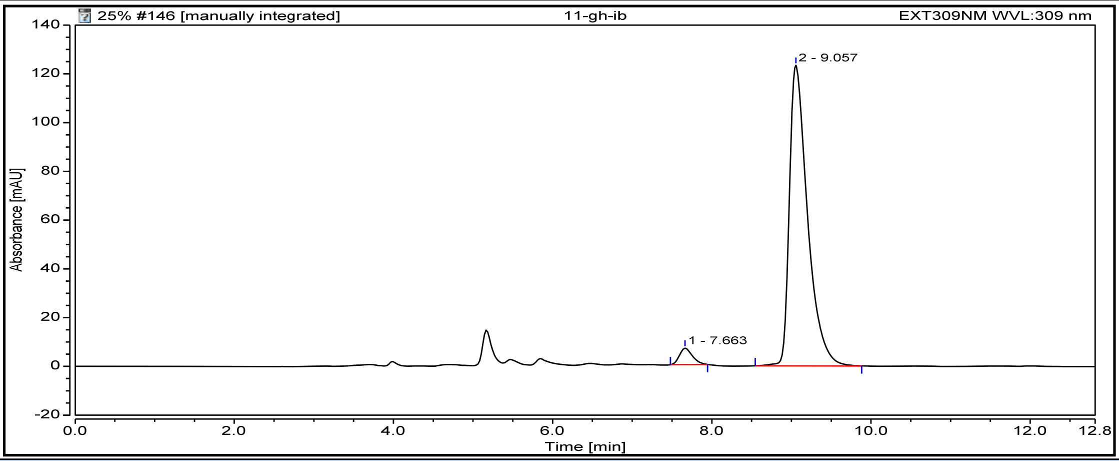

Integration Results					
No.	Peak Name	Retention Time min	Area mAU*min	Relative Area %	Amount n.a.
1		7.663	1.266	3.73	n.a.
2		9.057	32.649	96.27	n.a.
**Total:**			**33.915**	**100.00**	

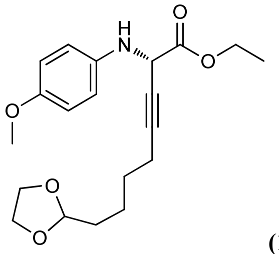

(1j)

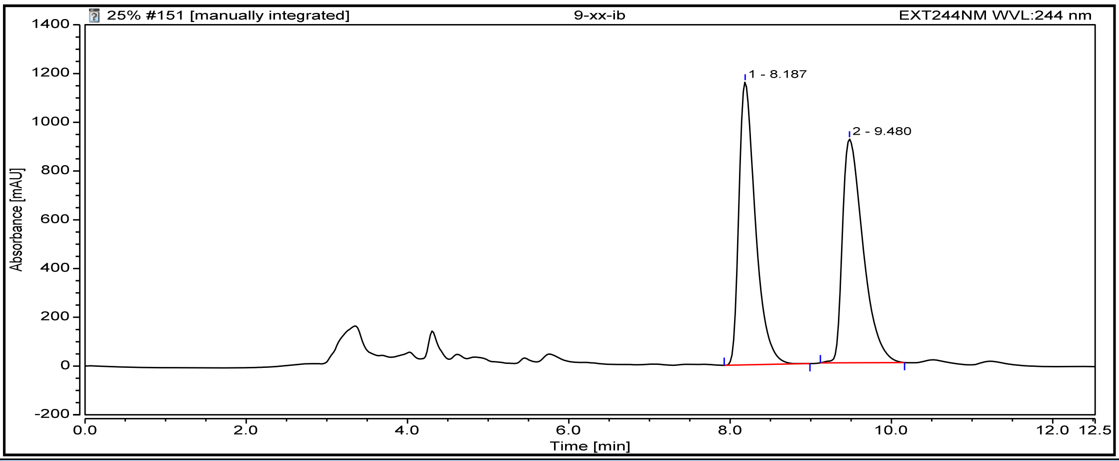

**Integration Results**					
No.	Peak Name	Retention Time<br>min	Area<br>mAU*min	Relative Area<br>%	Amount<br>n.a.
1		8.187	275.885	50.31	n.a.
2		9.480	272.469	49.69	n.a.
**Total:**			**548.354**	**100.00**	

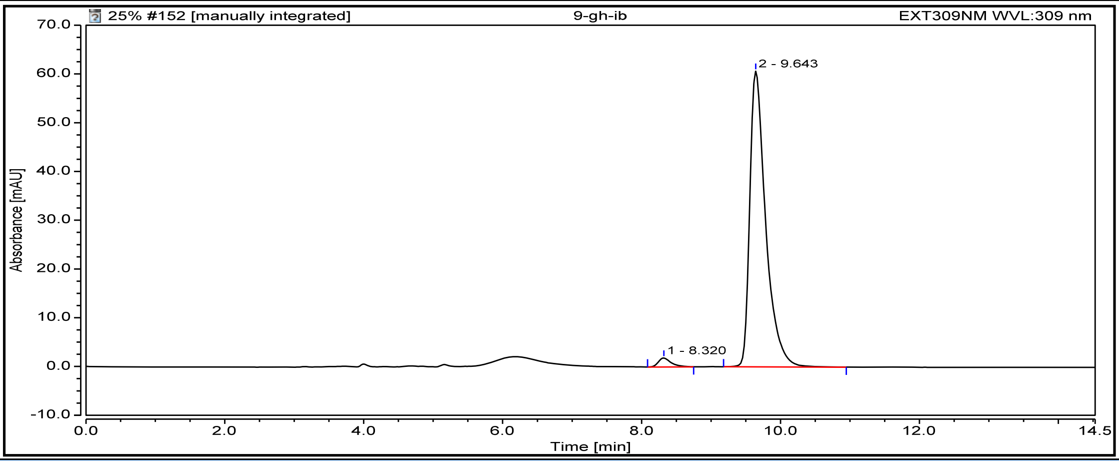

**Integration Results**					
No.	Peak Name	Retention Time<br>min	Area<br>mAU*min	Relative Area<br>%	Amount<br>n.a.
1		8.320	0.392	2.38	n.a.
2		9.643	16.125	97.62	n.a.
**Total:**			**16.517**	**100.00**	

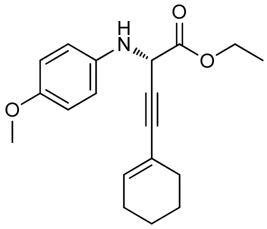

(1k)

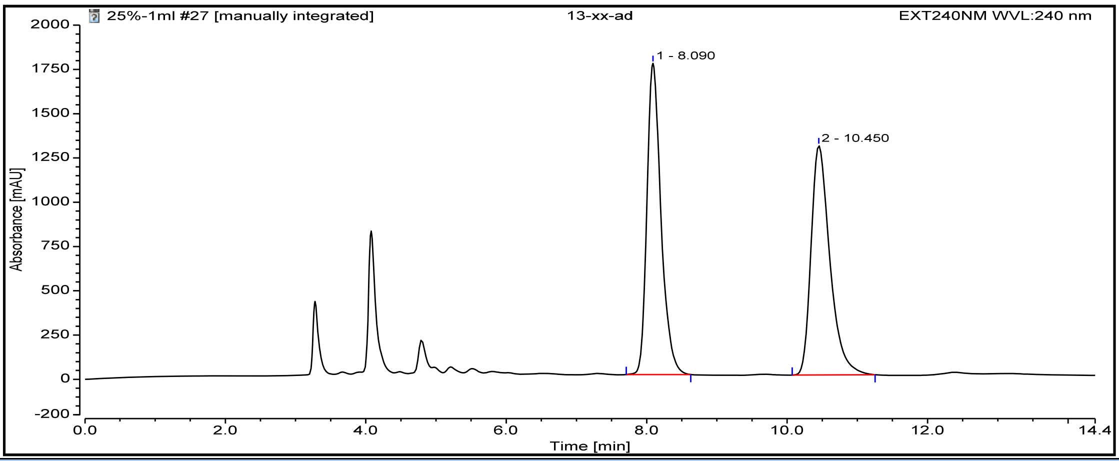

Integration Results					
No.	Peak Name	Retention Time<br>min	Area<br>mAU*min	Relative Area<br>%	Amount<br>n.a.
1		8.090	407.629	50.31	n.a.
2		10.450	402.658	49.69	n.a.
**Total:**			**810.286**	**100.00**	

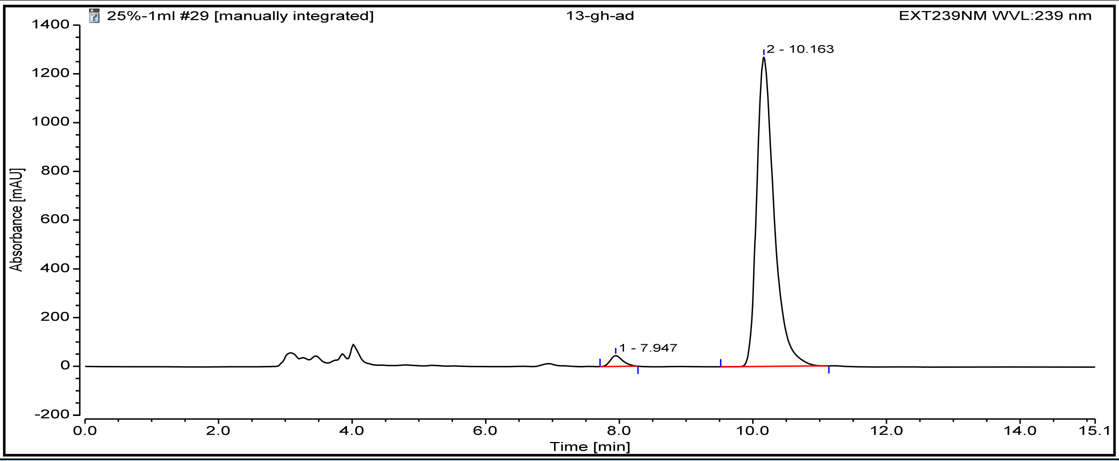

Integration Results					
No.	Peak Name	Retention Time<br>min	Area<br>mAU*min	Relative Area<br>%	Amount<br>n.a.
1		7.947	9.724	2.48	n.a.
2		10.163	382.610	97.52	n.a.
**Total:**			**392.334**	**100.00**	

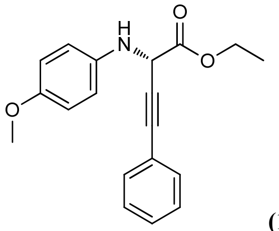

(1l)

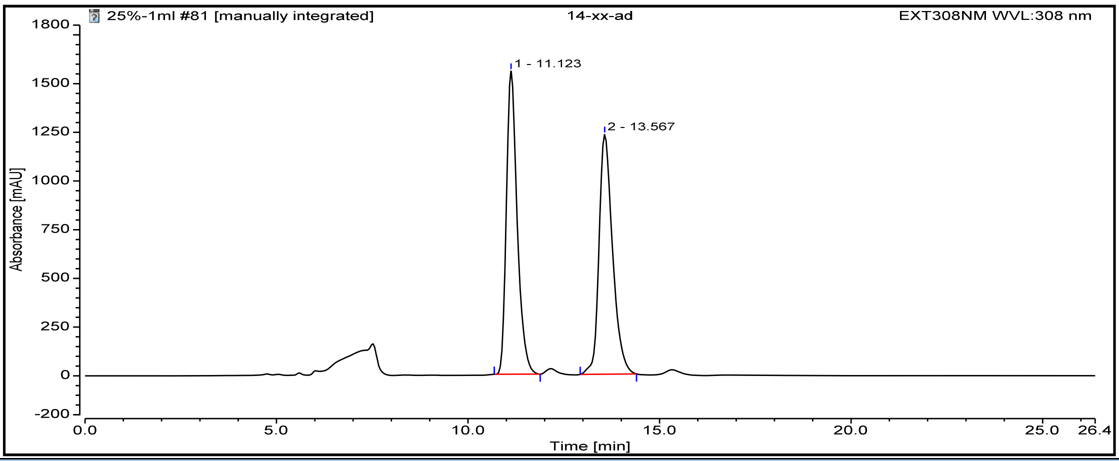

Integration Results					
No.	Peak Name	Retention Time<br>min	Area<br>mAU*min	Relative Area<br>%	Amount<br>n.a.
1		11.123	510.180	50.22	n.a.
2		13.567	505.762	49.78	n.a.
**Total:**			**1015.942**	**100.00**	

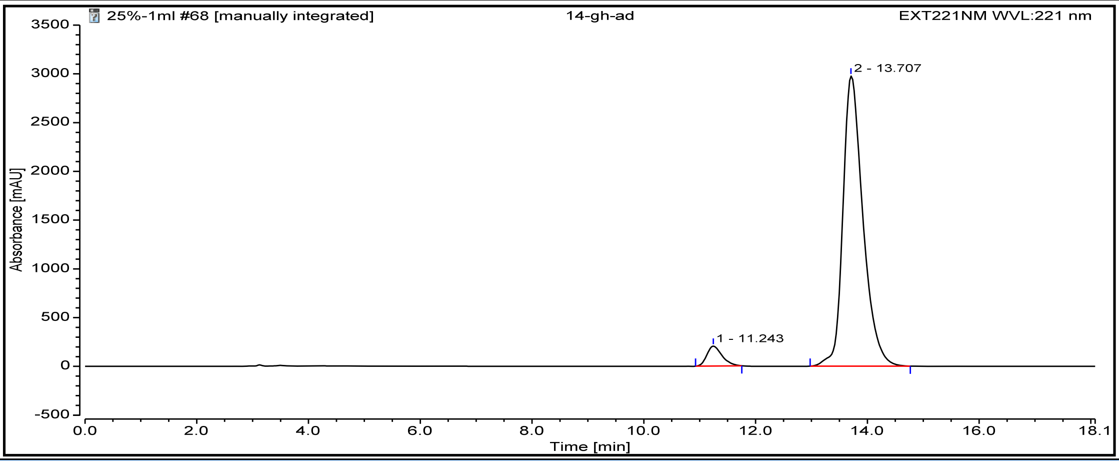

Integration Results					
No.	Peak Name	Retention Time<br>min	Area<br>mAU*min	Relative Area<br>%	Amount<br>n.a.
1		11.243	64.337	4.94	n.a.
2		13.707	1238.828	95.06	n.a.
**Total:**			**1303.166**	**100.00**	

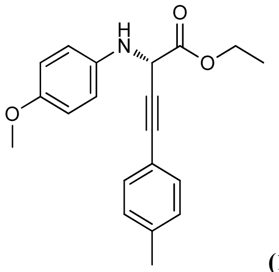

(1m)

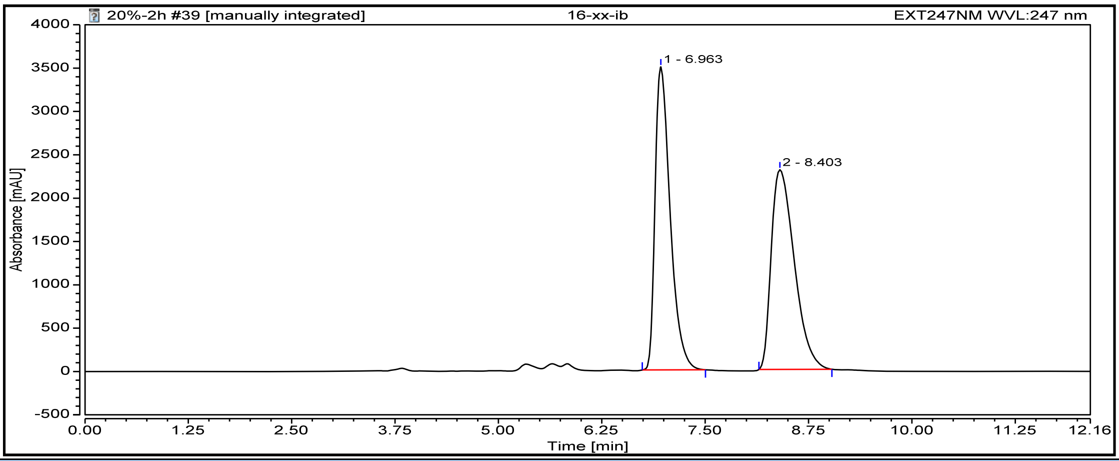

Integration Results					
No.	Peak Name	Retention Time<br>min	Area<br>mAU*min	Relative Area<br>%	Amount<br>n.a.
1		6.963	740.907	49.90	n.a.
2		8.403	743.936	50.10	n.a.
**Total:**			**1484.843**	**100.00**	

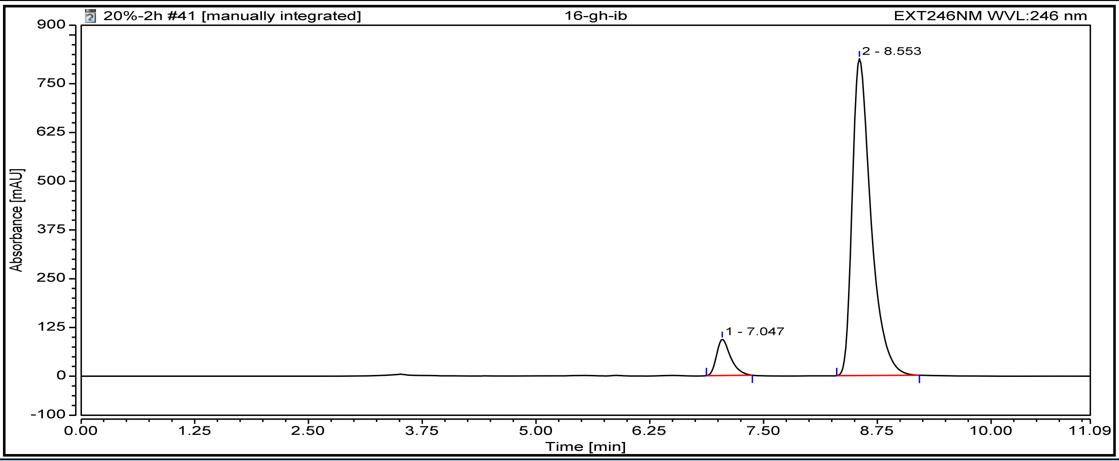

Integration Results					
No.	Peak Name	Retention Time<br>min	Area<br>mAU*min	Relative Area<br>%	Amount<br>n.a.
1		7.047	16.687	7.90	n.a.
2		8.553	194.609	92.10	n.a.
**Total:**			**211.295**	**100.00**	

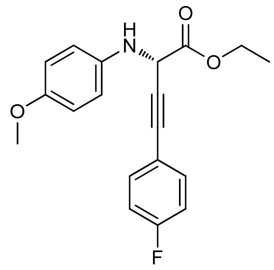

(1n)

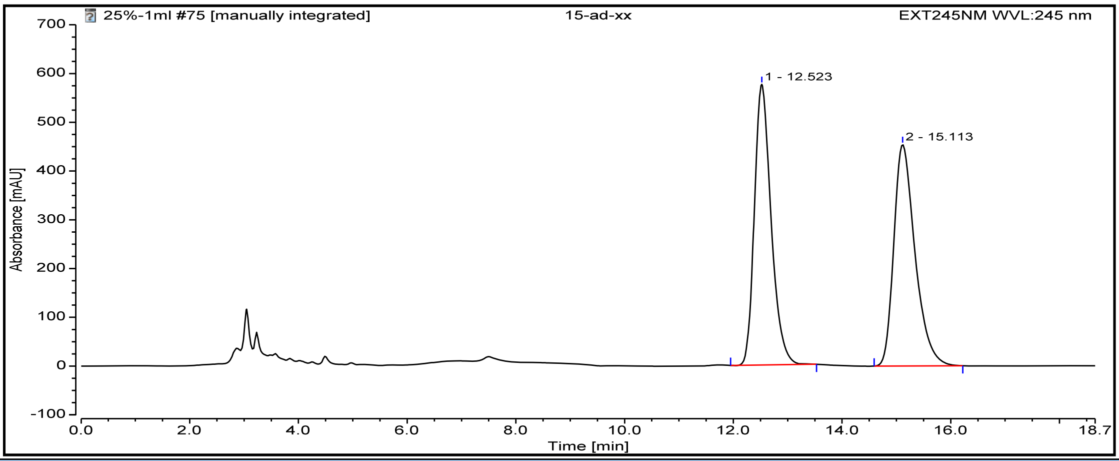

Integration Results					
No.	Peak Name	Retention Time min	Area mAU*min	Relative Area %	Amount n.a.
1		12.523	204.781	49.93	n.a.
2		15.113	205.395	50.07	n.a.
**Total:**			**410.175**	**100.00**	

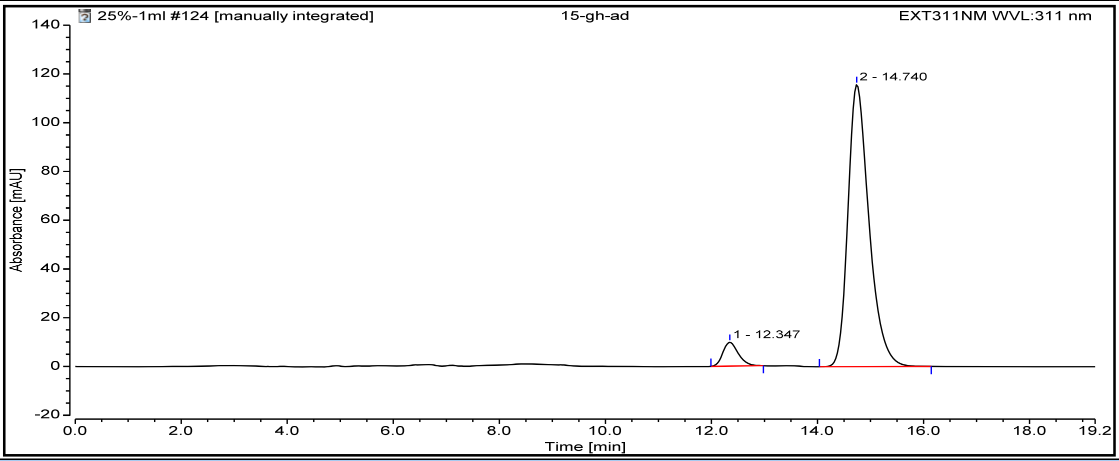

Integration Results					
No.	Peak Name	Retention Time min	Area mAU*min	Relative Area %	Amount n.a.
1		12.347	3.280	5.80	n.a.
2		14.740	53.294	94.20	n.a.
**Total:**			**56.574**	**100.00**	

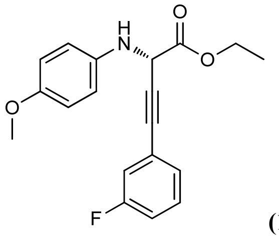

(1o)

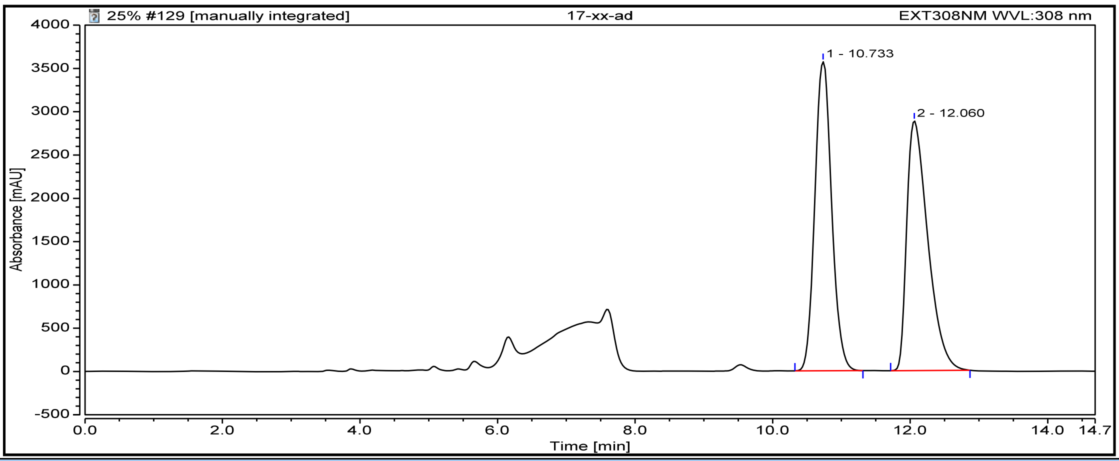

**Integration Results**					
No.	Peak Name	Retention Time<br>min	Area<br>mAU*min	Relative Area<br>%	Amount<br>n.a.
1		10.733	1013.598	49.68	n.a.
2		12.060	1026.619	50.32	n.a.
**Total:**			**2040.217**	**100.00**	

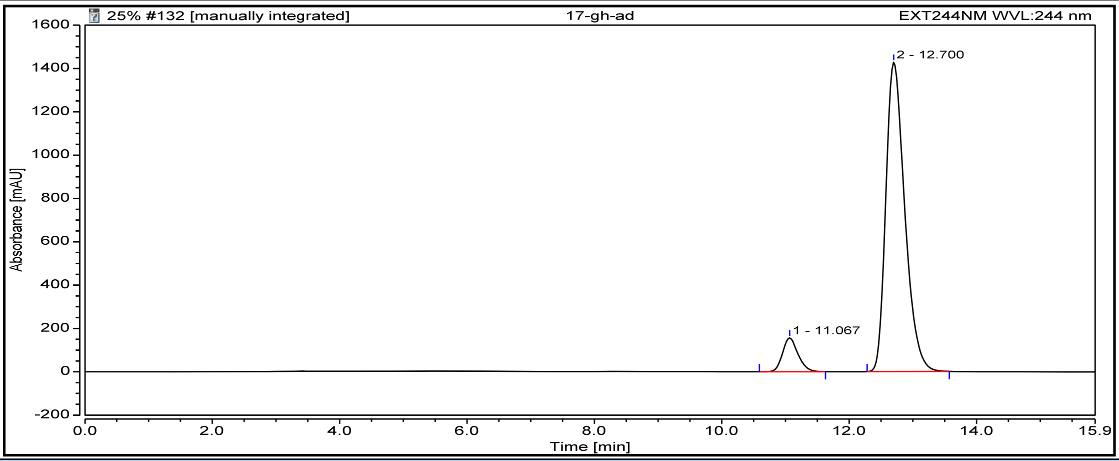

**Integration Results**					
No.	Peak Name	Retention Time<br>min	Area<br>mAU*min	Relative Area<br>%	Amount<br>n.a.
1		11.067	43.841	8.18	n.a.
2		12.700	492.285	91.82	n.a.
**Total:**			**536.127**	**100.00**	

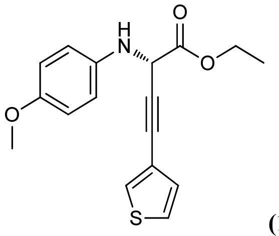

(1p)

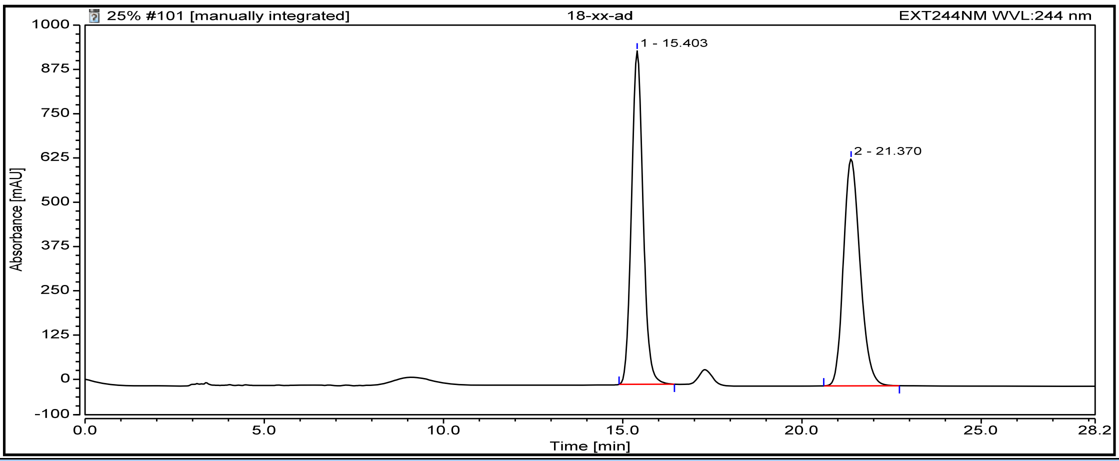

**Integration Results**					
No.	Peak Name	Retention Time<br>min	Area<br>mAU*min	Relative Area<br>%	Amount<br>n.a.
1		15.403	351.094	50.44	n.a.
2		21.370	344.982	49.56	n.a.
**Total:**			**696.076**	**100.00**	

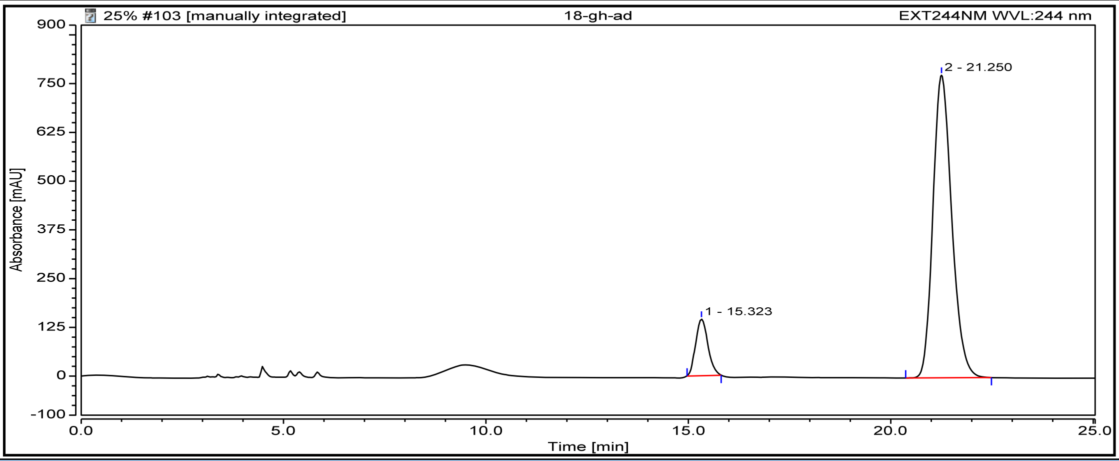

**Integration Results**					
No.	Peak Name	Retention Time<br>min	Area<br>mAU*min	Relative Area<br>%	Amount<br>n.a.
1		15.323	51.394	10.96	n.a.
2		21.250	417.512	89.04	n.a.
**Total:**			**468.906**	**100.00**	

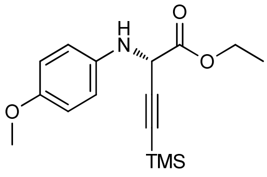

(1q)

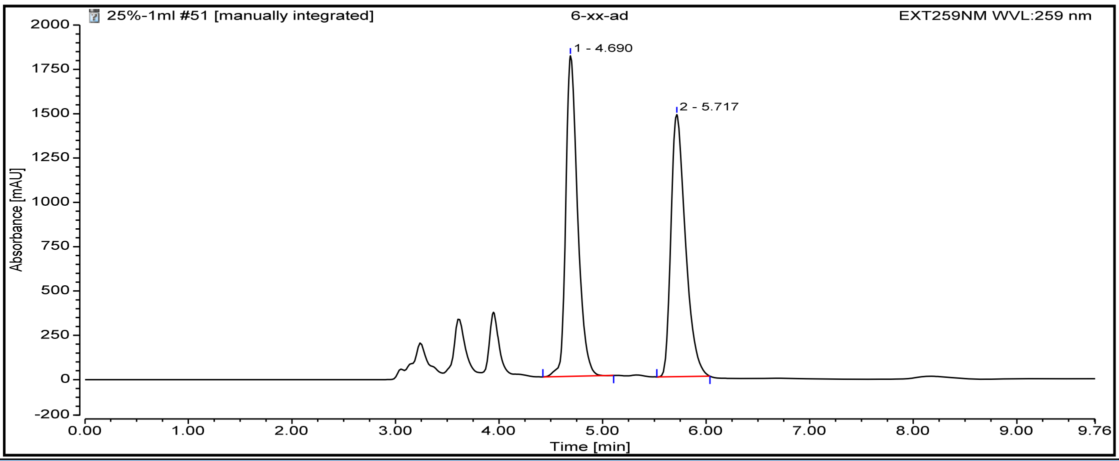

Integration Results					
No.	Peak Name	Retention Time<br>min	Area<br>mAU*min	Relative Area<br>%	Amount<br>n.a.
1		4.690	239.773	49.94	n.a.
2		5.717	240.330	50.06	n.a.
**Total:**			**480.103**	**100.00**	

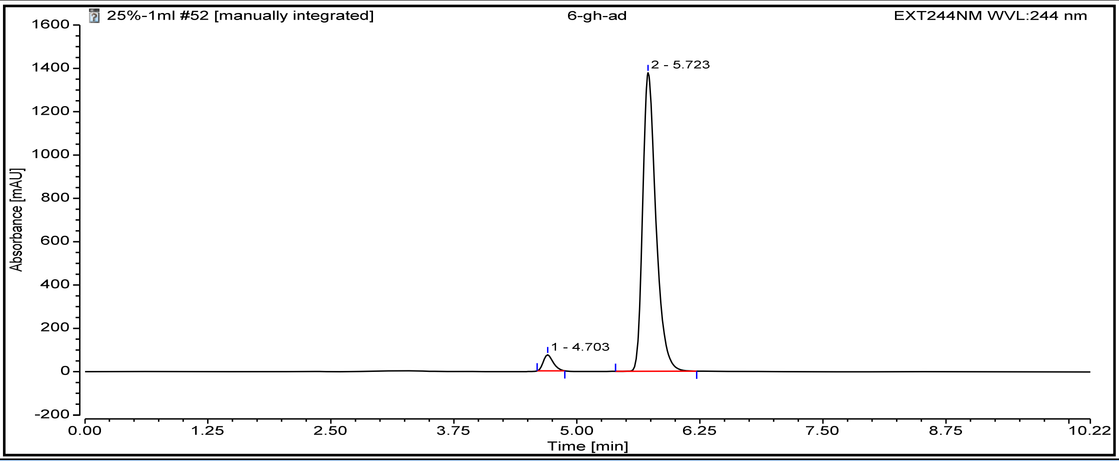

Integration Results					
No.	Peak Name	Retention Time<br>min	Area<br>mAU*min	Relative Area<br>%	Amount<br>n.a.
1		4.703	8.629	3.86	n.a.
2		5.723	214.915	96.14	n.a.
**Total:**			**223.544**	**100.00**	

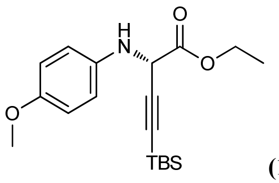

(1r)

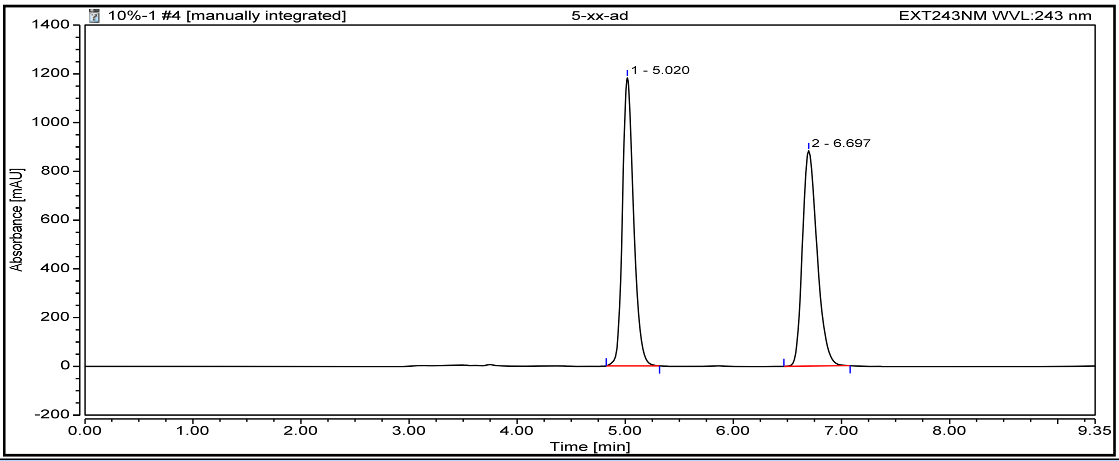

Integration Results					
No.	Peak Name	Retention Time<br>min	Area<br>mAU*min	Relative Area<br>%	Amount<br>n.a.
1		5.020	141.666	50.32	n.a.
2		6.697	139.851	49.68	n.a.
**Total:**			**281.516**	**100.00**	

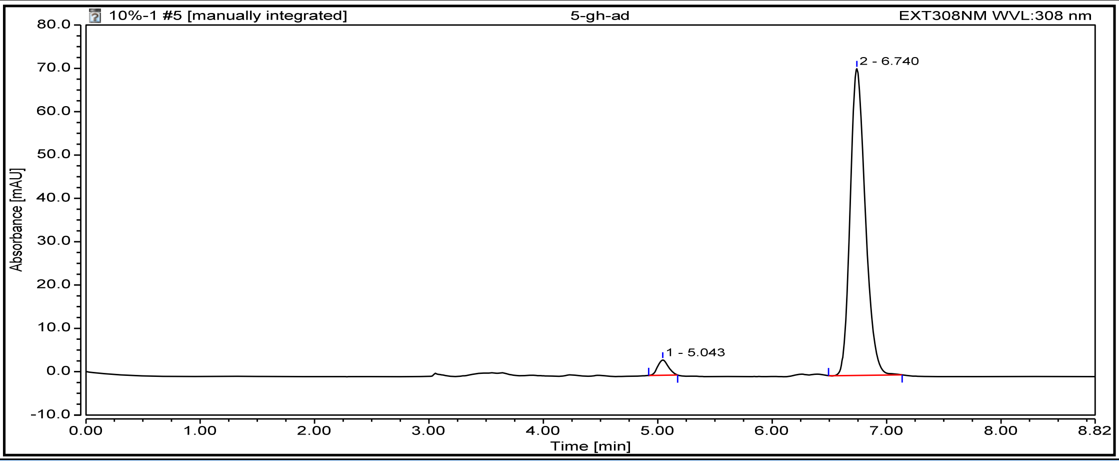

Integration Results					
No.	Peak Name	Retention Time<br>min	Area<br>mAU*min	Relative Area<br>%	Amount<br>n.a.
1		5.043	0.380	3.37	n.a.
2		6.740	10.880	96.63	n.a.
**Total:**			**11.260**	**100.00**	

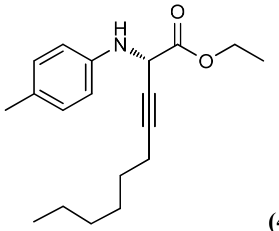

(4a)

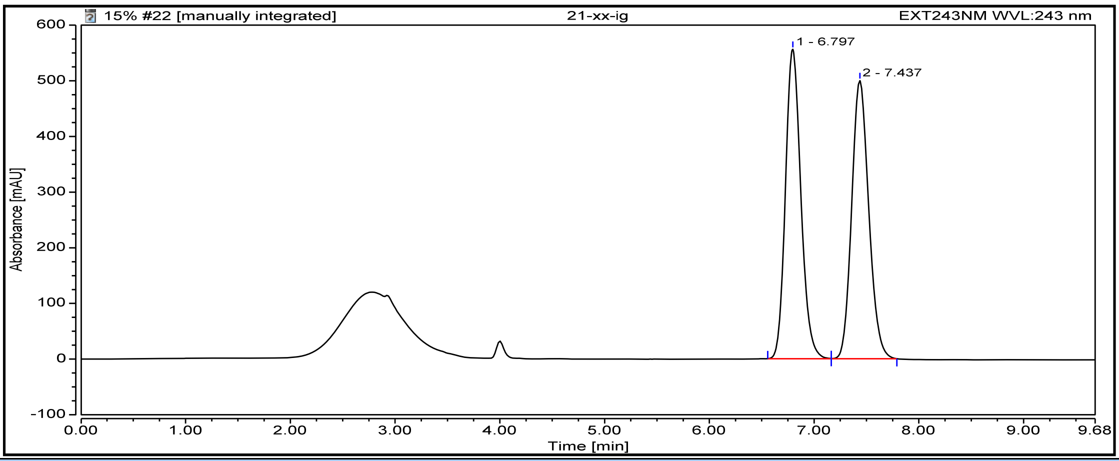

**Integration Results**					
No.	Peak Name	Retention Time<br>min	Area<br>mAU*min	Relative Area<br>%	Amount<br>n.a.
1		6.797	94.732	50.09	n.a.
2		7.437	94.405	49.91	n.a.
**Total:**			**189.137**	**100.00**	

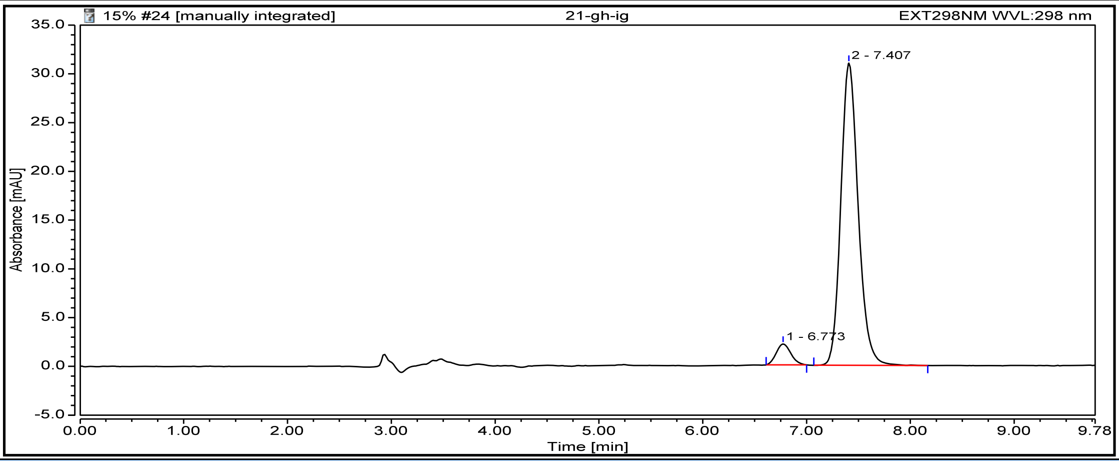

**Integration Results**					
No.	Peak Name	Retention Time<br>min	Area<br>mAU*min	Relative Area<br>%	Amount<br>n.a.
1		6.773	0.348	5.48	n.a.
2		7.407	6.006	94.52	n.a.
**Total:**			**6.355**	**100.00**	

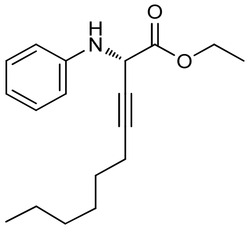

(4b)

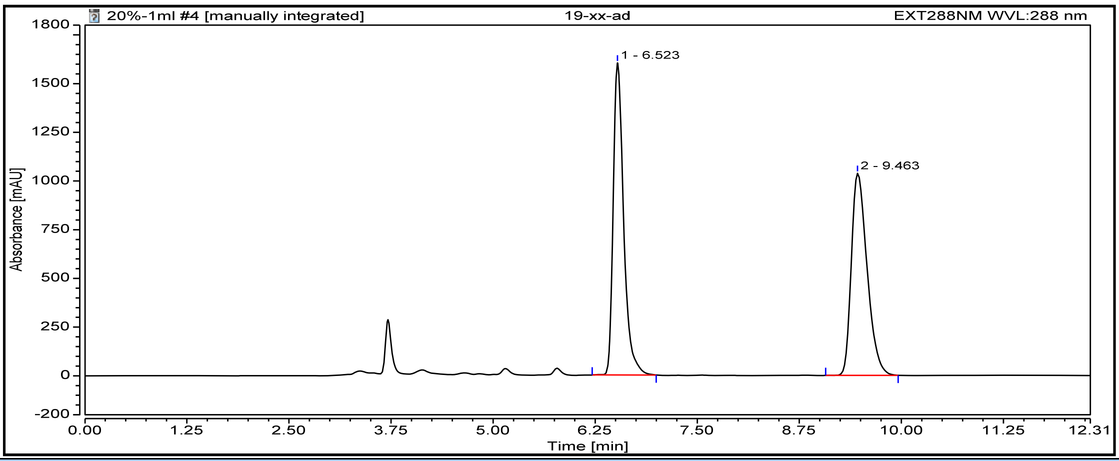

Integration Results					
No.	Peak Name	Retention Time<br>min	Area<br>mAU*min	Relative Area<br>%	Amount<br>n.a.
1		6.523	238.444	50.66	n.a.
2		9.463	232.242	49.34	n.a.
**Total:**			**470.686**	**100.00**	

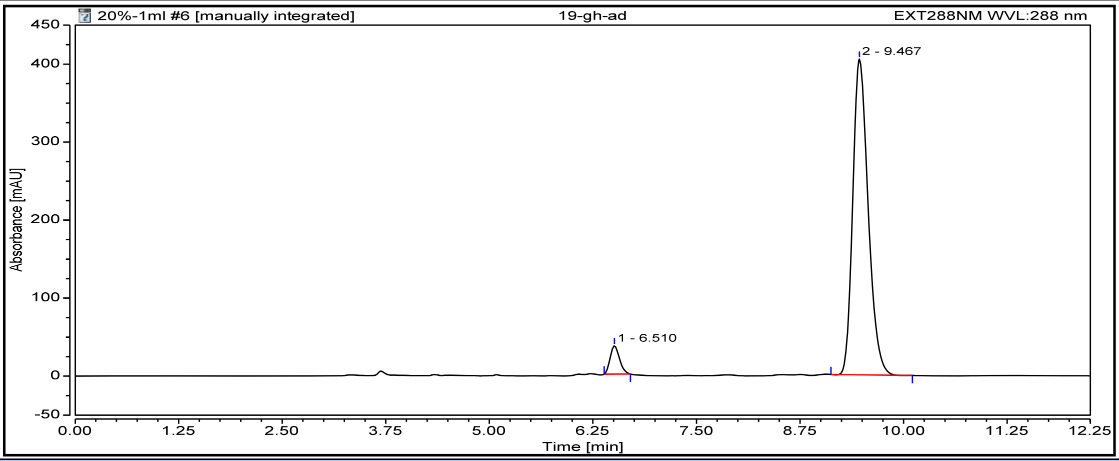

Integration Results					
No.	Peak Name	Retention Time<br>min	Area<br>mAU*min	Relative Area<br>%	Amount<br>n.a.
1		6.510	4.744	5.16	n.a.
2		9.467	87.269	94.84	n.a.
**Total:**			**92.013**	**100.00**	

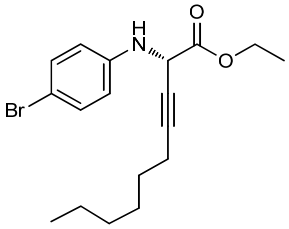

(4c)

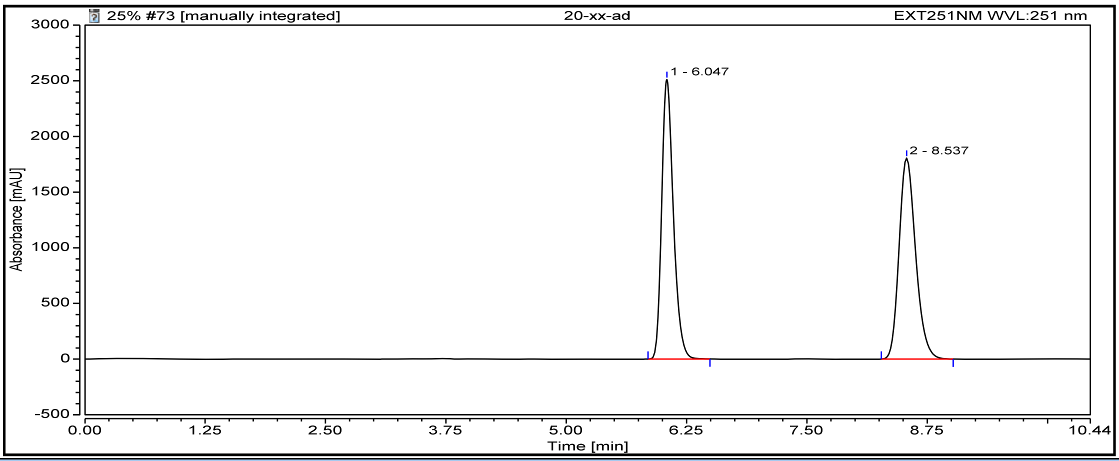

**Integration Results**					
No.	Peak Name	Retention Time<br>min	Area<br>mAU*min	Relative Area<br>%	Amount<br>n.a.
1		6.047	353.242	49.64	n.a.
2		8.537	358.339	50.36	n.a.
**Total:**			**711.581**	**100.00**	

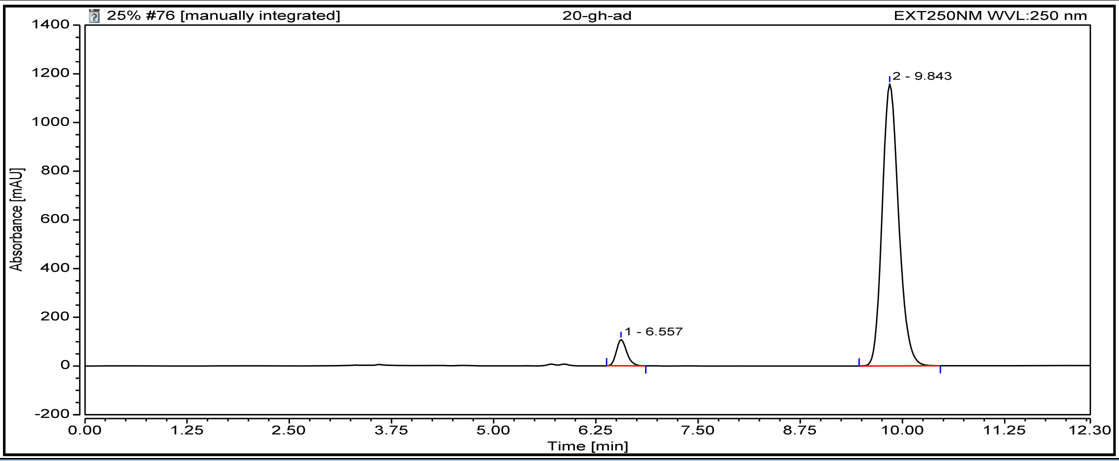

**Integration Results**					
No.	Peak Name	Retention Time<br>min	Area<br>mAU*min	Relative Area<br>%	Amount<br>n.a.
1		6.557	15.903	5.55	n.a.
2		9.843	270.789	94.45	n.a.
**Total:**			**286.692**	**100.00**	

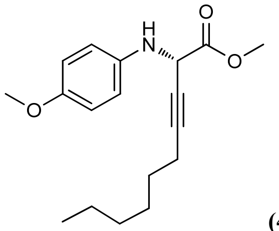

(4d)

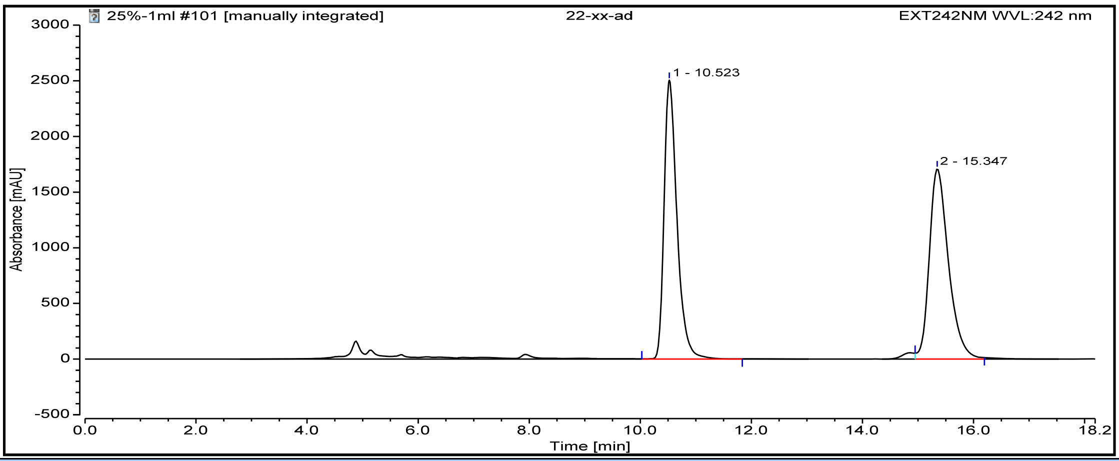

Integration Results					
No.	Peak Name	Retention Time<br>min	Area<br>mAU*min	Relative Area<br>%	Amount<br>n.a.
1		10.523	674.270	49.71	n.a.
2		15.347	682.243	50.29	n.a.
**Total:**			**1356.514**	**100.00**	

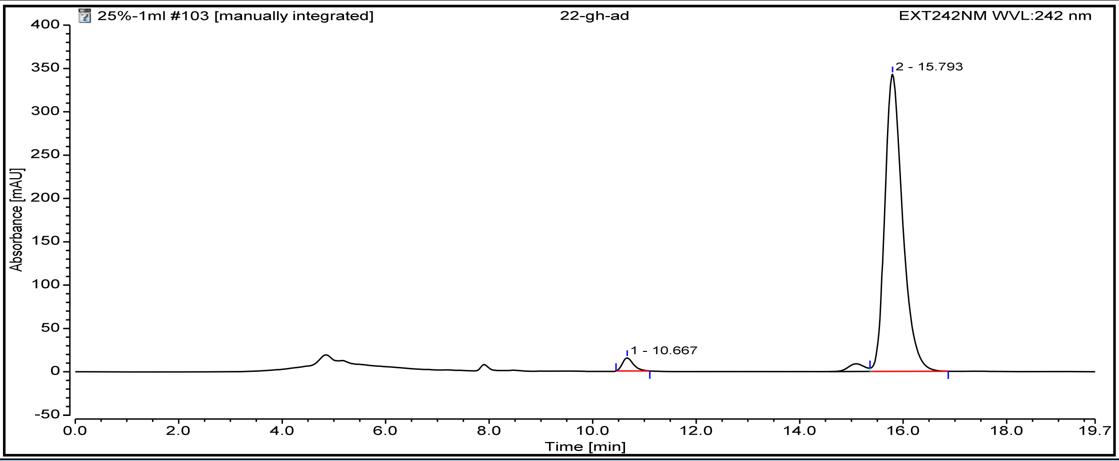

Integration Results					
No.	Peak Name	Retention Time<br>min	Area<br>mAU*min	Relative Area<br>%	Amount<br>n.a.
1		10.667	3.752	2.66	n.a.
2		15.793	137.030	97.34	n.a.
**Total:**			**140.782**	**100.00**	

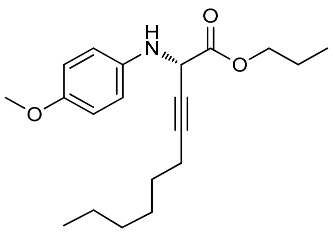

(4e)

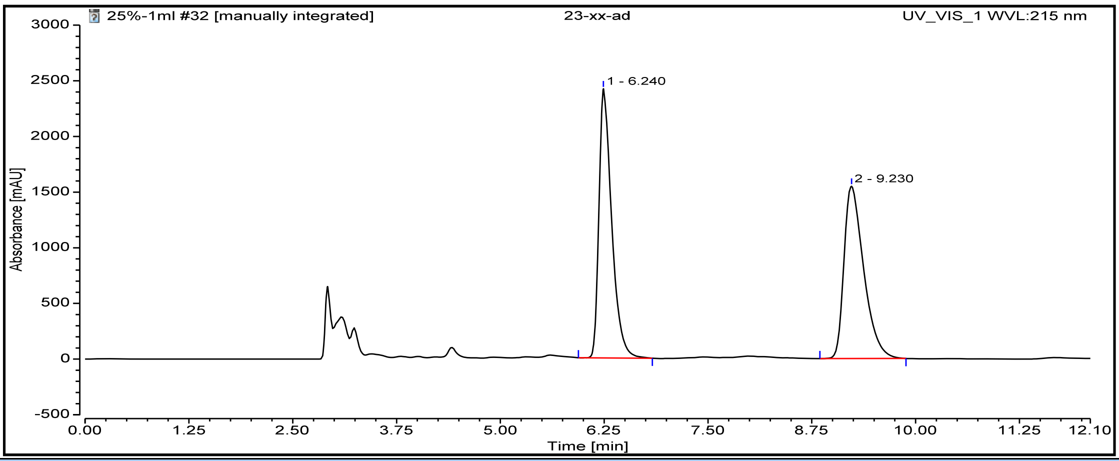

**Integration Results**					
No.	Peak Name	Retention Time<br>min	Area<br>mAU*min	Relative Area<br>%	Amount<br>n.a.
1		6.240	430.586	50.25	n.a.
2		9.230	426.326	49.75	n.a.
**Total:**			**856.913**	**100.00**	

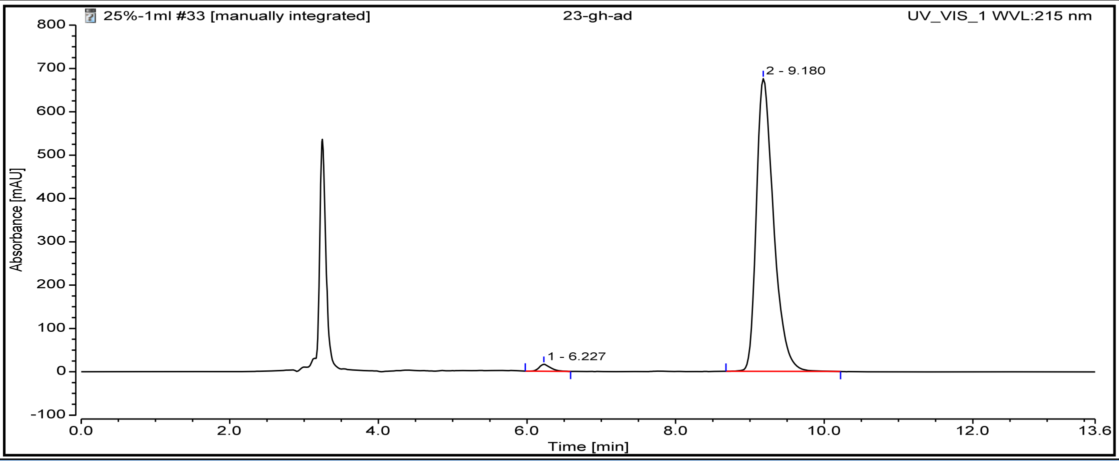

**Integration Results**					
No.	Peak Name	Retention Time<br>min	Area<br>mAU*min	Relative Area<br>%	Amount<br>n.a.
1		6.227	2.792	1.51	n.a.
2		9.180	182.115	98.49	n.a.
**Total:**			**184.907**	**100.00**	

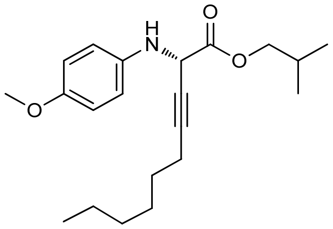

(4f)

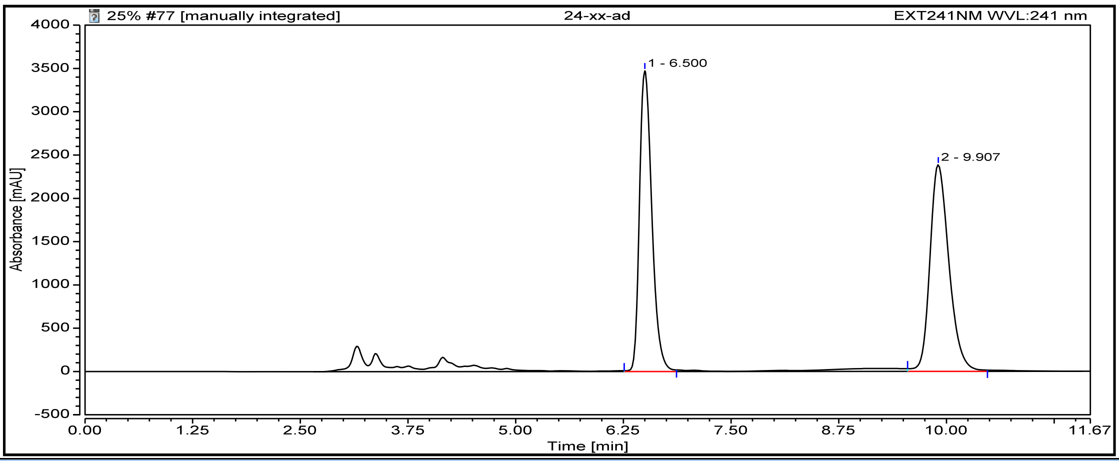

**Integration Results**					
No.	Peak Name	Retention Time<br>min	Area<br>mAU*min	Relative Area<br>%	Amount<br>n.a.
1		6.500	577.783	48.44	n.a.
2		9.907	615.086	51.56	n.a.
**Total:**			**1192.869**	**100.00**	

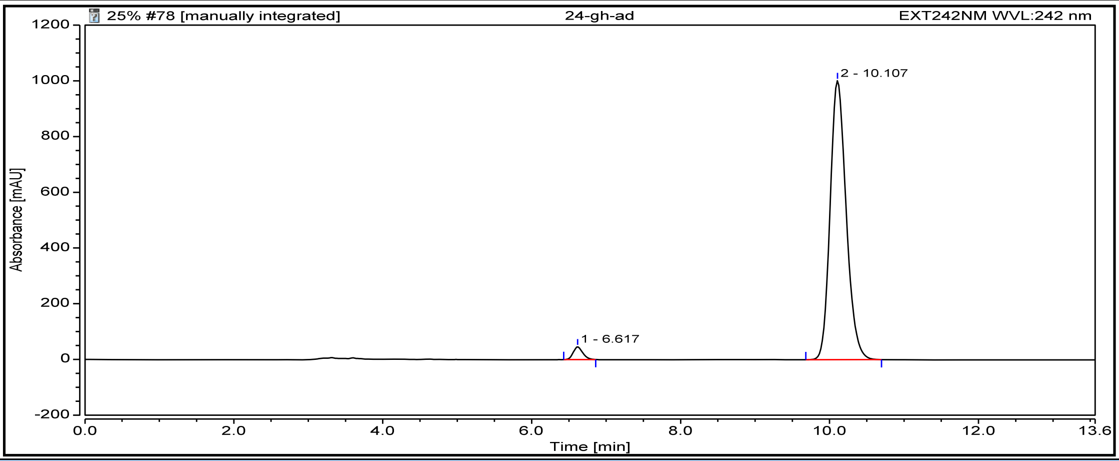

**Integration Results**					
No.	Peak Name	Retention Time<br>min	Area<br>mAU*min	Relative Area<br>%	Amount<br>n.a.
1		6.617	6.801	2.64	n.a.
2		10.107	250.531	97.36	n.a.
**Total:**			**257.332**	**100.00**	

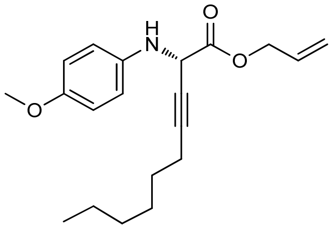

(4g)

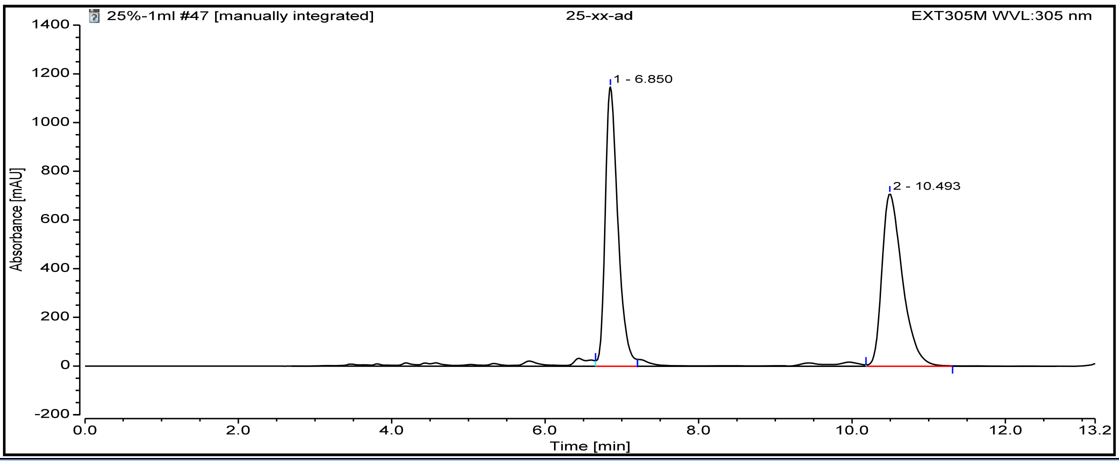

Integration Results					
No.	Peak Name	Retention Time<br>min	Area<br>mAU*min	Relative Area<br>%	Amount<br>n.a.
1		6.850	220.536	50.20	n.a.
2		10.493	218.811	49.80	n.a.
**Total:**			**439.347**	**100.00**	

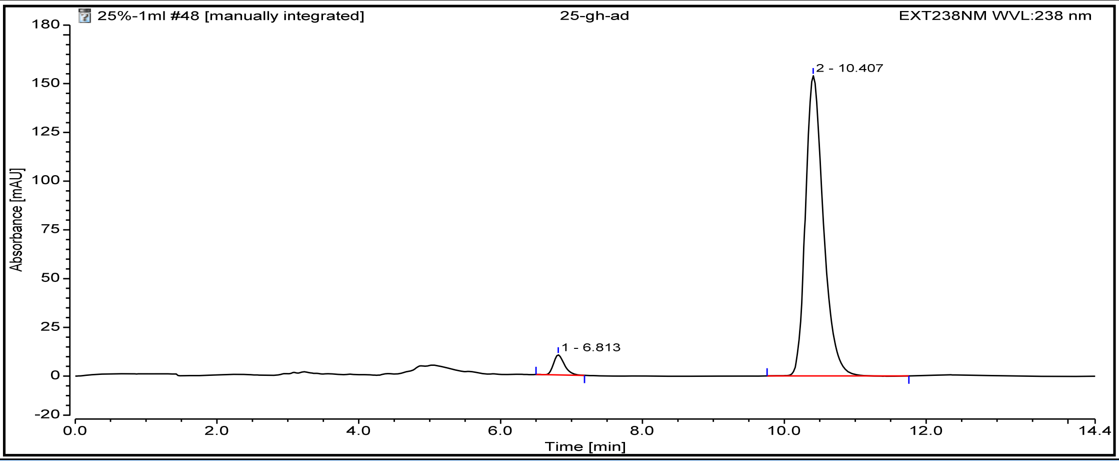

Integration Results					
No.	Peak Name	Retention Time<br>min	Area<br>mAU*min	Relative Area<br>%	Amount<br>n.a.
1		6.813	1.831	3.90	n.a.
2		10.407	45.122	96.10	n.a.
**Total:**			**46.953**	**100.00**	

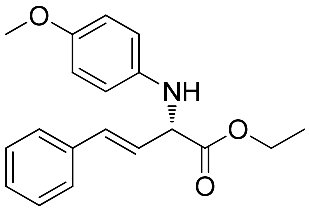

((*S*)-5)

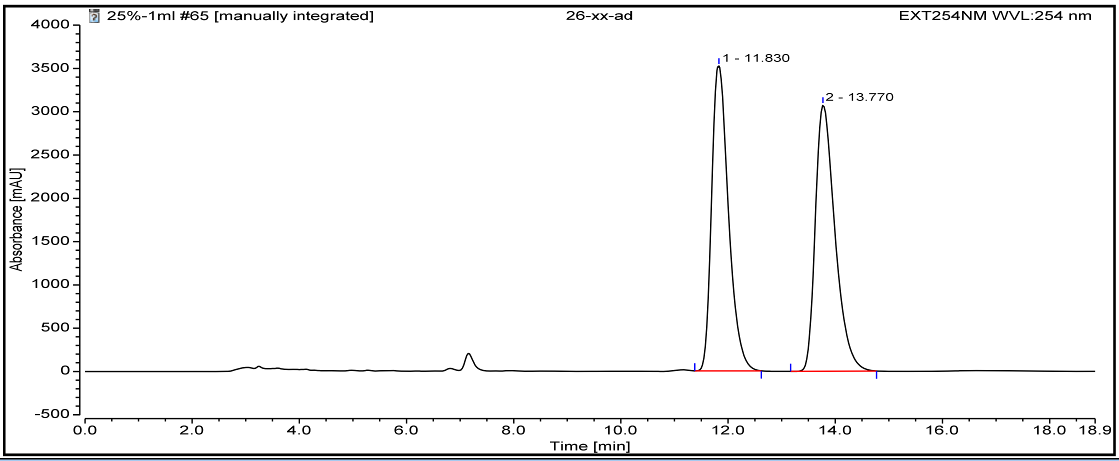

Integration Results

No.	Peak Name	Retention Time<br>min	Area<br>mAU*min	Relative Area<br>%	Amount<br>n.a.
1		11.830	1285.931	49.53	n.a.
2		13.770	1310.478	50.47	n.a.
**Total:**			**2596.409**	**100.00**	

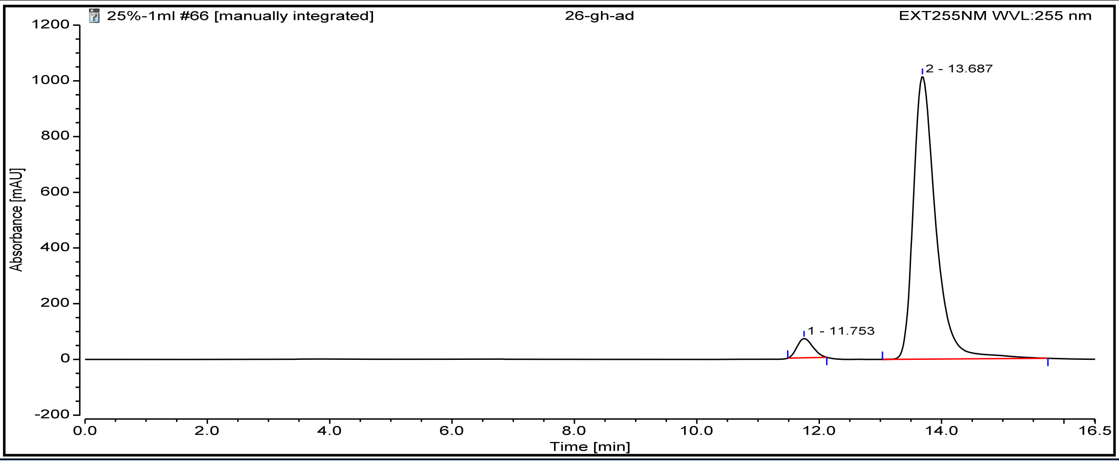

Integration Results

No.	Peak Name	Retention Time<br>min	Area<br>mAU*min	Relative Area<br>%	Amount<br>n.a.
1		11.753	20.691	4.57	n.a.
2		13.687	432.184	95.43	n.a.
**Total:**			**452.875**	**100.00**	
